# Small-molecule inhibitor of C‑terminal HSP90 dimerization modulates autophagy and functions synergistically with mTOR inhibition to kill cisplatin-resistant cancer cells

**DOI:** 10.1038/s41419-025-08330-4

**Published:** 2025-12-23

**Authors:** Céline David, Yadong Sun, Vitalij Woloschin, Melina Vogt, Niklas Dienstbier, Annabelle Friedrich, Karina S. Krings, David Schlütermann, Lena Berning, Beate Lungerich, Seda Akgün, María José Mendiburo, Christoph G. W. Gertzen, Arndt Borkhardt, Sebastian Wesselborg, Holger Gohlke, Sanil Bhatia, Thomas Kurz, Björn Stork

**Affiliations:** 1 Institute of Molecular Medicine I, Medical Faculty and University Hospital Düsseldorf, Heinrich Heine University, Düsseldorf, Germany; 2 Department of Urology, Weifang People’s Hospital, Shandong Second Medical University, Weifang, China; 3 Heinrich Heine University Düsseldorf, Faculty of Mathematics and Natural Sciences, Institute for Pharmaceutical and Medicinal Chemistry, Düsseldorf, Germany; 4 Department of Pediatric Oncology, Hematology and Clinical Immunology, Medical Faculty and University Hospital Düsseldorf, Heinrich Heine University, Düsseldorf, Germany; 5 Heinrich Heine University Düsseldorf, Center for Structural Studies (CSS), Düsseldorf, Germany; 6 Institute of Bio- and Geosciences (IBG-4: Bioinformatics), Forschungszentrum Jülich GmbH, Jülich, Germany

**Keywords:** Macroautophagy, Drug development

## Abstract

A major obstacle for the successful treatment of cancer is the presence or development of resistance mechanisms toward therapeutic intervention. In urothelial cancer, cisplatin-based regimens are still routinely employed, and multiple pathways contribute to chemoresistance. Since the identification of heat shock protein 90 (HSP90) as potential cancer target, various HSP90 inhibitors have been developed and evaluated in clinical trials. However, limited efficacy has been observed, mainly caused by dose-limiting toxicity and the concomitant induction of a cytoprotective heat shock response (HSR). To avoid this effect, inhibitors targeting the C-terminal domain (CTD) of HSP90 that do not elicit an HSR have been put forward. Additionally, the crosstalk between autophagy and HSP90 is currently being explored, since both processes work together in proteostasis, and the modulation of autophagy might be helpful in order to improve the efficacy of HSP90 inhibitors. We demonstrate that the second-generation small-molecule inhibitor VWK147 targeting HSP90 CTD dimerization induces cell death in both cisplatin-sensitive and cisplatin-resistant urothelial carcinoma cells. The treatment with VWK147 in these cells led to the destabilization of classical HSP90 client proteins without triggering an HSR. Additionally, we observe that VWK147 re-sensitizes resistant urothelial carcinoma cells to cisplatin and—in combination with mTOR inhibition—synergistically kills cisplatin-sensitive and -resistant cells, in contrast to what is observed upon treatment with the N-terminal domain-targeting HSP90 inhibitor 17-AAG. This synergy may be explained by VWK147-mediated inhibition of late autophagy events, and thus a blockade of autophagic flux. Finally, we also observed that VWK147 induces non-canonical LC3 lipidation, indicating that this compound exerts a broader effect on ion balance or pH of the endolysosomal system. VWK147 is a promising inhibitor that targets the C-terminal dimerization of HSP90 and simultaneously exhibits autophagy-modulating effects. This compound could potentially be an effective option for improving anti-cancer therapies and/or overcoming treatment resistance.

## Introduction

Heat shock proteins (HSPs) are molecular chaperones ensuring the function and stability of proteins. The HSP90 family includes five members, with either cytoplasmic (HSPC1/HSP90AA1; HSPC2/HSP90AA2; HSPC3/HSP90AB1), endoplasmic reticulum (HSPC4/HSP90B1), or mitochondrial localization (HSPC5/TRAP1) [1]. The general structure of HSP90 proteins includes (1) an N-terminal domain (NTD) harboring the ATPase activity and mediating co-chaperone binding, (2) a middle domain (MD) mediating client protein binding, co-chaperone binding and ATPase activation, and (3) a C-terminal domain (CTD) required for dimerization and co-chaperone binding [2]. The expression of heat shock proteins is upregulated in response to several cellular stress conditions, including raised temperature or oxidative stress.

HSP90 promotes cancer development by regulating several hallmarks of cancer, including sustained proliferative signaling, evasion of growth inhibitors, activation of invasion and metastasis, induction of angiogenesis, or resistance to cell death [2, 3]. Furthermore, HSP90 is overexpressed in several cancers, and high HSP90 expression has been identified as a marker of poor prognosis in lung cancer, esophageal cancer, urinary bladder cancer, melanoma, and leukemia [2]. Accordingly, HSP90 became an attractive target for anti-cancer therapy already in the early 1990’s [4]. So far, over 19 inhibitors targeting the HSP90 NTD have entered clinical trials, but none have been approved by the FDA as anti-cancer monotherapy [4]. Recently, the HSP90 NTD targeting inhibitor TAS-116 (Pimitespib) has been approved by the Japanese FDA for the treatment of gastrointestinal stromal tumors refractory to standard tyrosine kinase inhibitors [5]. Nevertheless, major disadvantages of NTD-targeting inhibitors are dose-limiting toxicities and the induction of a heat shock response (HSR) [4], during which HSP70 and other HSR indicators become upregulated, ultimately leading to cytoprotection of cancer cells and to increased resistance to HSP90 inhibition-induced cell death [6]. Next to NTD inhibitors, isoform-selective and CTD inhibitors have been described [4]. CTD-targeting inhibitors bind to the C-terminal ATP-binding site (e.g., novobiocin and derivatives), to co-chaperone binding sites, or interfere with HSP90 dimerization [4, 7–9]. With regard to dimerization-inhibiting compounds, peptidic inhibitors, a peptidomimetic inhibitor (aminoxyrone), and a small molecule inhibitor (5b or LSK82) based on a tripyrimidonamide scaffold have previously been reported [7–9]. In this study, we report the synthesis and evaluation of the second-generation small-molecule CTD dimerization inhibitor VWK147.

Generally, HSP90 exerts its chaperone function by forming complexes with the co-chaperone and the client in order to ensure their maturation and/or to maintain their stability [10, 11]. In turn, cellular proteostasis is mainly regulated by the ubiquitination-proteasome system (UPS) and (macro-)autophagy pathways, and both pathways contribute to the degradation of HSP90 clients [10]. However, autophagy not only mediates the degradation of HSP90 clients, but HSP90 associates and regulates several of the autophagy effector proteins. During the process of autophagy, proteins and organelles are transferred to lysosomes, where they become degraded and recycled. The initiation of autophagy is regulated by the ULK1 protein kinase complex and the VPS34/PIK3C3 lipid kinase complex. The core ULK1 complex consists of the Ser/Thr kinase unc-51 like autophagy activating kinase 1 (ULK1) and the associated proteins autophagy-related 13 (ATG13), ATG101, and focal adhesion kinase family interacting protein of 200 kD (FIP200) [12]. The ULK1 complex is regulated by the mammalian target of rapamycin (mTOR), which suppresses ULK1 kinase activity and thus inhibits autophagy via the phosphorylation of ULK1 at Ser758 (equivalent to Ser757 of mouse ULK1) [13, 14]. This site becomes dephosphorylated upon starvation or the pharmacological inhibition of mTOR, ultimately leading to ULK1 activation and the induction of autophagy [13, 14]. The second autophagy-inducing complex, the VPS34/PIK3C3 complex, is a class III phosphoinositide 3-kinase (PI3K) and contains the catalytic subunit vacuolar protein sorting 34 (VPS34)/phosphatidylinositol 3-kinase catalytic subunit type 3 (PIK3C3) and the associated proteins phosphoinositide-3-kinase regulatory subunit 4 (PIK3R4), ATG14, Beclin 1, and nuclear receptor binding factor 2 (NRBF2) [15]. The product of the VPS34/PIK3C3-catalyzed reaction, phosphatidylinositol 3-phosphate (PI3P), then recruits further downstream effector proteins such as double FYVE-containing protein 1 (DFCP1) and WD-repeat protein interacting with phosphoinositides (WIPI) proteins [16]. WIPI2 mediates the recruitment of the ATG12—ATG5-ATG16L1 complex, which is required for the lipidation of ATG8 proteins such as [microtubule associated protein 1] light chain 3 ([MAP1]LC3) [16]. During selective autophagy, ATG8 proteins bind to LC3-interacting region (LIR) motifs of the cargo or autophagy-receptors such as sequestosome 1 (SQSTM1)/p62 [17]. Next to canonical autophagy, non-canonical pathways have also been identified, which are characterized by the conjugation of ATG8s to single membranes (CASM) [18, 19]. On the molecular level, CASM appears to depend on the increased association of the lysosomal V-ATPase V0–V1 subunits, facilitating ATG16L1 recruitment through its C-terminal WD40 domain [20]. Thus, inhibition of the lysosomal V-ATPase with bafilomycin A_1_ (BafA_1_) leads to increased levels of lipidated LC3 during canonical autophagy, but inhibits LC3 lipidation during CASM [18].

In the last two decades, the function of autophagy in cancer has been addressed by various studies. It appears that autophagy plays an ambivalent role in cancer, i.e., prevention of early tumorigenesis versus the support of survival of established and metastasizing tumors [21]. Additionally, autophagic processes in the tumor microenvironment and/or immune-relevant cells, and non-canonical autophagy pathways such as CASM have to be considered in order to understand the role of autophagy in cancer pathogenesis [21]. Nevertheless, the identification and characterization of specific autophagy-modulating small molecules remains a promising approach for cancer therapy.

Urinary bladder cancer (BC) is the 9th most common cancer worldwide, with 613,791 new cases and 220,349 deaths in 2022 [22]. With 90% of these cases, urothelial carcinoma (UC) is the most common histologic type of BC [23]. Cisplatin-based combination treatments still represent an important adjuvant therapy, but their application and efficacy are frequently limited by the development of chemoresistance [24]. In bladder cancer, the PI3K/AKT/mTOR pathway is constitutively activated in up to 40% of tumors [25]. Furthermore, Pan et al. report an overexpression of both HSP90 and mTOR in bladder cancer cells [26]. Here, we demonstrate that the small molecule VWK147 induces cell death in cisplatin-sensitive and -resistant UC cells (UCCs), and re-sensitizes resistant cells to cisplatin treatment. In these UCCs, VWK147 treatment leads to destabilized client proteins without a concomitant heat shock response. Furthermore, we observe that VWK147—but not the N-terminal domain-targeting HSP90 inhibitor 17-AAG—in combination with mTOR inhibitors synergistically kills cisplatin-sensitive and -resistant UCCs. Finally, we show that VWK147 induces non-canonical LC3 lipidation but inhibits late stages of canonical autophagy.

## Results

### Synthesis of VWK147, a predicted CTD HSP90 inhibitor targeting the dimerization interface as an analog of LSK82

**Fig. 1:**
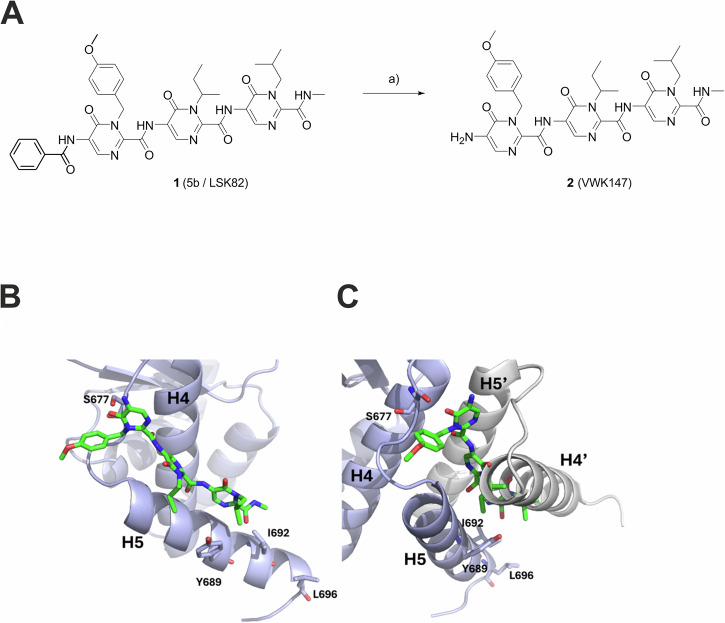
The structure and synthesis of VWK147, and its predicted binding mode at the CTD dimerization interface of an hHSP90 monomer. **A** Synthesis of tripyrimidonamide **2** (VWK147) from benzoyl-protected tripyrimidonamide **1** (LSK82): (a) NaOH, MeOH, 80 °C, 5 h, 40% yield. **B** **2** (VWK147) (green) is predicted to bind to the dimerization interface parallel to helix H5 of the hHSP90 protomer (light blue). The side chains of **2** (VWK147) are in the same order as those it mimics in helix H5’ of the other protomer, although the binding mode is slightly shifted towards the N-terminus of H5. There, it forms a hydrogen bond to S677 with its carbonyl group. **C** Upon projection of the binding mode onto the hHSP90 CTD dimer from PDB ID 3Q6M, it becomes evident that the binding mode of **2** (VWK147) effectively precludes H5’ and, notably, H4’ of the opposing protomer (depicted in gray) from engaging with the dimerization interface constituted by H4 and H5 of the initial protomer.

In 2022, Bhatia et al. reported the development of a first-in-class small-molecule inhibitor **1** (5b or LSK82, Fig. 1A, [8]) targeting the HSP90 CTD dimerization interface and based on a tripyrimidonamide scaffold. It was developed through structure-based molecular design, chemical synthesis, binding mode model prediction, assessment of the biochemical affinity, and evaluation of efficacy against therapy-resistant leukemia cells [8]. Drawbacks of this α-helix mimetic are the only moderate anti-cancer activity and its quite poor water solubility (aq. solubility). To simplify the chemical structure of **1** (LSK82) and to find out whether the benzoyl cap group is required for HSP90 inhibition and anti-cancer activity, we aimed at removing the benzoyl cap group of **1** to provide **2** (VWK147, Fig. 1A). Tripyrimidonamide **1** (LSK82) was synthesized as starting material from monomeric building blocks using a modular approach according to our previously published protocols [8, 27]. For the preparation of **2** (VWK147), the benzoyl cap group of **1** was removed by heating of **1** in a diluted aqueous solution of sodium hydroxide in methanol (Fig. 1). The side chains of the resulting cap-less tripyrimidonamide **2** (VWK147) mimic the same hot spots (Y689, I692, L696) of the CTD dimerization interface of HSP90 as **1**. The dimerization hot spots are located on α-helix H5, form a functional epitope, and account for most of the HSP90 dimerization energy. Since the C-terminal dimerization is essential for the chaperone activity of HSP90, in the following, **2** (VWK147) was studied by molecular modeling as well as in biochemical cell-free and cellular assays.

The binding mode of VWK147 at the dimerization interface of the HSP90 CTD was predicted by molecular docking using multiple X-ray structures of a monomeric hHSP90 protomer as a target. The docking results were generally similar in their orientation, although the conformation of helix H5 differed between the X-ray structures. They revealed that VWK147 interacts with helices H4 and H5 of the CTD in a manner that would prevent CTD dimerization (Fig. 1B, C). In the predicted binding mode, the side chains of VWK147 that mimic residues in helix H5’ of the other protomer are oriented accordingly, although the binding pose is slightly shifted towards the N-terminal end of H5, creating a large contact surface with H5. In comparison, the binding mode of LSK82 predicted previously is flipped, leading to less contact with helix H5 [8]. The difference between the two binding modes is likely a result of the missing benzoyl moiety in VWK147. As the conformation of the helices H4 and H5 could change in the absence of the other protomer, we also docked VWK147 to the representative structure of the largest cluster taken from 12 µs of accumulated MD simulations of full-length hHSP90. Here, we predominantly identify predicted binding modes in the cleft between the CTD and the middle domain, as were identified previously for LSK82 [8]. Taken together, the predicted binding modes of VWK147 are similar to those of LSK82 but VWK147 can form more interactions with H5 at the CTD dimerization interface.

### VWK147 is specific against HSP90 CTD and its co-chaperone function

**Fig. 2:**
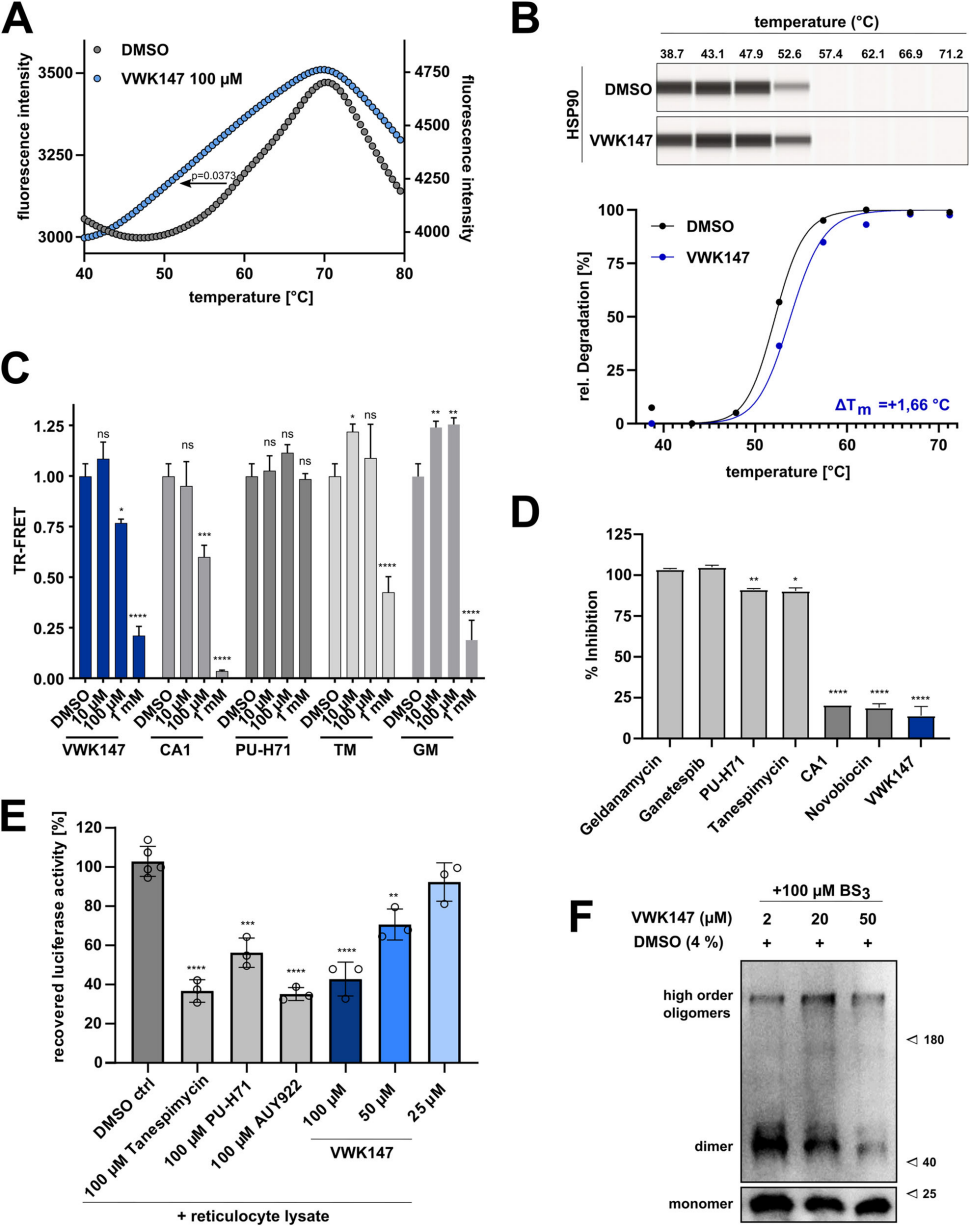
Specificity of VWK147 against HSP90 CTD and its co-chaperone function. **A** A cell-free thermal shift assay using recombinant HSP90α CTD protein with VWK147 displays destabilization of HSP90-CTD, indicated by a negative shift. **B** Cellular thermal shift assay (CETSA) in K562 leukemia cells displays stabilization of HSP90, indicated by a positive shift. **C**) VWK147 (1 mM) inhibits HSP90β, comparable to coumermycin A1 (CA1), indicated by a C-terminal FRET assay. **D** VWK147 (10 µM) is not binding to the N-terminal domain of HSP90, indicated by an N-terminal fluorescence polarization (FP) assay. **E** VWK147 inhibits the HSP90α chaperone function, comparable to Tanespimycin (TM), PU-H71 and AUY922, in the cell-free luciferase-refolding assay, where the incubation of the inhibitors prevented the HSP90-assisted refolding of denatured luciferase. **F** Recombinant HSP90α CTD was incubated with BS^3^ cross-linker at the indicated concentration with VWK147, followed by immunoblotting with the anti-HSP90 (AC88) antibody. VWK147 leads to a reduction of HSP90 dimers. Results are shown as means ± SD of three independent experiments. P values were either determined by a two-tailed unpaired t-test or two-way ANOVA. *p ≤ 0.05; **p ≤ 0.01; ***p ≤ 0.001; ****p ≤ 0.0001.

To investigate the specificity and selectivity of VWK147 against the CTD of HSP90, biochemical cell-free and cellular assays were performed [8]. First, we performed a cell-free thermal shift assay to evaluate the binding of VWK147 to recombinant HSP90α CTD. Incubation with VWK147 specifically destabilizes the dimeric HSP90α CTD (∆T_m_: −8.43 ± 2.75 °C) as indicated by a negative thermal shift (Fig. 2A). The thermostabilizing effect was also assessed in a cellular thermal shift assay (CETSA), which is based on ligand-induced stabilization of the target protein in living cells. The quantification of HSP90 was done using capillary-based western blotting and the treatment with VWK147 resulted in a higher amount of ligand-protected intracellular HSP90 with increasing temperature (Fig. 2B). Quantification of HSP90 degradation indicated a positive thermal shift (∆T_m_: 1.66 °C). To further investigate the specificity of VWK147 to the CTD of HSP90, a time-resolved fluorescence resonance energy transfer (TR-FRET) assay was performed. VWK147 disrupts the binding of Peptidyl prolyl isomerase D (PPID, CTD-specific co-chaperone) to the CTD of recombinant HSP90β similar to the C-terminal reference inhibitor coumermycin A1 (CA1) (Fig. 2C). To rule out interactions of VWK147 with the HSP90 NTD, a fluorescence polarization (FP) competitive assay was performed using FITC-labeled HSP90 NTD targeting reference inhibitor Geldanamycin (GM). Compared to HSP90-NTD targeting control inhibitors such as PU-H71, Tanespimycin (TM) and GM, VWK147 did not interact with the NTD of HSP90. This observation is consistent with other HSP90 CTD targeting reference inhibitors, such as CA1 and novobiocin (Fig. 2D). Next, we investigated the ability of VWK147 to inhibit HSP90 chaperone function in a cell-free luciferase refolding assay. Rabbit reticulocyte lysate was used as a source of HSP90, and it was demonstrated that VWK147 inhibited the recovered luciferase activity in a dose-dependent manner, displaying a comparable efficacy to the established HSP90 NTD targeting inhibitors TM, PU-H71, and AUY922 (Fig. 2E). To investigate the effect of VWK147 on the dissociation of the HSP90α CTD, a cell-free assay was conducted using the amine-reactive cross-linker BS^3^ to capture the oligomeric states of HSP90. Increasing concentrations of VWK147 resulted in a reduction of HSP90 CTD dimers, thereby inhibiting the function of HSP90, which is dependent on dimerization (Fig. 2F). Collectively, we confirmed that the specificity and selectivity of VWK147 against the CTD of HSP90 and its co-chaperone function are comparable to its predecessor, LSK82.

### VWK147 destabilizes HSP90 client proteins in urothelial carcinoma cells but does not induce a heat shock response

**Fig. 3:**
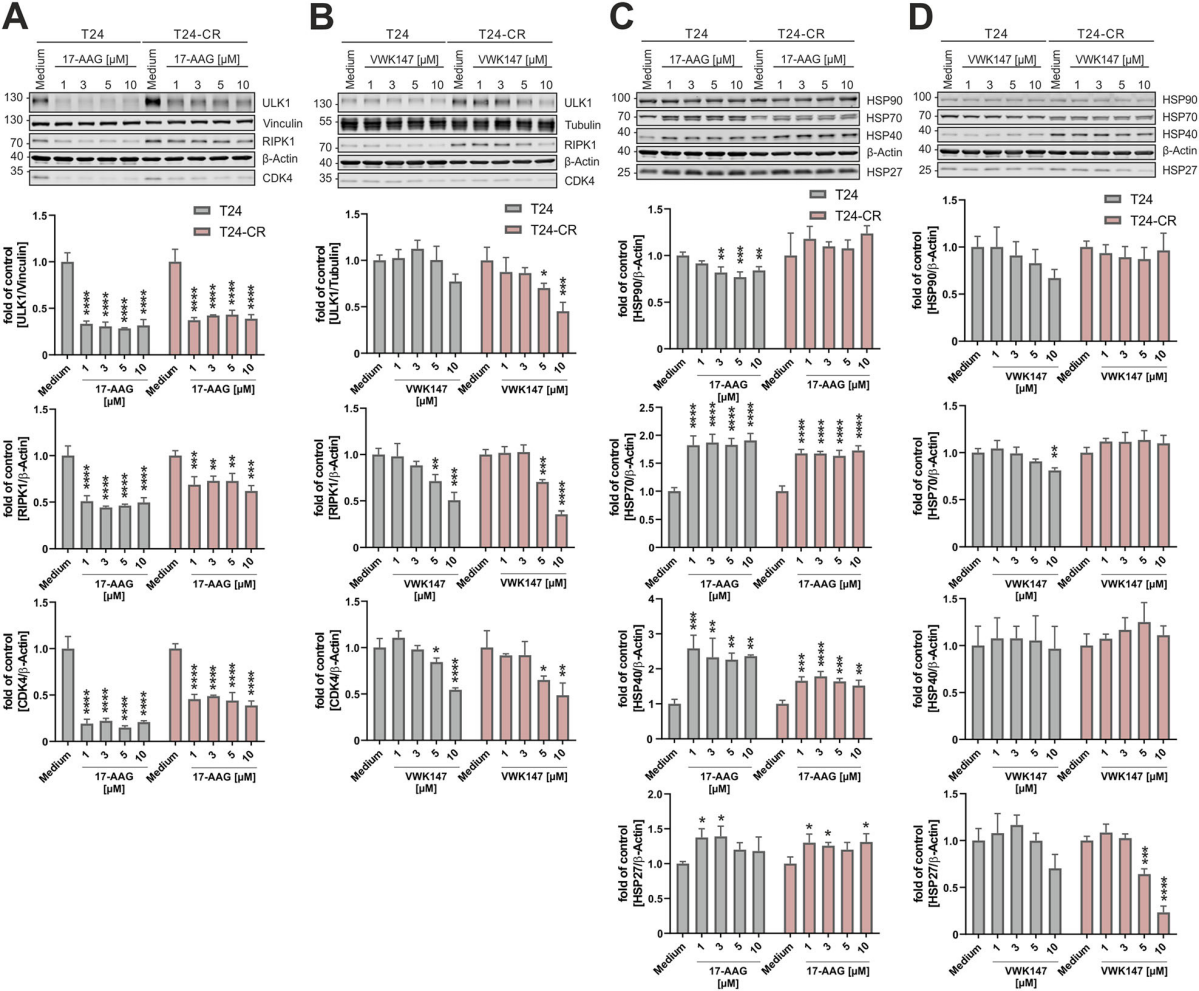
VWK147 destabilizes HSP90 clients but does not induce a heat-shock response. **A**–**D** T24 and T24-CR urothelial carcinoma cells were treated with the indicated concentrations of 17-AAG or VWK147 for 6 h. After treatment, the cells were lysed, and cellular lysates were immunoblotted for ULK1, RIPK1, CDK4, vinculin, tubulin, β-Actin, HSP90, HSP70, HSP40, and HSP27. One representative immunoblot is shown. The quantifications of indicated ratios are from three independent experiments (means + SD). P values were determined by ordinary one-way ANOVA with Dunnett´s multiple comparisons test (comparison to the solvent control of the respective cell line). *p ≤0.05; **p ≤0.01; ***p ≤ 0.001; ****p ≤ 0.0001.

Next, we aimed to confirm VWK147-mediated HSP90 inhibition in urothelial carcinoma cell (UCC) model systems. For that, we employed cisplatin-sensitive and -resistant sublines of the UCCs 253J and T24, respectively (Fig. S1). We treated the UCCs with the N-terminal HSP90 inhibitor 17-AAG or VWK147 and monitored protein levels of the HSP90 client proteins ULK1 [28], RIPK1 [29], and CDK4 [30], respectively. ULK1 is an autophagy-inducing kinase, RIPK1 mediates signaling downstream of the TNF-α receptor, and CDK4 regulates cellular transition into S-phase during cell cycle. The levels of the client proteins were reduced upon treatment with either HSP90 inhibitor (Fig. 3A, B and Fig. S2A, B). We also monitored the induction of a potential heat shock response upon treatment through the detection of HSP27, HSP40 and HSP70 levels by immunoblotting. 17-AAG significantly increased the levels of these heat shock proteins, whereas this was not the case for VWK147 (Fig. 3C, D and Fig. S2C, D). These data suggest that VWK147 destabilizes HSP90 client proteins without the concomitant induction of heat shock response.

### VWK147 induces cell death in UCCs

**Fig. 4:**
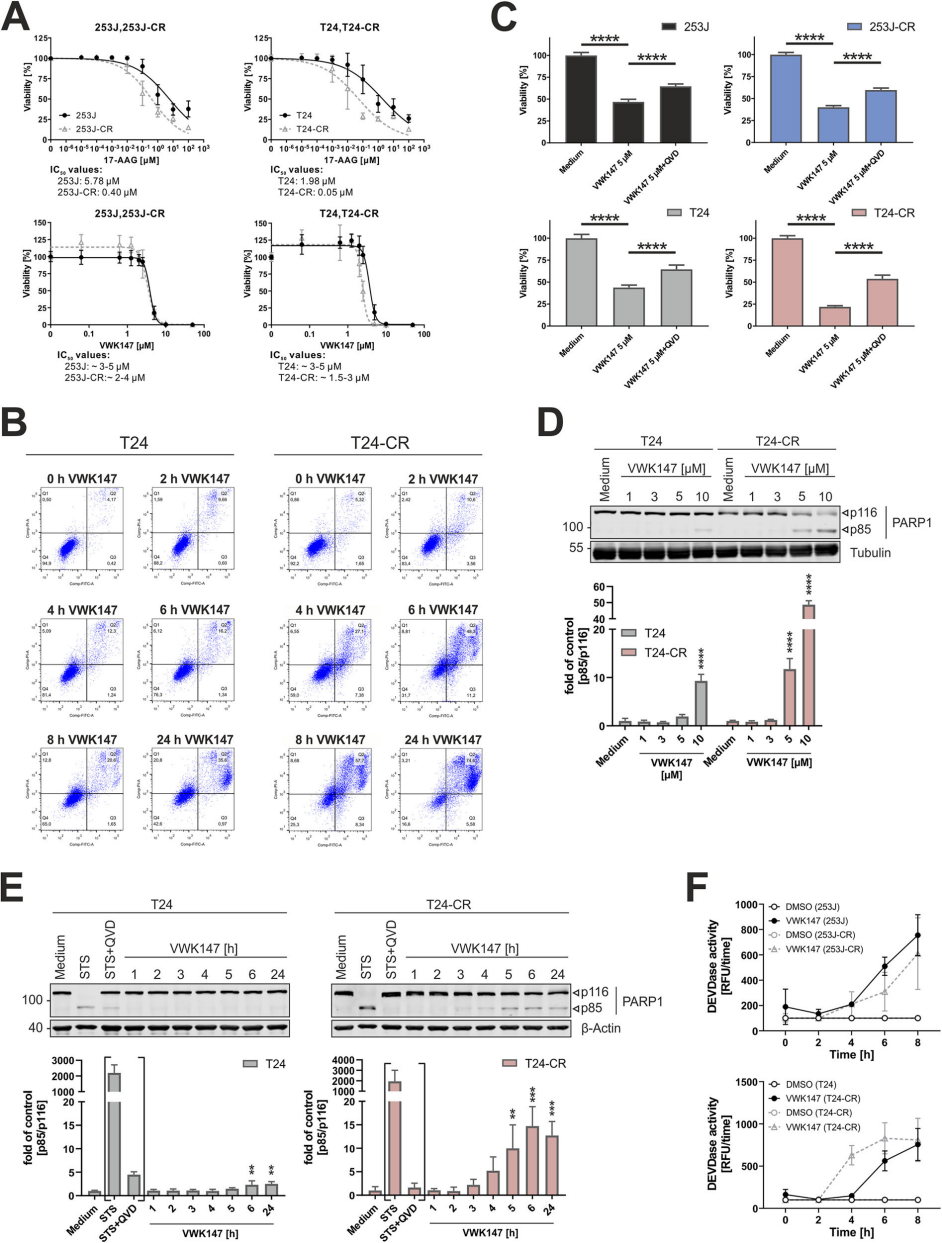
VWK147 reduces cell viability in cisplatin-sensitive and -resistant bladder carcinoma cells, partly by the induction of apoptosis. **A** 253J, 253J-CR, T24 and T24-CR urothelial carcinoma cells were treated with indicated concentrations of 17-AAG or VWK147 for 72 h. After treatment, cell viability was measured using Alamar Blue assay (17-AAG) or CellTiter-Glo (VWK147). Results are shown as means ± SD of n ≥ 3 independent experiments performed in triplicates for each treatment. Please note that—due to the steep decline in cell viability—concentration ranges are specified as IC_50_ for VWK147. **B** T24 and T24-CR urothelial carcinoma cells were treated with 10 µM VWK147 for the indicated periods of time. DMSO was used as a solvent control. After treatment, the cells were collected, stained with FITC-Annexin V and propidium iodide (PI) and analyzed by flow cytometry. One representative experiment is shown as dot plots. The quadrant Q4 represents living cells (Annexin V and PI negative), the quadrant Q3 early apoptotic cells (Annexin V positive, PI negative), the quadrant Q2 late apoptotic cells (Annexin V and PI positive) and the quadrant Q1 necrotic or dead cells (Annexin V negative and PI positive). Three independent experiments were performed. **C** 253 J, 253J-CR, T24 and T24-CR cells were treated with 5 µM VWK147 ± 20 µM QVD (caspase inhibitor) for 24 h. After treatment, cell viability was measured using MTT assay. Results are shown as means + SD of three independent experiments performed in triplicates for each treatment. P values were determined by ordinary one-way ANOVA with Tukey´s post hoc test. ****p ≤ 0.0001 **D** T24 and T24-CR urothelial carcinoma cells were treated with the indicated concentrations of VWK147 for 6 h. After treatment, the cells were lysed, and cellular lysates were immunoblotted for PARP1 and tubulin. One representative immunoblot is shown. The quantifications of cleaved PARP1 are from three independent experiments (means + SD). P values were determined by ordinary one-way ANOVA with Dunnett’s multiple comparisons test (comparison to the solvent control of the respective cell line). ****p ≤ 0.0001. **E** T24 and T24-CR urothelial carcinoma cells were treated with 5 µM VWK147 for the indicated periods of time or with 2.5 µM staurosporine (STS) ± 10 µM QVD for 24 h. After treatment, the cells were lysed, and cellular lysates were immunoblotted for PARP1 and β-Actin. One representative immunoblot is shown. The quantifications of cleaved PARP1 are from three independent experiments (means + SD). P values were determined by ordinary one-way ANOVA with Dunnett’s multiple comparisons test (comparison to the solvent control of the respective cell line). Brackets indicate the exclusion of STS and STS + QVD in the significance test. **p ≤0.01; ***p ≤0.001. **F** 253 J, 253J-CR, T24 and T24-CR urothelial carcinoma cells were treated with 5 µM VWK147 for the indicated periods of time. After treatment, the cells were lysed and mixed with the profluorescent caspase substrate Ac-DEVD-AMC. The kinetics of AMC release were determined as a surrogate marker for caspase-3 activation by measuring AMC fluorescence intensity. Results are shown as means + SD of three independent experiments performed in duplicates for each treatment.

Next, we investigated the effect of VWK147-mediated HSP90 inhibition on cell viability in our UCC model systems. Both 17-AAG and VWK147 reduced cell viability in UCCs. For 17-AAG, we observed lower IC_50_ values for the cisplatin-resistant sublines when compared to sensitive sublines (Fig. 4A, upper panels). For VWK147, we did not observe differences for the 253J pair, and a slightly lower IC_50_ value for the cisplatin-resistant T24 subline (Fig. 4A, lower panels). We also performed FITC-Annexin V/PI-based flow cytometry in order to characterize the mode of cell death (Fig. 4B and Fig. S3A–C). In contrast to the positive control staurosporine, which induced clear early apoptotic events, VWK147 appeared to induce a cell death with both apoptotic and necrotic properties, as reflected from a rather direct “diagonal” shift from viable to late apoptotic/necrotic cells (Fig. 4B). Since these data suggested that VWK147 can at least partially induce apoptosis, we wanted to confirm this by using the caspase inhibitor QVD. Indeed, QVD partially reduced the VWK147-mediated reduction of cell viability, but did not completely abolish the observed effect (Fig. 4C). The partial pro-apoptotic effects of VWK147 were confirmed by the concentration- (Fig. 4D) and time-dependent (Fig. 4E) detection of cleaved PARP1, and by monitoring caspase-3 activation (Fig. 4F). With regard to caspase activation by VWK147, no major difference was found between the sensitive and resistant 253 J sublines. For the T24 pair, we observed slightly earlier caspase-3 activation in cisplatin-resistant cells, confirming the observations of the cell viability assay shown in Fig. 4A. However, the magnitude of caspase-3 activation was similar in both T24 sublines. Taken together, VWK147 induces apoptosis and presumably additional forms of cell death in cisplatin-sensitive and -resistant UCCs.

### VWK147 sensitizes resistant cells to cisplatin treatment and acts synergistically with mTOR inhibition

**Fig. 5:**
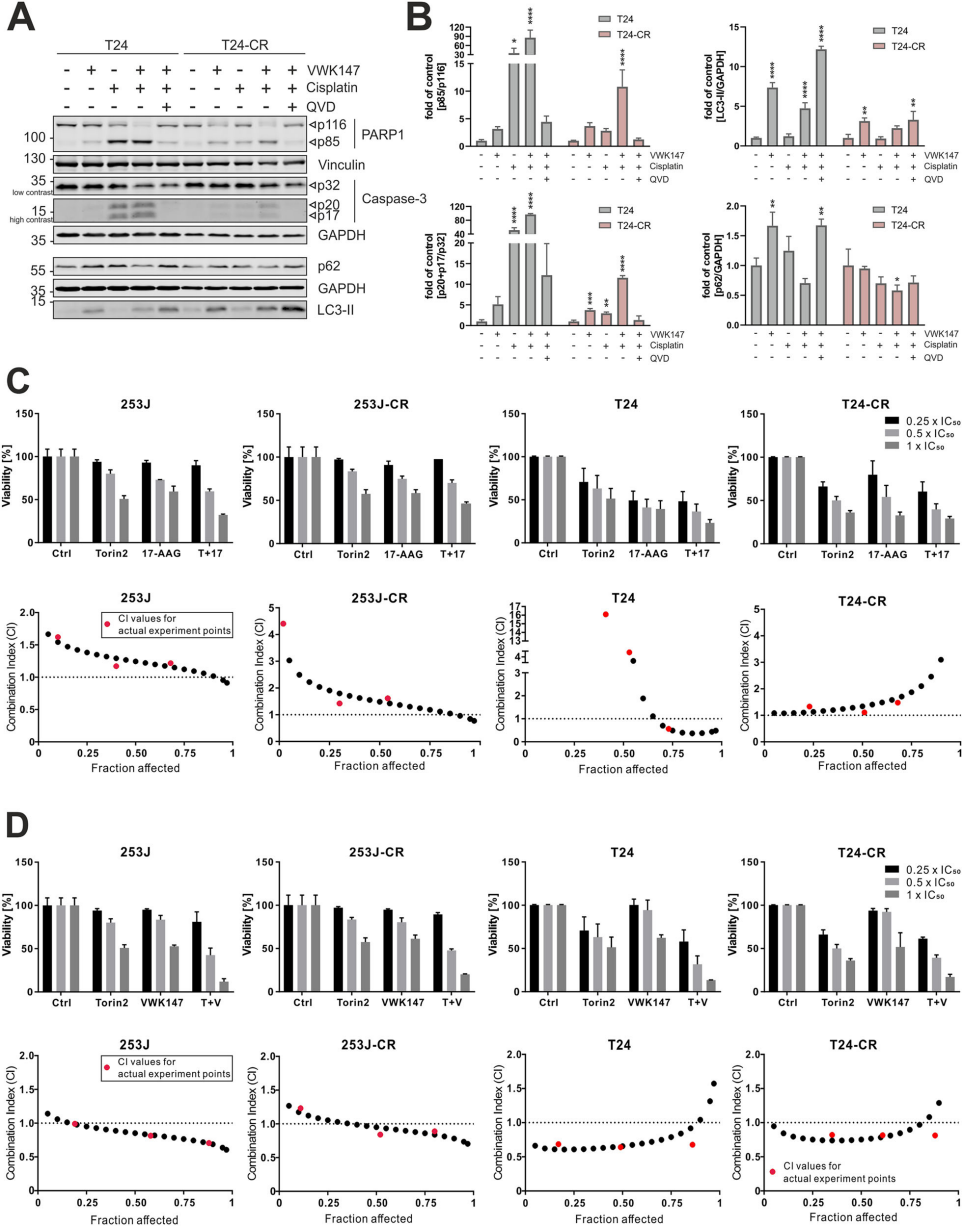
VWK147 sensitizes T24-CR cells to cisplatin-induced apoptosis and acts synergistically with mTOR inhibition. **A** T24 and T24-CR urothelial carcinoma cells were treated with 24 h-IC_50_ concentrations of VWK147 (~10 µM and 5 µM, respectively) and cisplatin (~8 µg/ml and 87 µg/ml, respectively) alone or in combination in absence or presence of 10 µM QVD. After 24 h, the cells were lysed, and cellular lysates were immunoblotted for PARP1, Vinculin, Caspase-3, p62, LC3 and GAPDH. One representative immunoblot is shown. **B** The quantifications of cleaved PARP1, cleaved caspase-3, LC3-II or p62 are from three independent experiments (means + SD). P values were determined by ordinary one-way ANOVA with Dunnett’s multiple comparisons test (comparison to the solvent control of the respective cell line). *p ≤ 0.05; **p ≤ 0.01; ***p ≤ 0.001; ****p ≤ 0.0001. **C**, **D** 253 J, 253J-CR, T24 and T24-CR cells were treated with Torin2, 17-AAG, VWK147, or Torin2 combined with VWK147 or 17-AAG (0.25×, 0.5× and 1 × IC_50_) for 72 h. The approximate IC_50_ values of each compound were determined based on the previous mono-treatment. The cell viability was measured using Alamar Blue assay. Viability results are depicted in bar diagrams as means + SD of three independent experiments performed in triplicates for each treatment. The combination index values were calculated using CompuSyn [33]. The actual experiment points are indicated as red dots whereas simulation dots processed by CompuSyn are indicated as black dots. The combination effects of Torin2 and 17-AAG or VWK147 were determined synergistic (CI <1), additive (CI = 1) and antagonistic (CI >1).

Next, we tested whether VWK147 sensitizes the resistant subline of T24 cells towards cisplatin treatment. For this, we treated both sublines either with cisplatin or VWK147 alone or in combination, and monitored PARP1 and caspase-3 cleavage by immunoblotting. For both sensitive and resistant T24 sublines, VWK147 treatment significantly increased cisplatin-induced PARP1 and caspase-3 cleavage (Fig. 5A, B), indicating that VWK147 can sensitize resistant cancer cells for cisplatin treatment.

The PI3K-AKT-mTOR pathway is a central target for anti-cancer therapy. Several mTOR inhibitors have been assessed in clinical trials. However, these stand-alone approaches revealed limited efficacy due to several reasons, e.g., induction of feedback survival loops, importance of mTOR signaling for healthy tissue, or the induction of autophagy [31]. Inhibitors of mTOR were also assessed in several combinatorial therapy approaches, and in recent years, it has been demonstrated that the combination of HSP90 and mTOR inhibitors can produce a synergistic anticancer effect in several tumors [26]. mTOR inhibition interrupts survival signaling and attenuates HSF1-driven heat shock response [32], while HSP90 inhibition destabilizes AKT and mTOR, thus reducing feedback loops and causing stronger anticancer activity. Based on this, we decided to analyze whether co-inhibition of mTOR and HSP90 leads to an increase in the cytotoxicity observed in VWK147-treated cisplatin-resistant cells. First, we tested the mTOR inhibitor Torin2 in the UCCs and confirmed its efficacy by detecting reduced phosphorylation of mTOR Ser2448 and the mTOR substrate site Ser758 in ULK1 (Fig. S4A, B). ULK1 Ser758 is an inhibitory site, and, accordingly, Torin2 treatment resulted in increased phosphorylation of the ULK1 substrate ATG14 at Ser29 (Fig. S4A and S4B). To obtain IC_50_ values for combination analyses, we treated cisplatin-sensitive and -resistant 253J and T24 cells with different concentrations of Torin2. The inhibitor efficiently reduced cell viability, with low IC_50_ values especially in the cisplatin-resistant sublines (Fig. S4C). To investigate whether Torin2 and HSP90 co-inhibition exhibits a synergistic effect on cytotoxicity, we performed isobologram analysis using the Chou-Talalay method [33]. The combination of Torin2 and 17-AAG was not synergistic (Fig. 5C), whereas this was clearly the case for VWK147 at most tested concentrations in all four UCC lines (Fig. 5D). To assess further clinical applications, we repeated the above described experiments with the mTOR inhibitor Temsirolimus, which is FDA/EMA-approved for advanced renal cell carcinoma and EMA-approved for mantle cell lymphoma [34]. We confirmed general functionality of Temsirolimus in 253J and T24 cells by immunoblotting for phospho-mTOR substrates as described above and by assessing Temsirolimus-induced reduction of cell viability (Fig. S5A, B). Similarly, we performed isobologram analysis using the combination of Temsirolimus and VWK147 (Fig. S6). Again, we observed synergy and/or additive effects for this combination.

Finally, we assessed the efficacy of VWK147 and its combination with mTOR inhibition in HBLAK cells, a spontaneously immortalized cell line derived from a primary culture of uroepithelial cells [35]. It has been suggested that these cells represent a preneoplastic stage of UCC [35]. We confirmed VWK147 functionality by immunoblotting for HSP90 client proteins (Fig. S7A) and Torin2/Temsirolimus functionality by immunoblotting for phospho-mTOR substrates (Fig. S7B). We also performed cell viability assays with all three compounds in HBLAK cells (Fig. S7C). In isobologram analyses, we observed rather additive effects with the combination of VWK147 and Torin2 (Fig. S8). For Temsirolimus, we observed slight synergistic effects for some experimental concentrations (Fig. S8). However, it needs to be noted that the IC_50_ value of Temsirolimus is higher in HBLAK than in 253J or T24 cells. Overall, synergistic effects appeared to be lower in HBLAK as compared to UCCs, indicating that synergy might depend on the neoplastic stage of the cell.

Collectively, mTOR inhibition and VWK147 treatment appear to synergistically kill UCCs.

### Forced expression of HSP70 reduces the cytotoxic effect of VWK147

Since we observed the induction of a heat shock response and increased HSP70 expression upon 17-AAG treatment, we hypothesized that this might explain the lack of synergism for Torin2 and 17-AAG. To test this, we exogenously overexpressed 3xFLAG-HSP70 in all four UCC sublines (Fig. S9A) and assessed cell viability upon 17-AAG or VWK147 treatment. Indeed, HSP70 overexpression counteracted the reduction in cell viability observed upon HSP90 inhibition in UCCs (Fig. S9B). Our results suggest that the synergism between VWK147 and mTOR inhibition can be at least partially caused by the absence of a heat shock response.

### VWK147 induces LC3-II accumulation and inhibits autophagosome-lysosome fusion

**Fig. 6:**
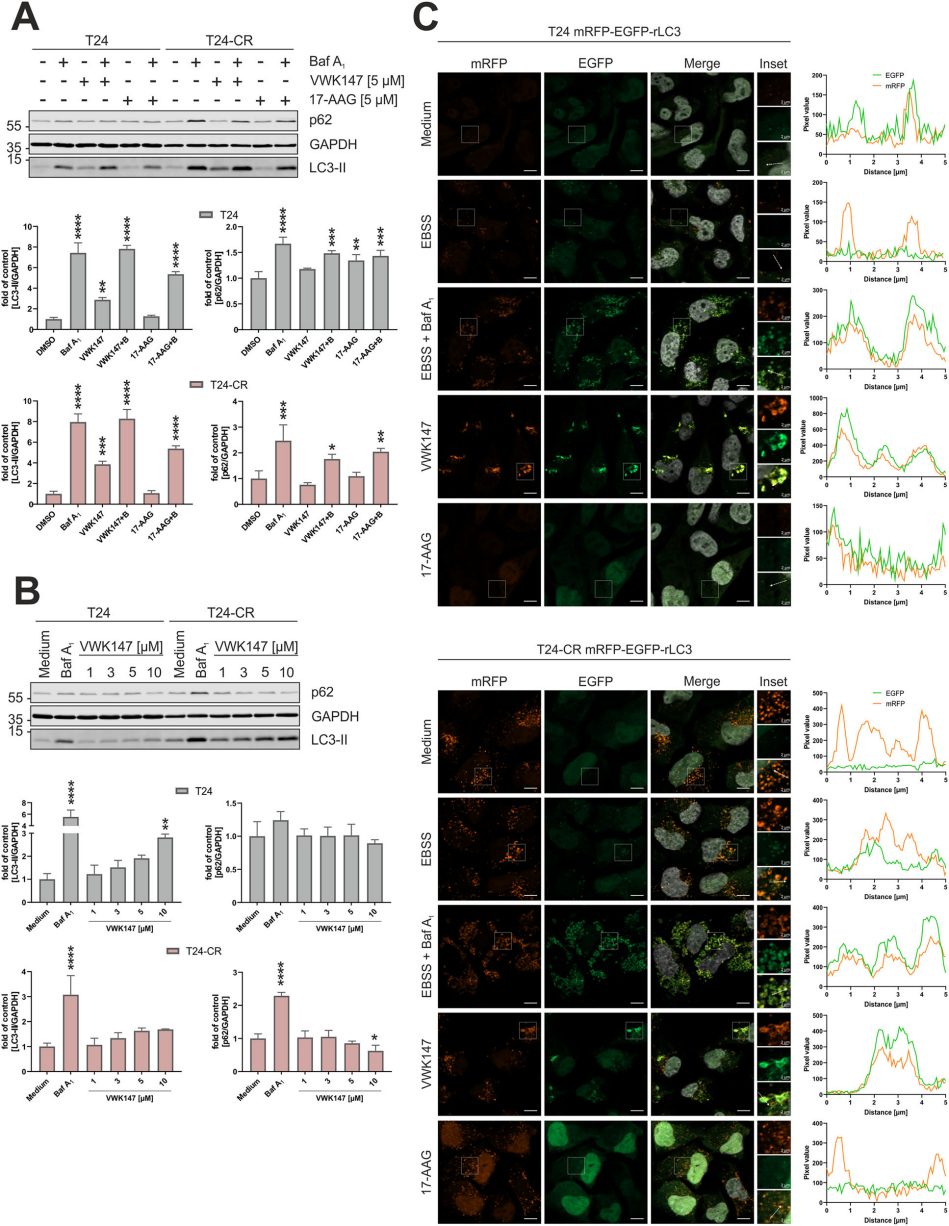
VWK147 inhibits canonical autophagy. **A** T24 and T24-CR urothelial carcinoma cells were treated with 5 µM VWK147 or 17-AAG in presence or absence of 20 nM bafilomycin A_1_ for 6 h. After treatment, the cells were lysed, and cellular lysates were immunoblotted for p62, LC3 and GAPDH. One representative immunoblot is shown. The quantifications of indicated ratios are from three independent experiments (means + SD). P values were determined by ordinary one-way ANOVA with Dunnett´s multiple comparisons test (comparison to the solvent control of the respective cell line). *p ≤ 0.05; **p ≤ 0.01; ***p ≤ 0.001; ****p ≤ 0.0001. **B** T24 and T24-CR urothelial carcinoma cells were treated with indicated concentrations of VWK147 or 20 nM bafilomycin A_1_ for 6 h. After treatment, the cells were lysed, and cellular lysates were immunoblotted for p62, LC3 and GAPDH. One representative immunoblot is shown. The quantifications of indicated ratios are from three independent experiments (means + SD). P values were determined by ordinary one-way ANOVA with Dunnett´s multiple comparisons test (comparison to the solvent control of the respective cell line). *p ≤ 0.05; **p ≤ 0.01; ****p ≤ 0.0001. **C** T24 and T24-CR urothelial carcinoma cells stably expressing mRFP-EGFP-rLC3 were grown on glass coverslips 1 or 2 days prior to treatment. Cells were treated with EBSS alone or in combination with 20 nM bafilomycin A_1_, 5 µM VWK147 or 17-AAG for 4 h. Imaging was performed using a Zeiss Axio Observer 7 fluorescence microscope equipped with a 40x/1.4 Oil DIC M27 Plan-Apochromat objective and ApoTome 2. Representative sections are depicted. Scale bars: 10 µm and 2 µm. The line graphs represent the pixel intensities of the areas indicated by the respective dashed white arrows shown in the insets.

Although the lack of a heat shock response upon treatment with VWK147 seems to be relevant for its synergism with Torin2, additional pathways affected by both VWK147 and mTOR inhibition might contribute to this phenomenon. As stated above, mTOR inhibition generally results in the activation of autophagy. In order to investigate whether VWK147 affects autophagy signaling, we analyzed autophagic flux by treating the T24 sublines with the HSP90 inhibitors in the presence or absence of bafilomycin A_1_ (BafA_1_) (Fig. 6A). BafA_1_ is an inhibitor of vacuolar H^+^-ATPases and blocks the lysosomal degradation of autophagosomes. VWK147 mono-treatment increased the levels of lipidated LC3 (LC3-II) in either subline, whereas this was not the case for 17-AAG, indicating that VWK147 affects autophagy signaling. In order to distinguish between autophagy-inducing or -inhibiting properties of VWK147, we compared the BafA_1_-treated cells. The combination of VWK147 and BafA_1_ did not significantly increase LC3-II levels compared to BafA_1_ treatment alone, indicating that VWK147 rather inhibits autophagic flux. We also investigated levels of the autophagy receptor p62/SQSTM1. Here, an accumulation was only evident in BafA_1_-treated cells, and the effects of either HSP90 inhibitor were rather negligible (Fig. 6A). VWK147-mediated LC3-II accumulation and unaffected p62 levels were also observed in a concentration-dependent manner (Fig. 6B).

In order to ultimately prove the autophagy-inhibiting properties of VWK147, we generated cisplatin-sensitive and -resistant T24 sublines stably expressing mRFP-EGFP-rLC3, and monitored autophagic flux by the detection of yellow puncta (mRFP/EGFP co-localization, representing autophagosomes) and red puncta (mRFP only, representing autolysosomes) using fluorescence microscopy (Fig. 6C) [36]. As a control for autophagy induction, we used a starvation medium (EBSS). Under starvation conditions, both yellow and red puncta were detectable, suggesting the presence of both autophagosomes and autolysosomes. In contrast, EBSS and BafA_1_ co-treatment abolished red-only dots, confirming the inhibited fusion of autophagosomes with lysosomes. These observations were similar in both cisplatin-sensitive and -resistant sublines, with the exception of rather increased red-only dots in the resistant cells, confirming our previously reported enhanced autophagic flux in cisplatin-resistant T24 [37]. Interestingly, VWK147 treatment resulted in an almost complete co-localization of GFP and RFP signals, again indicating that VWK147 interferes with the generation of autolysosomes. Of note, these co-localizing structures appeared to be more tube-like or vesicular aggregates/clusters rather than puncta. In contrast, 17-AAG treatment did not induce similar signals. Collectively, our data show that VWK147 induces LC3 accumulation and inhibits autophagosome-lysosome fusion, and that these effects are not shared by HSP90 inhibitors targeting the NTD.

### VWK147 induces CASM

**Fig. 7:**
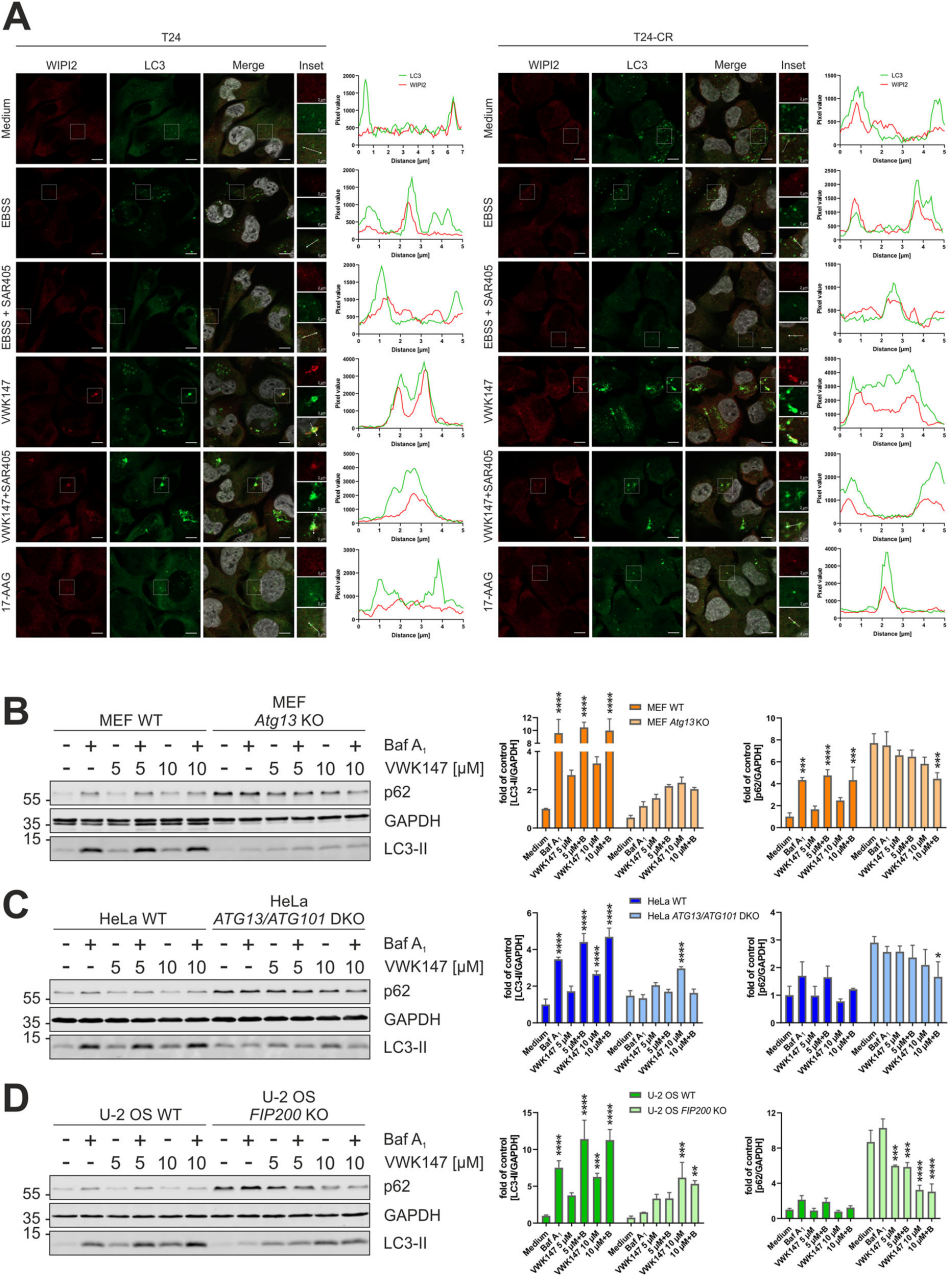
VWK147 induces non-canonical autophagy. **A** T24 and T24-CR urothelial carcinoma cells were grown on glass coverslips 1 or 2 days prior to treatment. Cells were treated with EBSS or 5 µM VWK147 alone or in combination with 5 µM SAR405, or 5 µM 17-AAG. Imaging was performed using a Zeiss Axio Observer 7 fluorescence microscope equipped with a 40x/1.4 Oil DIC M27 Plan-Apochromat objective and ApoTome 2. Representative sections are depicted. Scale bars: 10 µm and 2 µm. The line graphs represent the pixel intensities of the areas indicated by the respective dashed white arrows shown in the insets. See also Fig. S5. **B** MEF WT and MEF *Atg13* KO, **C** HeLa WT and HeLa *ATG13/ATG101* DKO and **D** U-2 OS WT and U-2 OS *FIP200* KO were treated with indicated concentrations of VWK147 in absence or presence of 20 nM bafilomycin A_1_ for 6 h. After treatment, the cells were lysed, and cellular lysates were immunoblotted for p62, LC3 and GAPDH. One representative immunoblot is shown. The quantifications of indicated ratios are from three independent experiments (means + SD). P values were determined by ordinary two-way ANOVA with Tukey´s multiple comparisons test (comparison to the solvent control of the respective cell line). *p ≤ 0.05; **p ≤ 0.01; ***p ≤ 0.001; ****p ≤ 0.0001.

Since we observed LC3-II accumulation upon VWK147 treatment but rather unaffected p62 levels, we next analyzed if VWK147 induces conjugation of ATG8 proteins to single membranes (CASM). This non-canonical autophagy pathway is independent of the autophagy-inducing ULK1 and PIK3C3 complexes [18]. For this, we made use of the PIK3C3 inhibitor SAR405 [38] as control, since it blocks canonical LC3 lipidation. The efficacy of this compound was confirmed in cisplatin-sensitive and –resistant T24, in which it abolished EBSS-induced WIPI2 and LC3 puncta formation (Fig. 7A). WIPI2 binds to phosphatidylinositol 3-phosphate, the catalytic product of the PIK3C3 complex, and mediates the recruitment of the ATG8 protein lipidation machinery during canonical autophagy [39]. Of note, VWK147-mediated formation of LC3-positive aggregates/clusters was not affected by SAR405 (Fig. 7A and Fig. S10), pointing towards PIK3C3-independent LC3 lipidation. Although WIPI2 is not required for CASM, we observed that these VWK147-induced LC3-positive structures were frequently also positive for WIPI2, and that especially these double-positive structures were insensitive to SAR405 in both T24 sublines. In order to test for a possible CASM induction by an independent approach, we monitored LC3 lipidation by immunoblotting in different cell lines deficient for different components of the autophagy-inducing ULK1 complex, i.e., *Atg13* knockout murine embryonic fibroblasts, *ATG13/ATG101* double knockout HeLa, and *FIP200* knockout U-2 OS. First, we confirmed the corresponding KOs and an abolished starvation-induced autophagic flux in these cells (Fig. S11). VWK147 induced LC3 lipidation in the corresponding wild-type cell lines and also in all tested KO cell lines (Fig. 7B–D). Lipidated LC3 levels remained unaffected or were rather reduced by BafA_1_ co-treatment (Fig. 7B–D). Furthermore, p62 accumulation was reduced upon VWK147 treatment in a dose-dependent manner in the KO cell lines. In contrast, the N-terminal inhibitor 17-AAG did not evoke a substantial LC3 lipidation or a reduced p62 accumulation in the KO cell lines (Fig. S12). Finally, we assessed whether CASM induction might represent a general response to CTD-dependent inhibition of HSP90. For that, we employed novobiocin [40] and confirmed reduced expression of the client proteins AKT and ULK1 (Fig. S13A). Similar to VWK147, novobiocin induced LC3 lipidation and reduced p62 levels in our UCC models (Fig. S13B, C) and in *FIP200* KO U-2 OS (Fig. S13D). LC3 lipidation was again partially reduced by concomitant BafA_1_ treatment, suggesting that a BafA_1_-sensitive non-canonical LC3 lipidation might represent a general phenomenon of CTD-dependent HSP90 inhibition. However, it needs to be mentioned that novobiocin concentrations were 200–500-fold higher than VWK147 concentrations.

## Discussion

**Fig. 8:**
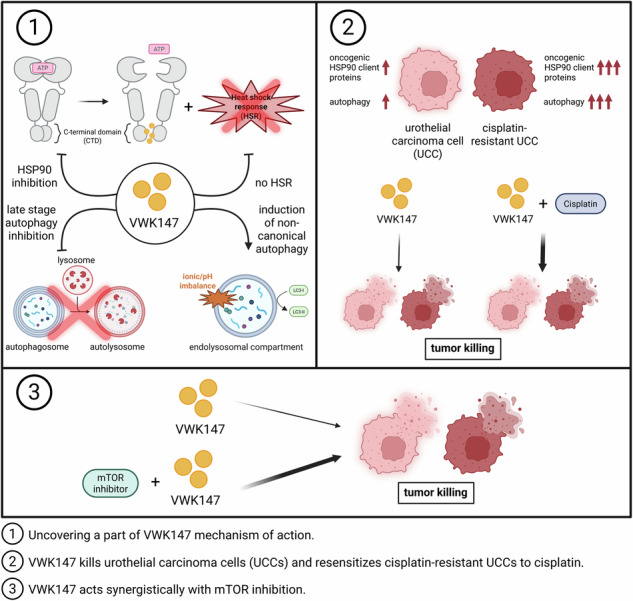
Graphical summary of VWK147’s cellular effects. VWK147 inhibits HSP90 without inducing a heat shock response. In addition, VWK147 inhibits canonical autophagy and concomitantly induces CASM-like processes (panel 1). As mono-treatment, VWK147 kills urothelial carcinoma cells and cisplatin-resistant derivatives thereof. Furthermore, VWK147 increases the efficacy of cisplatin in sensitive UCCs and re-sensitizes cisplatin-resistant UCCs (panel 2). Finally, we observed that VWK147 acts synergistically with mTOR inhibition (panel 3). Figure was created with BioRender.com.

Targeting HSP90 has become a promising approach in anticancer therapy. Several cancer-associated proteins are regulated by HSP90, and various HSP90 inhibitors have been assessed in clinical trials [2]. Frequently, therapeutic interventions are antagonized by cellular stress responses. In the case of HSP90 inhibition, its efficacy is hampered by the induction of HSR. Additionally, autophagy is also employed by cancer cells in order to adapt to metabolic stress and/or chemotherapy. Here, we reported the synthesis, molecular modeling, biochemical characterization, and cell biological assessment of a small molecule inhibitor of C‑terminal HSP90 dimerization that additionally interferes with two central survival pathways in cancer cells, i.e., this compound does not induce HSR and blocks canonical autophagy. We observed that this compound re-sensitizes cisplatin-resistant cancer cells to cisplatin and acts synergistically with mTOR inhibition (Fig. 8).

In recent years, the crosstalk between HSP90 and autophagy has been investigated, and several autophagy-regulating proteins have been identified as HSP90 client proteins [41]. HSP90 has been reported to regulate Toll-like receptor (TLR)-induced autophagy by interacting with Beclin 1, which is a core component of the autophagy-inducing VPS34/PIK3C3 complex [42]. Similarly, ULK1-mediated mitophagy is regulated by the HSP90-Cdc37 chaperone complex [28]. Also chaperone-mediated autophagy (CMA) is regulated by HSP90, as lysosome-associated HSP90 ensures the stability of LAMP-2A at the lysosomal membrane [43]. Accordingly, it is not surprising that HSP90 inhibition affects autophagy signaling. Of note, both autophagy-inducing and -inhibiting properties of HSP90 inhibitors have been reported. Generally, HSP90 inhibition-mediated induction of autophagy might be explained by the inactivation of client proteins such as AKT or mTOR. At the same time, these pro-autophagic effects might be counteracted by the inactivation of pro-autophagic client proteins such as ULK1. Additionally, different cellular model systems and/or differential effects on starvation-induced vs. basal autophagy have to be considered when analyzing autophagy modulation by HSP90 inhibition. Joo et al. reported that 17-AAG inhibits starvation-induced autophagy [28]. Impaired autophagic flux was also observed in A549 lung cancer cells exposed to coumarin pyrazoline compounds that might represent HSP90 inhibitors [44]. Mechanistically, it has also been suggested that HSP90 inhibition diminishes the expression of ATG7, thereby impeding autophagic responses [45]. In our case, this effect does not appear to play a major role, since we observe strong LC3 lipidation upon VWK147 mono-treatment. In contrast to the anti-autophagic effects listed above, several reports describe the induction of autophagy upon HSP90 inhibition [46–49]. Schaefer et al. observed that co-treatment consisting of 17-AAG and the kinase inhibitor D11 induces autophagy [47]. D11 causes the suppression of the heat shock response, and thus 17-AAG/D11 co-treatment might be mimicked by our single treatment with VWK147. However, our analyses using the RFP-EGFP-rLC3 reporter construct indicate an inhibition of autophagy upon VWK147 treatment, which might contribute to its cytotoxicity in our UCC models. Accordingly, HSP90 inhibition and inhibition of late-stage autophagy are molecular properties that are combined in VWK147 and that might be central for its therapeutic efficacy.

Non-canonical LC3 lipidation represents an autophagy-related signaling pathway that gained recent interest [18]. The conjugation of ATG8 proteins to single membranes (CASM) occurs independently of ULK1- and VPS34/PIK3C3-complexes, and this is also the case for VWK147-induced LC3 lipidation, as shown by the usage of the VPS34/PIK3C3 inhibitor SAR405 or different cell lines deficient for different components of the ULK1 complex. Interestingly, we also observe VPS34/PIK3C3-dependent phenomena upon VWK147 treatment, such as formation of WIPI2-positive aggregates/clusters. It has been reported that starvation-induced WIPI2 puncta formation is inhibited by Wortmannin, but that the association of WIPI2 with membranes remains unaffected [50]. The VWK147-induced LC3-positive aggregates/clusters are clearly different from starvation-induced puncta and frequently reveal a vesicular or tube-like morphology. These structures might be membranous, and thus WIPI2 association is possible. Generally, it appears that VWK147 blocks canonical autophagy in cells that are autophagy-competent (i.e., harboring the autophagy-mediating machinery), whereas VWK147-mediated non-canonical LC3 lipidation is clearly detectable in cells lacking the components of the ULK1 complex. Accordingly, the LC3/WIPI2-positive clusters observed in T24 cells may also represent stalled autophagosomes and not necessarily CASM-related structures. It should also be noted that CASM is generally dependent on the vacuolar H^+^-ATPase and thus blocked by BafA_1_. Partially we observe this dependency, but sometimes VWK147-induced LC3-II levels remain unaffected by BafA_1_ also in cells lacking the components of the ULK1 complex. Interestingly, both VWK147 and novobiocin reduced p62 levels in wild-type cells and in cells deficient for components of the ULK1 complex, and especially in the latter case, this reduced p62 accumulation was not sensitive to BafA_1_ treatment. Taken together, the relative contributions of (1) the inhibition of canonical autophagy and (2) the induction of non-canonical LC3 lipidation need to be characterized in future studies. Generally, a VWK147-dependent effect on the endolysosomal compartment might also be deduced from our flow-cytometric observations in the FITC-Annexin V/PI staining, as it has been previously suggested that lysosomal damage can result in both apoptotic and necrotic cell death.

We also observe a clear difference between 17-AAG and VWK147 on ULK1 levels. It has previously been described that 17-AAG treatment results in a faster-migrating, presumably hypophosphorylated form of ULK1 [28]. In contrast, VWK147 treatment does not alter the migrational behavior of ULK1 but reduces its overall levels. These differential observations might point towards differential effects of these two inhibitors on the “client spectrum” of upstream kinases phosphorylating ULK1. Similarly, we do not observe non-canonical LC3 lipidation for 17-AAG, whereas this is the case for the CTD-targeting inhibitors VWK147 and novobiocin. Collectively, differential effects of NTD- and CTD-targeting HSP90 inhibitors await further clarification.

We did not observe major differences between cisplatin-sensitive and -resistant cells with regard to VWK147 mono-treatment (with the exception of a slightly earlier onset of caspase-3 activation in the cisplatin-resistant T24 subline). We have previously shown that autophagy-regulating proteins are up-regulated in cisplatin-resistant UCCs [37], possibly indicating an autophagy-addicted state of these cells. However, this “autophagy-addiction” does not appear to render them more sensitive towards VWK147 mono-treatment. This might be explained by the fact that VWK147 exerts multiple additional cellular effects, i.e., inhibition of HSP90 and/or non-canonical LC3 lipidation. Generally, synergism between cisplatin and HSP90 inhibition has been reported in several cancers [51–56], and also in bladder cancer [57]. Along these lines, we observed increased caspase-3 activation and PARP1 cleavage in both UCC pairs upon cisplatin + VWK147 treatment compared to cisplatin alone. In other words, VWK147 might increase efficacy of cisplatin in sensitive UCCs and re-sensitize cisplatin-resistant UCCs. Söti et al. reported that cisplatin represents a selective nucleotide competitor of the C-terminal ATP-binding site in HSP90 [58]. Accordingly, simultaneously targeting HSP90 CTD dimerization with VWK147 and nucleotide binding with cisplatin may effectively abolish HSP90 CTD activity. Since HSP90 CTD activity is critical for stabilizing numerous client proteins, this dual inhibition strategy suggests that VWK147 could serve as an effective combination partner with cisplatin (Fig. 8).

The PI3K/AKT/mTOR pathway is an essential pathway that regulates various cellular processes, including cell growth, proliferation, survival, and autophagy [59, 60]. In several cancers, the mTOR signaling pathway is dysregulated [61]. Accordingly, mTOR has emerged as an attractive therapeutic target, and both allosteric (rapamycin and analogs, i.e., rapalogs) and ATP-competitive kinase inhibitors (TORKinibs) have been or are currently tested in clinical trials [31]. The efficacy of monotherapies targeting mTOR is hampered by the induction of feedback survival loops [31], resulting in the development of combinatorial approaches. There exists a crosstalk between mTOR and HSP90 signaling. On the one hand, mTOR inhibition can help to overcome HSP90-induced heat-shock responses via the suppression of HSF1 and HSP70 expression [32]. On the other hand, HSP70 associates with Rictor and is required for mTORC2 formation and activity [62]. In recent years, the combination of HSP90 and mTOR inhibitors has been shown to be an effective therapeutic approach in various cancer models [26, 63–67]. Recently, Pan et al. reported the synthesis and evaluation of a dual HSP90/mTOR inhibitor [26]. The authors describe an in vivo anti-tumor activity of their compound in UCC xenograft models. However, efficacy in cisplatin-resistant cells was not assessed and awaits further analysis. Kim et al. reported that the N-terminal HSP90 inhibitor 17-DMAG and the PI3K/mTOR inhibitor NVP-BEZ235 show a synergistic anti-tumor effect in cisplatin-resistant T24 cells [67]. We do not observe synergism between the N-terminal inhibitor 17-AAG and Torin2, but this may be due to the different nature of the applied inhibitors and/or different cellular model systems. However, we do observe synergism between VWK147 and mTOR inhibition by either the TORKinib Torin2 or the rapalog Temsirolimus in UCC lines (Fig. 8). This effect was clearly observed in UCC cell lines and to a lesser extent in HBLAK cells, which rather represent a preneoplastic stage. This might indicate that there is a therapeutic window dependent on the neoplastic stage. However, HBLAK cells are also immortalized and cannot be completely considered as a replacement model for primary uroepithelial cells. We propose that the synergy observed in UCCs is caused by two VWK147-dependent effects on cellular survival responses: (1) lacking induction of heat-shock response, and (2) inhibition of autophagy. As mentioned above, we have previously reported that autophagy-related proteins are up-regulated in different cisplatin-resistant UCCs compared to the sensitive parental cell lines [37], and one could speculate that these cells may be particularly susceptible to treatment with autophagy-modulating compounds. As discussed above, we did not observe this for VWK147 mono-treatment. However, if these cells are simultaneously exposed to autophagy-activating conditions (i.e., mTOR inhibition), an effect may become apparent. The induction of autophagy and the simultaneous blockade of this process have been proposed as an anti-cancer strategy [68–70], and this might exactly be the mechanism we observe here: the induction of early autophagy events by mTOR inhibition, and the blockade of late-stage autophagy by VWK147. Certainly, the relevance and/or contribution of the described non-canonical LC3 lipidation awaits further clarification. Nevertheless, VWK147 represents a promising inhibitor of the C-terminal HSP90 dimerization with double-edged autophagy-modulating properties, and this compound can—as monotherapy or in combination with cisplatin or mTOR inhibitors—prove useful in order to optimize anti-cancer therapies and/or to overcome therapy resistance.

## Materials and methods

### Procedures, synthetic methods, and analytical data for the synthesis of VWK147 (2)

#### NMR spectroscopy

Nuclear magnetic resonance (NMR) spectra were recorded on Bruker Avance™ III-300 (300 MHz for ^1^H, 75 MHz for ^13^C) spectrometer. All ^13^C spectra reported here were acquired with broadband proton decoupling. Chemical shifts (δ) are given in parts per million (ppm) relative to the external standard tetramethylsilane (TMS). NMR spectra were recorded in CDCl_3_, (CD_3_)_2_CO or CD_3_OD. Spectra were referenced to residual solvent signals.

#### HPLC method

A Knauer HPLC system equipped with an Azura P 6.1 L pump, Optimas autosampler, and K-2600 photometer was used. Reversed-phase chromatography was performed on a Knauer 150×4 mm Eurospher II 100-5 C 18 column (serial number: FK36). Samples were prepared by dissolving 1 mg of analyte in 1 ml of acetonitrile or DMSO. The mobile phases consisted of water with 0.1% trifluoroacetic acid (TFA) (mobile phase A) and acetonitrile with 0.1% TFA (mobile phase B). The gradient elution program was as follows:

**Table:** 

Time (min)	Mobile Phase A (%)	Mobile Phase B (%)
0–0.5	90	10
0.5–20	90 → 0	10 → 100
20–30	0	100

The flow rate was 1 ml/min. Detection was at 254 nm. The run time was 30 min per sample. All compounds showed purities greater than 95% by HPLC analysis.

#### Melting point determination

Melting points were recorded on a Büchi Melting Point B-545 apparatus and are uncorrected.

#### Mass spectrometry

High-resolution electrospray ionization mass spectra (ESI-HRMS) were recorded on a Bruker Daltonics maXis 4 G UHR-QTOF mass spectrometer.

#### Procedure for the synthesis of tripyrimidonamide **2** (VWK147)

Tripyrimidonamide **1** (1.05 g, 1.35 mmol) was dissolved in 6 ml of a 0.5 M solution of NaOH in methanol, and the reaction mixture was stirred at 80 °C for 5 h. The solvent was removed under reduced pressure, affording a yellow solid. The residue was purified by flash column chromatography (n-hexane/ethyl acetate, ratio) to give the main product fractions, which were combined and concentrated under reduced pressure. The resulting bright yellow solid was recrystallized from n-hexane/ethyl acetate to yield compound **2** as a yellow solid in 40% yield.

#### Analytical data for tripyrimidonamide **2** (VWK147)

**Yield**: 40%; 362 mg (0.54 mmol), yellow solid

**Mp**: 226 °C (dichloromethane)

**HPLC**: R_t_: 15.567 min, purity: 97.2%

^**1**^**H NMR** (300 MHz, chloroform-*d*) δ 10.44 (s, 1H), 10.11 (s, 1H), 8.96 (s, 1H), 8.93 (s, 1H), 7.84–7.65 (m, 1H), 7.38 (s, 1H), 7.31 (d, *J* = 8.2 Hz, 2H), 6.81 (d, *J* = 8.2 Hz, 2H), 5.98 (s, 2H), 5.31–5.11 (m, 2H), 4.66 (d, *J* = 6.7 Hz, 2H), 3.75 (s, 3H), 2.99 (s, 3H), 2.36–2.20 (m, 1H), 2.18–1.90 (m, 2H), 1.68 (d, *J* = 6.0 Hz, 3H), 0.93 (d, *J* = 6.2 Hz, 6H), 0.84 (t, *J* = 7.1 Hz, 3H).

^**13**^**C NMR** (75 MHz, chloroform-*d*) δ 160.92, 158.99, 158.89, 158.73, 157.94, 157.88, 144.75, 144.31, 136.83, 136.52, 134.20, 132.99, 129.64, 129.01, 128.29, 126.98, 123.50, 113.85, 59.98, 55.23, 51.03, 46.56, 28.81, 26.65, 25.88, 19.82, 17.87, 11.36.

**HR-MS (ESI**+**):** calculated for [C_32_H_38_N_10_O_7_ + H^+^] m/z: 675.2998; found: 675,2995

#### Thermal shift assay

The thermal shift assay was performed as previously described [8]. CTD r-HSP90α (5 µM) protein and VWK147 (100 µM) were mixed together in the assay buffer (1× PBS, pH = 7.5) and were incubated for 2 h. Then, 6× SYPRO orange dye (Sigma-Aldrich) was added to the mixture to a final volume of 20 μl. 96-well polymerase chain reaction (PCR) plates and a PCR system (BioRad, CFX Connect real-time system) were used to heat the samples from room temperature to 95 °C in increments of 0.5 °C for 10 s, with the excitation wavelength at 470 nm and emission wavelength at 570 nm. For a determination of protein melting temperature values (Tm), the melting curve for each data set was analyzed by GraphPad Prism 8.0.2 and fitted with the sigmoidal Boltzmann fit. Melting temperatures without the inhibitors (DMSO) were used as a control.

#### Cellular thermal shift assay (CETSA)

A CETSA assay was performed as described previously [8]. K562 cells were incubated with VWK147 (or DMSO) for 24 h. Cells were harvested by centrifugation (400 × *g* for 5 min at room temperature) and washed five times with ice-cold PBS. The pellets were dissolved in PBS and later equally divided into 200 μl PCR tubes. Solutions were heated at the indicated temperature gradient for 3 min and 30 s (T-Gradient Cycler, Biometra). Aliquots were then snap-frozen in liquid nitrogen and thawed at 25 °C in a thermal cycler (GeneAMP PCR System2700, Applied Biosystems) three times, followed by centrifugation at 10,000 × *g* for 20 min at 4 °C. The supernatants were harvested, and protein levels were measured by a quantitative simple western immunoassay (JESS, BioTechne, Minneapolis, MN). JESS was performed following the manufacturer’s instructions. Protein levels represented by the area under the curve of the electropherograms were normalized to the lowest temperature set as 0% degradation. The ΔTm value was determined by plotting normalized data using a sigmoid dose curve and nonlinear regression (GraphPad Prism 8.0.2).

#### Time-resolved fluorescence resonance energy transfer (TR-FRET) assay

An evaluation of the C-terminal HSP90 binding affinity to PPID (cyclophilin D) was performed using the HSP90 CTD TR-FRET assay kit (50289, BPS Bioscience, San Diego, CA). For the positive control, the inhibitor was substituted for DMSO, and for the negative control, PPID-GST-tag was substituted for 1x HSP90 assay buffer. The assay was performed as described previously [8]. Samples were incubated for 2 h at room temperature protected from light and measured with a microplate-reader (SPARK10M, Tecan). Fluorescence was measured using a time-resolved reading mode with two subsequent measurements: The first measurement was performed using a 340 nm/620 nm (excitation/emission) wavelength with a lag time of 60 μs and integration time of 500 μs. The second measurement was performed using a 340 nm/665 nm (excitation/emission) wavelength with a lag time of 60 μs and integration time of 500 μs. Data analysis was performed using the TR-FRET ratio (665 nm emission/620 nm emission). The TR-FRET ratios are normalized to % activity by setting the negative control as 0% activity and the positive control as 100% activity [(FRET_sample_ – FRET_neg_)/(FRET_pos_ – FRET_neg_) × 100%]. Two-way ANOVA was performed to calculate significance against DMSO control.

#### Fluorescence polarization (FP) assay

An evaluation of the binding affinity of compounds toward the ATP pocket of HSP90 NTD was determined by a competitive binding assay against FITC-labeled geldanamycin (GM) using the HSP90 NTD assay kit (50293, BPS Bioscience) as described previously [8]. The inhibitor sample wells were filled with 1X HSP90 assay buffer, supplemented with DTT, BSA, FITC-labeled GM (100 nM), and 10 μl of VWK147 (final concentration 10 µM). The reaction was initiated by adding 20 μl of Hsp90 (17 ng/μl) and incubating at room temperature for 3 h with slow shaking. Background wells (master mix only), negative controls (FITC-labeled GM, buffer, and DMSO), and positive controls (FITC-labeled GM, buffer, DMSO, and Hsp90) were also included within the assay plate. Fluorescence polarization was measured at a 470 nm excitation wavelength and 525 nm emission wavelength in a microtiter-plate reader (Infinite M1000pro by Tecan). The instrument-specific g-factor of 1.187 was used to calculate the polarization ((I_II_ – G(I┴)/(I_II_ + G(I┴)) × 1000, whereas the percentage of HSP90-bound FITC-GM was calculated using P_norm_ = (P_Inhibitor_ − P_neg_)/(P_pos_ – P_neg_)*100. Statistical analysis was performed utilizing two-way ANOVA against unlabeled Geldanamycin.

#### Luciferase refolding assay

A luciferase refolding assay was performed as previously described [8]. Recombinant firefly luciferase from Photinus pyralis (Sigma-Aldrich, St. Louis, MO; 10 × 1010 units/mg) was diluted (1:100) in denaturation buffer (25 mM Tricine, pH 7.8, 8 mM MgSO4, 0.1 mM EDTA, 1% Triton X-100, 10% glycerol, and 10 mg/ml BSA) at 38 °C for 8 min. Rabbit reticulocyte lysate (Promega, Madison, WI) was diluted 1:1 by the addition of cold mix buffer (100 mM Tris, pH 7.7, 75 mM Mg(OAc)2, 375 mM KCl, and 15 mM ATP), creatine phosphate (10 mM), and creatine phosphokinase (16 U/ml) and was preincubated at 30 °C with VWK147 and controls (DMSO, Tanespimycin, PU-H71, and AUY922) for 1 h. Afterward, 1 μl of denatured luciferase or active luciferase (as a control) was added to 20 μl of a rabbit reticulocyte mixture. As a control, denatured or active luciferase was incubated without reticulocyte lysate in buffer containing 20 mM Tris, pH 7.5, 150 mM NaCl, 1% hemoglobin, and 4% BSA. At desired time points, 1.5 μl samples were removed and added to 40 μl of assay buffer (25 mM Tricine, pH 7.8, 8 mM MgSO4, 0.1 mM EDTA, 33 μM DTT, 0.5 mM ATP, and 0.5 mM luciferin), and the luminescence was read using a Spark microplate reader (Tecan). Percent luciferase refolding was determined by comparing the samples to DMSO (100%).

#### BS3 crosslinker assay

Hsp90 CTD dimerization was evaluated using an amine-reactive chemical cross-linker bis(sulfosuccinimidyl) suberate (BS3) (Pierce) as previously described (DOI: 10.1021/acscentsci.2c00013). HSP90α CTD protein (2 μM) was diluted in Na_2_HPO_4_ (25 mM; pH 7.4) and treated with different concentrations of the inhibitor VWK147 to make a final volume of 25 μl. The reaction mixture was incubated at room temperature for 1 h. The amine-reactive cross-linker BS3 was added to a final concentration of 100 μM, and the samples were incubated for 1 h at room temperature. Cross-linking was quenched by the addition of SDS sample buffer and subsequent heating for 5 min at 95 °C. Samples were run in 12% SDS-PAGE gels followed by Western blotting. Blots were probed with anti-HSP90 (AC88, Abcam) antibody.

### Reagents

Torin2 (#S2817), Temsirolimus (#S1044) and SAR405 (#S7682) were purchased from Selleck Chemicals (Houston, TX, USA); bafilomycin A_1_ (#B1793) was purchased from Sigma-Aldrich (St. Louis, MO, USA). Additionally, the following reagents were used: dimethyl sulfoxide (DMSO; AppliChem GmbH, #A3672), staurosporine (STS; [Biozol, #S-9300]), Q-VD-Oph (QVD; [Selleck Chemicals, #S7311]), milk powder (Carl Roth, #T145.2), PBS (Thermo Fisher Scientific/Gibco, #14190-094), 0.05% trypsin/EDTA solution (Thermo Fisher Scientific/Gibco, #25300-062), hygromycin (InvivoGen, #ant-hg-1), puromycin (InvivoGen, #ant-pr-1) and. For immunoblotting, primary antibodies against SQSTM1/p62 (PROGEN Biotechnik, #GP62-C), LC3 (Cell Signaling Technology, #2775), GAPDH (abcam, #8245), β-Actin (Sigma-Aldrich, #A5316), phospho-mTOR Ser2448 (Cell Signaling Technology, #2971), mTOR (CST, #2972), phospho-ULK1 Ser757 (CST, #6888), ULK1 (clone D8H5, CST, #8054), ATG14 (MBL, #PD026; CST, #5504), phospho-ATG14 Ser29 (CST, #92340), p70S6K (CST, #2708), phospho-p70S6K Thr389 (CST, #9234), S6RP (CST, #2217), phospho-S6RP Ser240/244 (CST, #2215), Vinculin (Sigma-Aldrich, #V9131), tubulin (Sigma, #T5168), poly (ADP-ribose) polymerase (PARP) 1 (Enzo, #MBL-SA250; CST, #9542), Caspase-3 (CST, #9662), RIPK1 (BD Biosciences, #610459), CDK4 (Thermo Fisher Scientific, #MA5-13720, or CST, #12790), HSP90 (CST, #4875), HSP70 (Abcam, #ab2787), HSP40 (CST, #4871), HSP27 (CST, #2402), phospho-AKT Ser473 (CST, #9271), AKT (CST, #9272), FLAG (Sigma-Aldrich, #F1804), FIP200 (Proteintech, #17250-1-AP), ATG13 (Sigma-Aldrich, #SAB4200100) and ATG101 (CST, #13492) were used. IRDye 680- or IRDye 800-conjugated secondary antibodies (#926-68077, #926–68072/73, #926-32212/13) were purchased from LI-COR Biosciences.

For immunofluorescence, primary antibodies against WIPI2 (Biorad, #MCA5780GA) and LC3B (MBL, #PM036) were used. Alexa Fluor^®^ 488-conjugated and Alexa Fluor^®^ 647-conjugated secondary antibodies were purchased from Jackson ImmunoResearch Laboratories (#115-545-003 and #111-605-003) and from Thermo Fisher Scientific (#A32728, #A32731 and #A31573). DAPI was obtained from Roth (#6335.1).

### Cell lines and cell culture

All urothelial carcinoma cell lines (UCCs) used in this research were kindly provided by Margaretha Skowron, Department of Urology, Heinrich Heine University Düsseldorf, Düsseldorf, Germany. K562 cells were obtained from the German Collection of Microorganisms and Cell Cultures GmbH (DMSZ, #ACC 10). Plat-E cells were kindly provided by Toshio Kitamura, Institute of Medical Science, University of Tokyo, Japan. Wild-type and *Atg13* KO MEFs were provided by Noboru Mizushima (University of Tokyo, Japan). Wild-type and *FIP200* KO U-2 OS cells were provided by Fulvio Reggiori (Aarhus University, Denmark). The human uroepithelial cell line HBLAK was purchased from CELLnTEC (#HBLAK). All cells (except for HBLAK) were cultured in DMEM (Thermo Fisher Scientific/Gibco, #41965) or RPMI (Thermo Fisher Scientific/Gibco, #61870) supplemented with 10% FCS (Sigma-Aldrich #F0804, LOT BCCB7649 and #F9665, LOT 0001655439), 4.5 g/l D-glucose, 100 U/ml penicillin and 100 μg/ml streptomycin (Thermo Fisher Scientific/Gibco, #15140-122) at 37 °C and 5% CO_2_ humidified atmosphere. During cell culture, cisplatin was added to the media of UCCs with every passage in concentrations of 4 µg/ml for 253J and 11 µg/ml for T24 cells. For experiments, cisplatin was removed unless specified. HBLAK cells were cultured according to the manufacturer’s recommendation in serum-free CnT-NX-E Epithelial Proliferation Medium (CELLnTEC, #CNT-NX-E) at 37 °C and 5% CO2 humidified atmosphere. During cell culture, cells were detached using Accutase (CELLnTEC, #CnT-Accutase-100).

### Cell viability assays

Cell viability was measured using either Alamar Blue, MTT or CellTiter-Glo Luminescent Cell Viability (CTG) assay (Promega, #G7570). For all assays, cells were seeded in 96-well plates 1 day prior to the experiment (for the CTG Assay, white, clear bottom 96 well plates were used). Wells without cells were included as a blank. The following day, the cells were treated with the indicated compounds as described above for 24–72 h. DMSO was used a solvent control. For the Alamar Blue Assay, 40 µM resazurin sodium salt (Cayman Chemicals, #14322) was added to the cells after treatment and incubated at 37 °C for 2 h. Afterward, the fluorescence intensity (excitation: 560 nm, emission: 590 nm, bandwidth: 4 nm) was measured using a multi-mode, single channel monochromator-based microplate reader equipped with a xenon flash light source and photodiodes as detectors (BioTek, Synergy Mx). For the MTT assay, 0.5 mg/ml MTT (Roth, #4022) was added to the cells and cells were incubated at 37 °C for 1 h. Then the plates were centrifuged at 600 rcf for 5 min, and formazan crystals were dissolved in DMSO for 20 min in dark. The absorbance was measured at test (570 nm) and reference (650 nm) wavelengths using the microplate reader described above. For the CTG Assay, medium was aspirated and replaced with OptiMEM (Thermo Fisher Scientific, #31985062) and equal amounts of CellTiter-Glo reagent. For HBLAK cells, CellTiter-Glo reagent was directly mixed with the medium as the cells tended to detach during the aspiration step. ATP levels were quantified by measuring the luminescence according to the manufacturer´s instructions using a CLARIOstar Plus multi-mode microplate reader (BMG LABTECH). All emitted light was measured wavelength independent in each well by using the scan mode “spiral averaging”, a scan diameter of 6 mm, a gain value of 3600 and a focal height of 14–15 mm after adjustment performances. For analysis, the blank was subtracted from the raw data and data was next normalized to the mean of the DMSO control.

### FITC-Annexin V and PI co-staining

Staining was performed using the FITC Annexin V Detection Kit (BD Biosciences, #556547) and according to the manufacturer’s instructions. Briefly, cells were seeded in a 12-well format 1 day prior to treatment. After treatment, cells were trypsinized, washed twice with cold PBS and then resuspended in 200 µl of 1X Binding Buffer. Cell suspensions were then transferred to FACS tubes and 7.5 µl FITC-Annexin V and 10 µl propidium iodide (PI) were added. Cells were gently vortexed and incubated for 15 min at RT in the dark. Then, 150 µl 1X Binding Buffer was added and cells were analyzed by flow cytometry within 1 h (LSRFortessa, BD Biosciences). Compensation was performed beforehand with staurosporine treated and untreated cells. Both FITC and PI fluorochromes were excited with the 488-nm blue laser. Following filter configurations were used: for the FITC dye, the 530/30 Band Pass filter and 505 Long Pass filter were used. For the PI dye, 695/40 Band Pass filter and 685 Long Pass filter were used. Measurements were performed with the software FACSDiva (version 6.2). The dot plots were generated with the software FlowJo (version 10).

### Fluorometric caspase-3 activity assay

T24 and 253 J were seeded at a density of 6 × 10^4^ cells/well and T24-CR and 253J-CR at a density of 9.5 × 10^4^ cells/well in a 24-well plate format. Next day, cells were treated with the respective agents for the indicated time. Briefly, cells were harvested by centrifugation at 600 × *g* and lysed with 50 μl of ice-cold lysis buffer (20 mM HEPES, 84 mM KCl, 10 mM, MgCl_2_, 200 μM EDTA, 200 μM EGTA, 0.5% NP40, 1 μg/ml leupeptin, 1 μg/ml pepstatin, 5 μg/ml aprotinin) on ice for 10 min. Cell lysates were transferred to a flat-bottom microplate and mixed with 150 μl of ice-cold reaction buffer (50 mM HEPES, 100 mM NaCl, 10% sucrose, 0.1% CHAPS, 2 mM CaCl_2_, 13.35 mM DTT) containing 70 μM of the profluorescent caspase substrate Ac-DEVD-AMC (Biomol GmbH, #ABD-13402). The kinetics of AMC release were monitored by measuring AMC fluorescence intensity (excitation: 360 nm, emission: 450 nm) at 37 °C in intervals of 2 min over a time course of 120 min, using a multi-mode, single-channel monochromator-based microplate reader with a xenon flash light source and photodiodes as detectors (BioTek, Synergy Mx). The slope of the linear range of the fluorescence curves (Δrfu/min) was considered as corresponding to caspase-3 activity. Therefore, the mean slope of DMSO-treated wells per time point and cell line was set to 100 and all other values were normalized to the corresponding DMSO mean.

### Retroviral transduction

cDNA encoding human HSP70 was amplified from 253J cDNA by PCR using the following primers: fwd: GAAGGACCGAGCTCTTCTCG, rev: AGCAATCTTGGAAAGGCCCC. Subsequently, the HSP70 cDNA harboring 5′ and 3′ overlap to pMSCVhygro sequences was amplified and directly cloned into pMSCVhygro by sequence and ligation-independent cloning (SLIC) [71]. The following primers were used: 3xFLAG-HSP70 fwd: GACGATGACAAGGGATCCGCCAAAGCCGCGGCGATCGGCA, 3xFLAG-HSP70 rev: CCCCTACCCGGTAGAATTCCTAATCTACCTCCTCAATGGTGGGGCC, pMSCVhygro fwd: GATTAGGAATTCTACCGGGTAGGGGAGGCGCTTTTCCCAAGGCA, and pMSCVhygro rev: CGGCTTTGGCGGATCCCTTGTCATCGTCATCCTTGTAATCGATG. Plat-E cells were used as packaging cells, and were transfected with the retroviral expression vectors pMSCVhygro, pMSCVhygro-3xFLAG-HSP70, pMSCVpuro or pMSCVpuro-mRFP-EGFP-rLC3 using FuGENE^®^ 6 (Roche, #11988387001). For the retroviral infection, pVSVG was co-transfected into Plat-E cells. After 48 h, UCCs were incubated with the corresponding retroviral supernatants containing 3 µg/ml Polybrene (Sigma-Aldrich, #H9268-106) for 72 h and subsequently selected in medium containing 200 µg/ml hygromycin or 1–2.5 µg/ml puromycin for about 1 week. After, T24 and T24-CR mRFP-EGFP-rLC3 expressing cells were additionally sorted for intermediate GFP signal at the Core Facility Flow Cytometry, University Clinic of Düsseldorf, in order to obtain a cell population expressing similar amounts of the protein.

### Immunoblotting

Cells were harvested by scraping and lysed in ice-cold lysis buffer (20 mM Tris/HCl, pH 7.5, 150 mM NaCl, 0.5 mM EDTA, 1% [v/v] Triton X-100, 1 mM Na_3_VO_4_, 10 μM Na_2_MoO_4_, 2.5 mM Na_4_P_2_O_7_, 10 mM NaF and protease inhibitor cocktail [Sigma-Aldrich, #P2714]). Alternatively, Na_3_VO_4_, Na_2_MoO_4_, Na_4_P_2_O_7_ and NaF were replaced by PHOSSTOP (Roche, #04906837001). Equal amounts of proteins were subjected to 8–15% SDS-PAGE. Proteins were then transferred to PVDF membranes (Millipore, #IPFL00010) or nitrocellulose membranes (Thermo Fisher Scientific, #88018) and analyzed using the indicated primary antibodies and appropriate IRDye-conjugated secondary antibodies. Protein signals were detected using an Odyssey Infrared Imaging system (LI-COR Biosciences) and quantified using Image Studio lite 4.0 and 5.2 (LI-COR Biosciences).

### Immunofluorescence

6.5–7.5 × 104 cells were plated on glass coverslips 1 or 2 days prior to treatment. After treatment, the cells were fixed at room temperature using 4% paraformaldehyde for 15 min. Subsequently, the cells stably expressing mRFP-EGFP-rLC3 were directly stained with DAPI. The other cells were permeabilized with 50 µg/ml digitonin (Roth, #4005) for 5 min, blocked with 3% BSA (Roth, #8076)-PBS for 30 min, and then incubated with indicated primary antibodies for 2 h and corresponding secondary antibodies for 30 min. Cells were stained with DAPI and then embedded in ProLong Glass Antifade Mountant (Thermo Fisher Scientific, #P36980). Fluorescence imaging was performed using a Zeiss Axio Observer 7 epifluorescence microscope equipped with colibri 7 as light source, a 90 HE LED filter cube, a 40x/1.4 Oil DIC M27 Plan-Apochromat objective (Zeiss, Jena, Germany) and ApoTome 2 (Zeiss, Jena, Germany). Images were taken by using ZEN 2.6 pro. DAPI, Alexa Fluor 488, Alexa Fluor 647, GFP and TurboRFP were excited at 385 nm (5% LED intensity), 493 nm (75% LED intensity), 653 nm (75% LED intensity), 488 nm (20% LED intensity) and 553 nm (20% LED intensity), respectively. The exposure times were as following: DAPI: 10 ms; LC3 (AF488): 70 ms; WIPI2 (AF647): 1 s; GFP: 30 ms; TuRFP: 400 ms. Pixel intensity profiles of areas indicated by dashed white arrows were measured with ZEN lite 2.3 and are depicted in bar graphs.

### Molecular dynamics simulations

To generate a conformational ensemble of the solvent-exposed hHSP90 protomer, a monomer taken from the cryo-EM structure of hHSP90 (PDB-ID 7L7I; [72]) was subjected to all-atom explicit solvent MD simulations. For this, the structure was prepared at a pH of 7.4 in Maestro [73] using the protein preparation wizard and PROPKA [74]. MD simulations were performed with the Amber22 package of molecular simulation codes [75]. The ff19SB force field [76] was used to parameterize the protein, Li-Merz parameters for ions [77], and the OPC water model [78] for the solvent. To generate input structures, a shell of at least 12 Å of OPC water, counter ions, and enough KCl to reach a salt concentration of 150 µM was added using Packmol-Memgen [79]. To cope with long-range interactions, the Particle Mesh Ewald method [80] was used; the SHAKE algorithm [81] was applied to bonds involving hydrogen atoms, and a direct-space, non-bonded cut-off of 8 Å was used.

In the beginning, 17,500 steps of steepest descent and conjugate gradient minimization were performed; during 2500, 10,000, and 5000 steps, positional harmonic restraints with force constants of 25 kcal mol^−1^ Å^-2^, 5 kcal mol^−1^ Å^-2^, and zero, respectively, were applied to the solute atoms. Thereafter, 50 ps of NVT (constant number of particles, volume, and temperature) MD simulations were conducted to heat the system to 100 K, followed by 300 ps of NPT (constant number of particles, pressure, and temperature) MD simulations to adjust the density of the simulation box to a pressure of 1 atm and to heat the system to 300 K. During these steps, a harmonic potential with a force constant of 10 kcal mol^−1^ Å^−2^ was applied to the solute atoms. As the final step in thermalization, 300 ps of NVT-MD simulations were performed, while gradually reducing the restraint forces on the solute atoms to zero within the first 100 ps of this step. Afterward, twelve independent production runs of NVT-MD simulations with 1 µs length each were performed. For this, the starting temperatures of the MD simulations at the beginning of the thermalization were varied by a fraction of a Kelvin. Subsequently, the resulting runs were pooled and structures were extracted every ns. Then, a *k*-means clustering algorithm based on RMSD, as implemented in cpptraj [82], was used to assign the structures to five clusters, and the cluster representative was identified. After checking that all twelve runs were represented in the largest cluster, the representative of this cluster was chosen for subsequent docking experiments.

### Molecular docking

For the molecular docking, VWK147 was drawn and converted into a 3D structure with Maestro. Additional to the cluster representative from the above MD simulations, the X-ray crystal structures of hHSP90 with PDB ID 3Q6M [83] and 7L7I were used in their monomeric form and prepared with Maestro as described above. The ligand was subsequently docked into the binding pocket of the respective proteins using the combination of AutoDock as a docking engine and the DrugScore2018 distance-dependent pair-potentials as an objective function [84–86].

### Statistics

For immunoblotting, the density of each protein band was divided by the average of the density of all bands of this protein. The ratios were normalized to the loading control, and fold changes were calculated by dividing each normalized ratio by the average of the ratios of the solvent control (*n* ≥ 3). For PARP1 and Caspase-3, normalized bands representing the fragment of the protein of interest were additionally normalized to the normalized bands of the full-length protein. The results are shown as mean + standard deviation (SD) in the bar diagrams and in the dose-response curves. P values were either determined by ordinary one-way ANOVA with Dunnett’s post hoc or multiple comparisons test, by ordinary two-way ANOVA with Tukey’s multiple comparisons test or by a two-tailed unpaired t-test.

For cell viability assay, results are shown as mean ± standard deviation in the bar diagrams, and P values were determined by two-way ANOVA with Sidak’s post hoc test. All IC_50_ values were calculated using GraphPad Prism (versions 7.01 and 8.0.2). Compusyn 1.0 [33] was used to perform isobologram analysis for the combination of two compounds. The resulting combination index (CI) values represent the combination effects of two compounds and are interpreted as synergistic (CI ≤1), additive (CI = 1) or antagonistic (CI>1) [33].

## Supplementary information

Original data

Supporting Information

## Data Availability

The datasets generated and/or analyzed during the current study are available at Mendeley Data (10.17632/rn23vbrfhb.1).
