# Supplementary material for: Small-molecule inhibitor of C‑terminal HSP90 dimerization modulates autophagy and functions synergistically with mTOR inhibition to kill cisplatin-resistant cancer cells
Source: Cell Death Dis. 2025 Dec 23;17(1):130. doi: 10.1038/s41419-025-08330-4 (PMC12848304; doi:10.1038/s41419-025-08330-4)

Figure 2B:

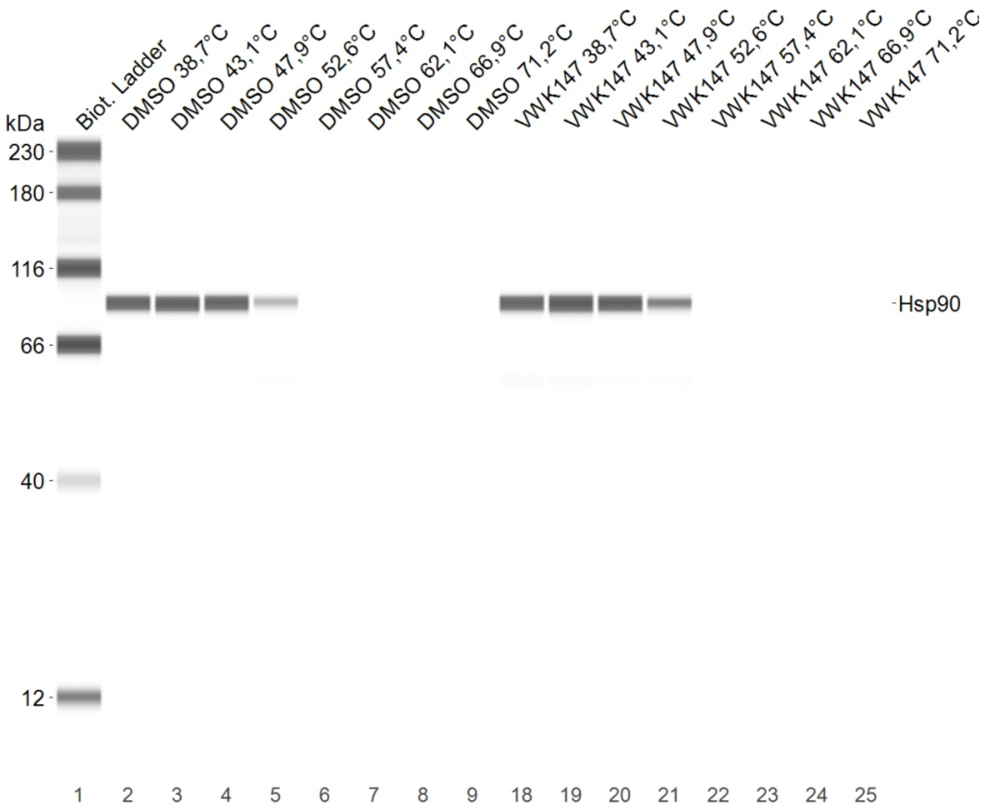

Figure 2F:

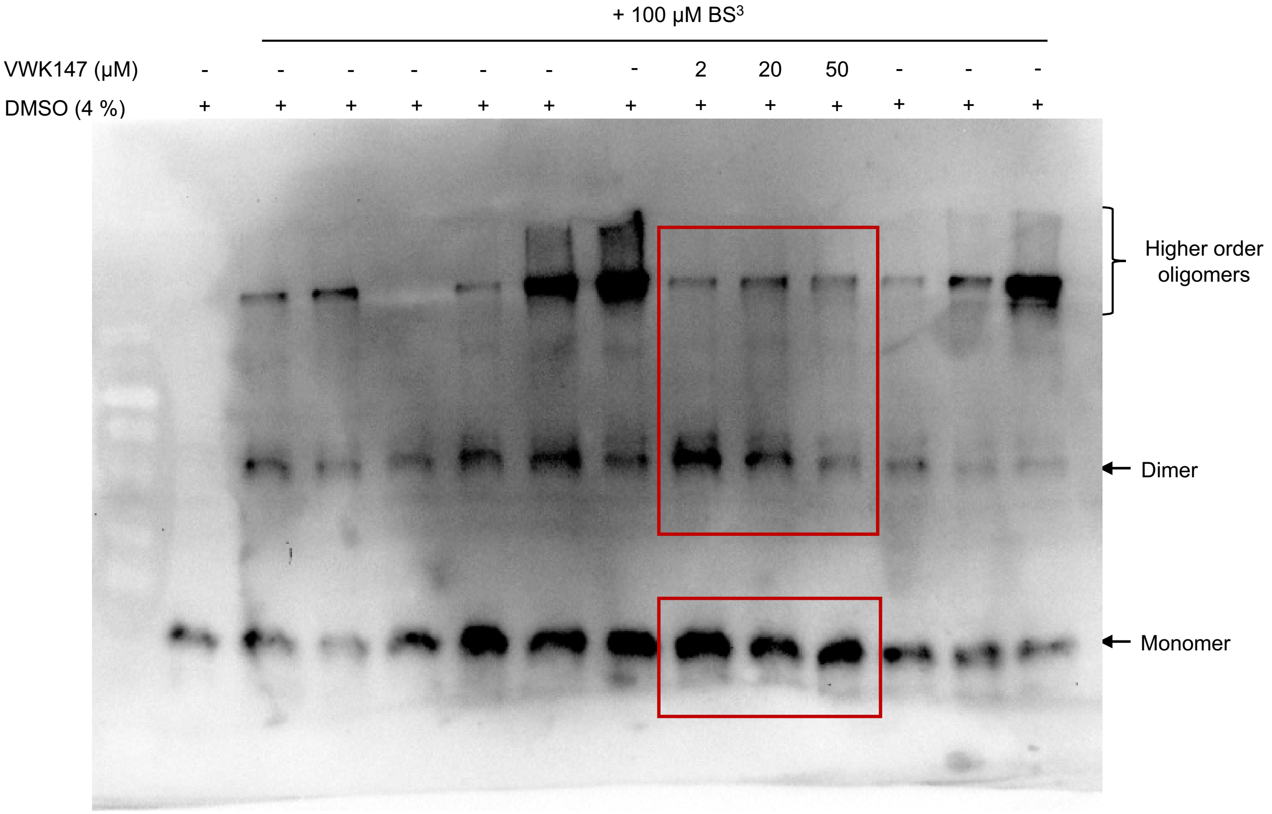

Figure 3A:

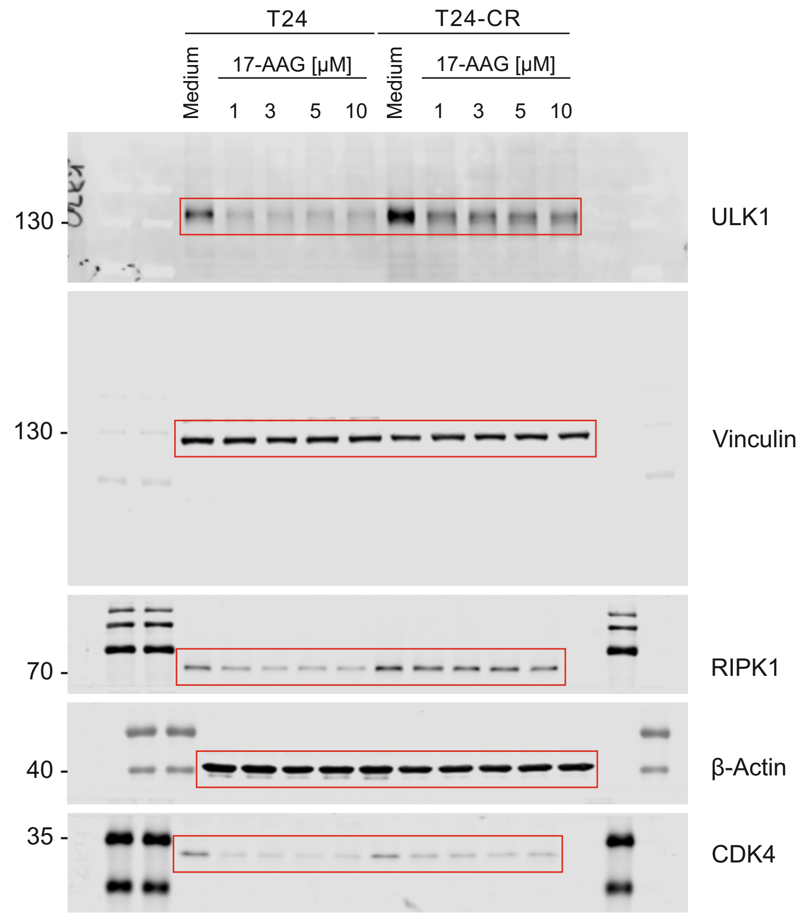

Figure 3B:

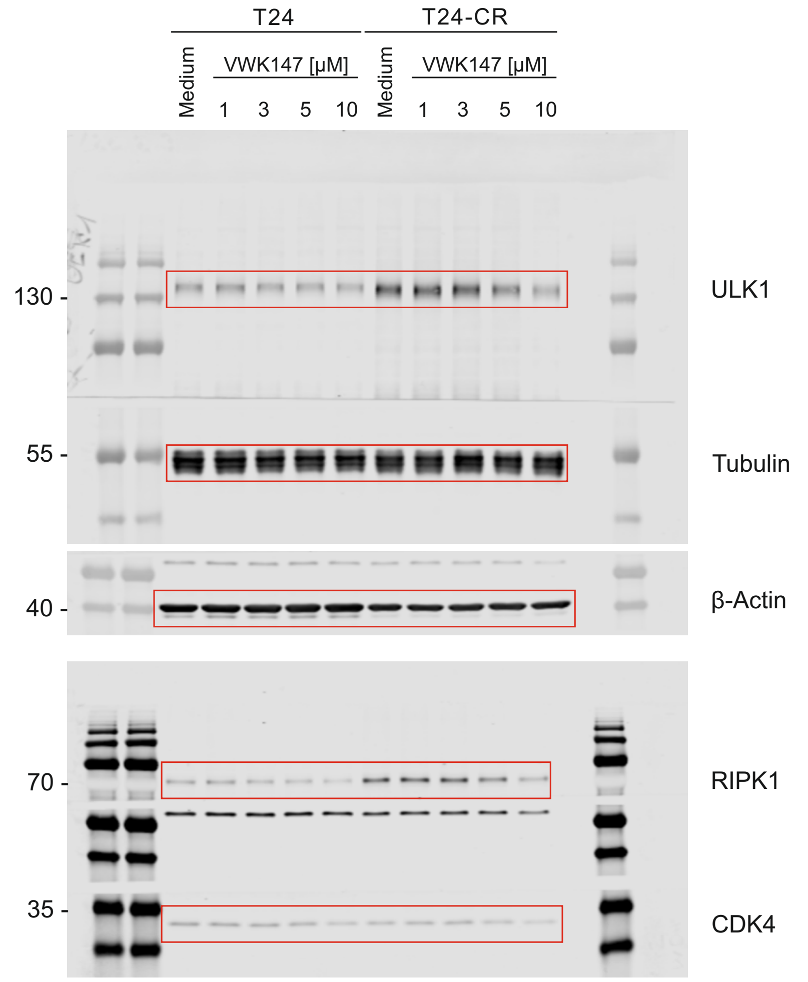

Figure 3C:

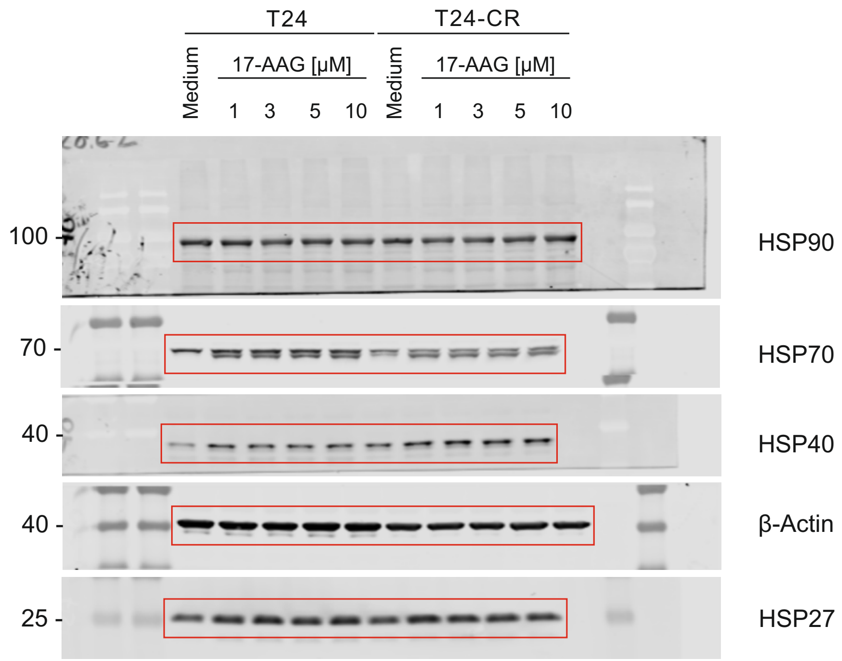

Figure 3D:

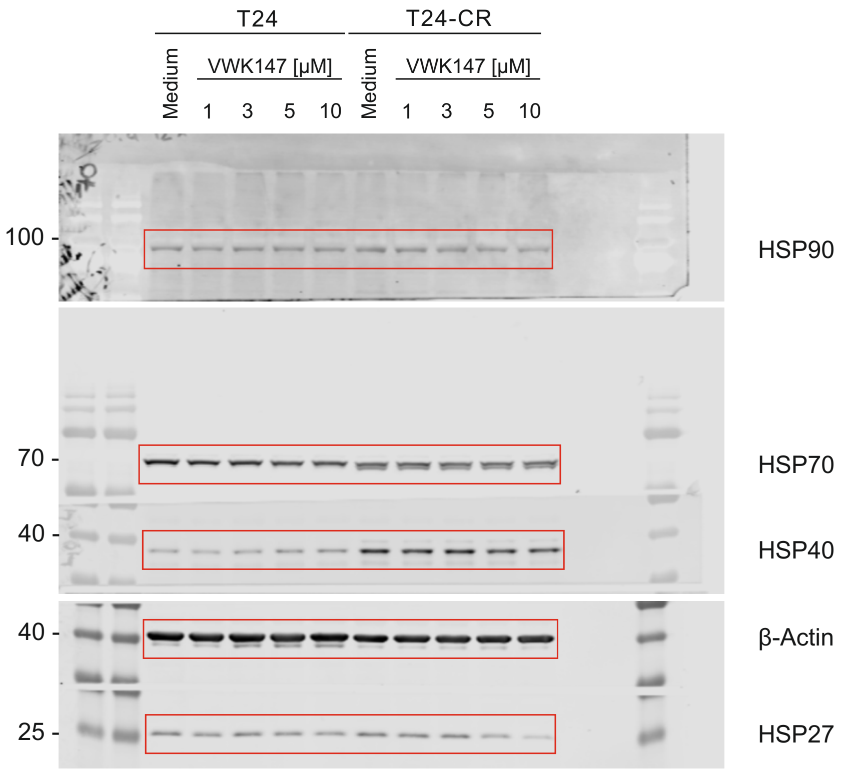

Figure 4D:

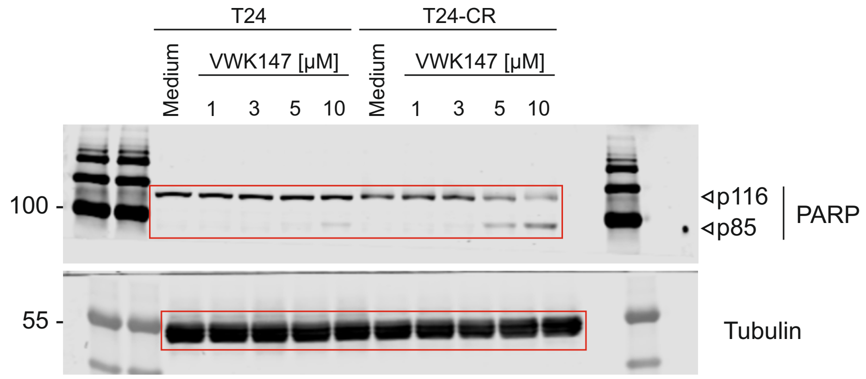

Figure 4E:

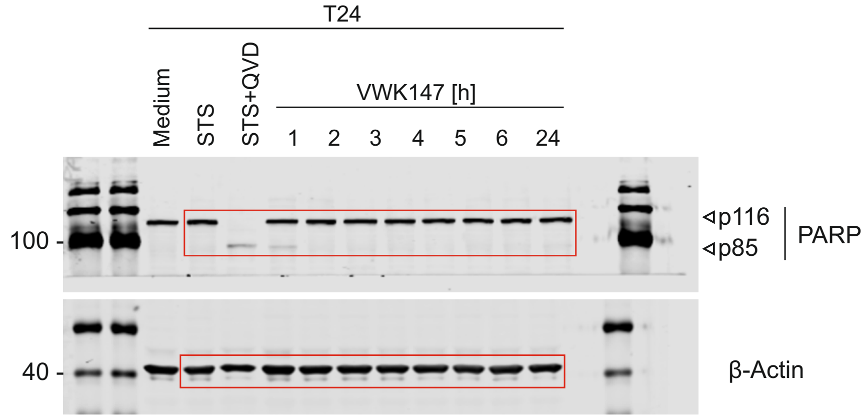

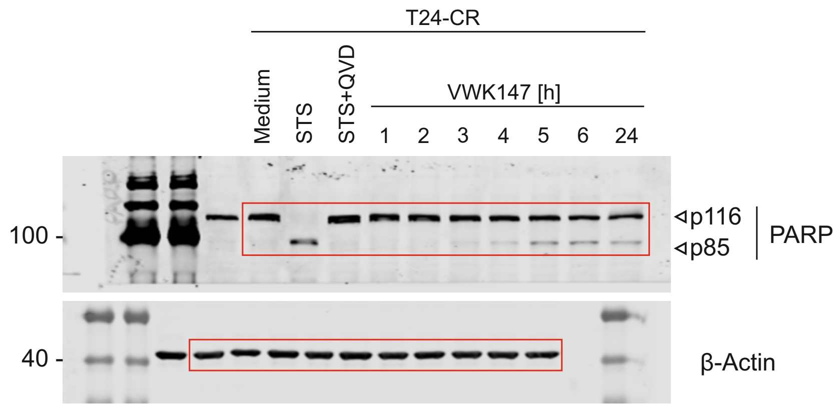

Figure 5A:

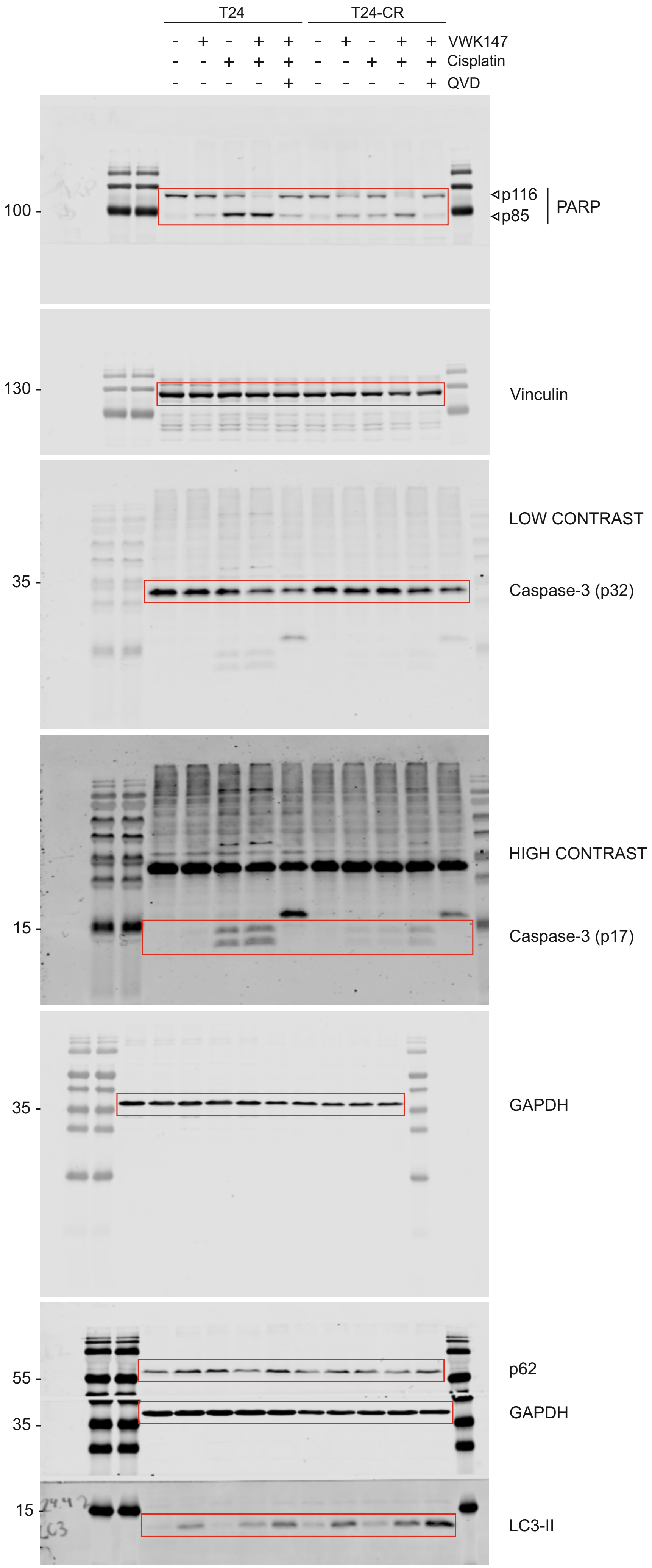

T24
T24-CR
- + - + + - + - + + VWK147
- - + + + - - + + + Cisplatin
- - - - + - - - - + QVD
100 -
◁p116
◁p85
PARP
130 -
Vinculin
LOW CONTRAST
35 -
Caspase-3 (p32)
HIGH CONTRAST
15 -
Caspase-3 (p17)
35 -
GAPDH
55 -
p62
35 -
GAPDH
15 -
LC3-II

Figure 6A:

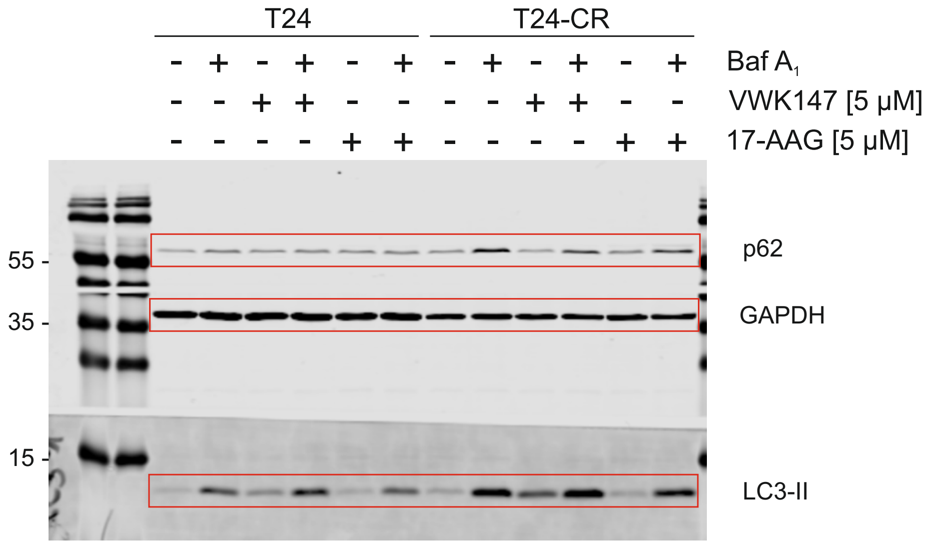

Figure 6B:

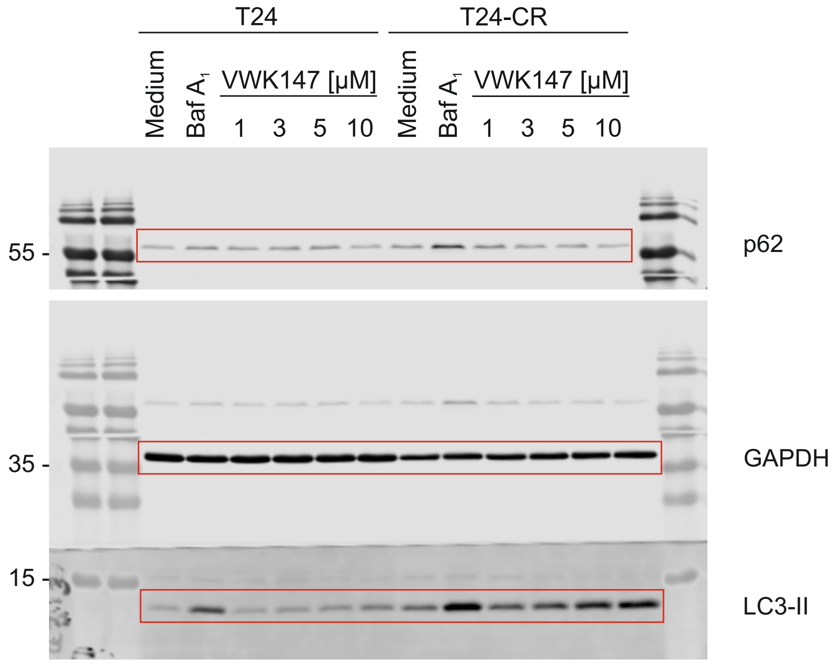

Figure 7B:

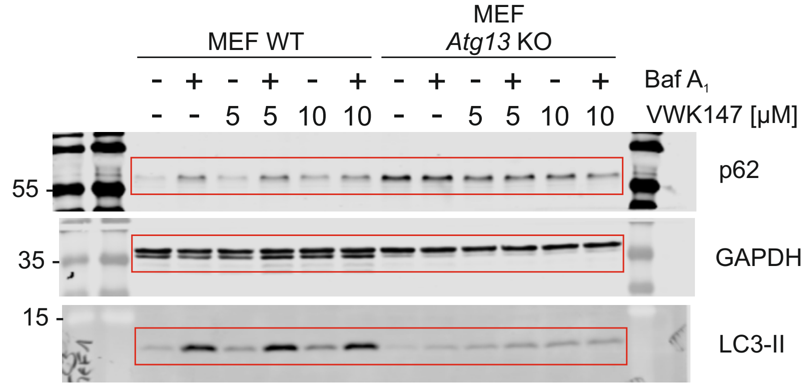

Figure 7C:

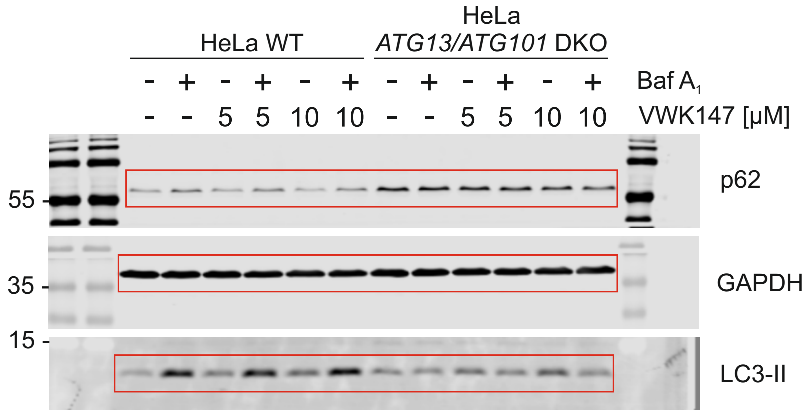

Figure 7D:

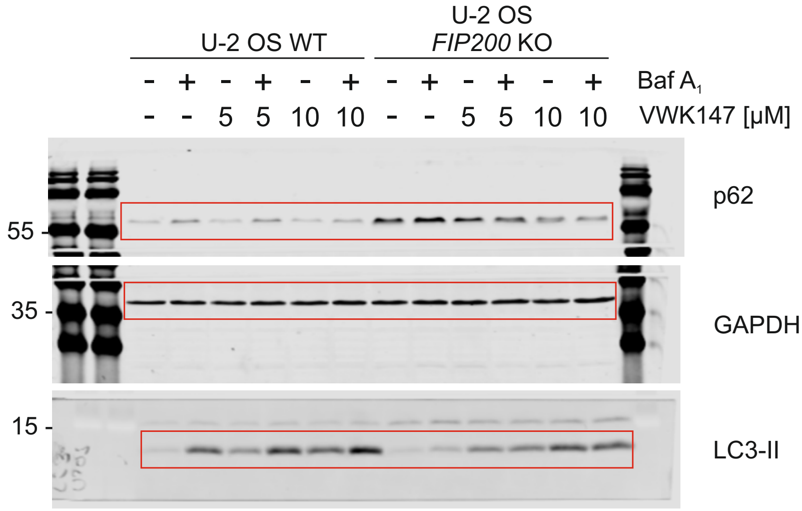

Suppl. Figure 2A and B:

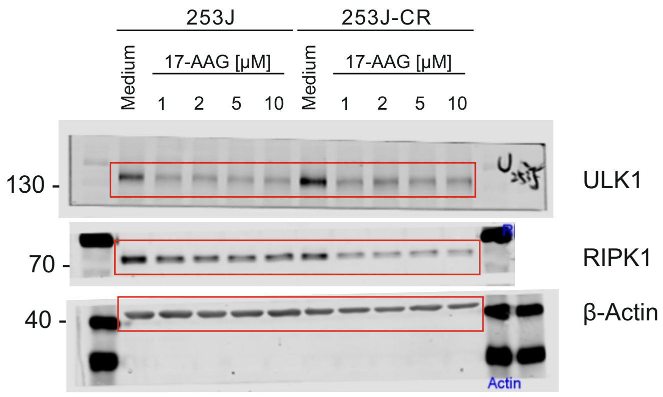

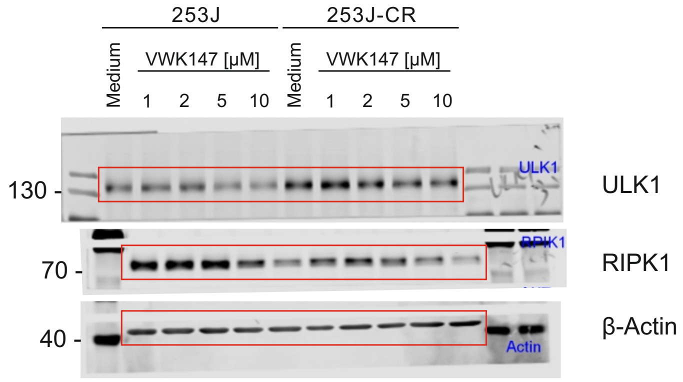

Suppl. Figure 2C:

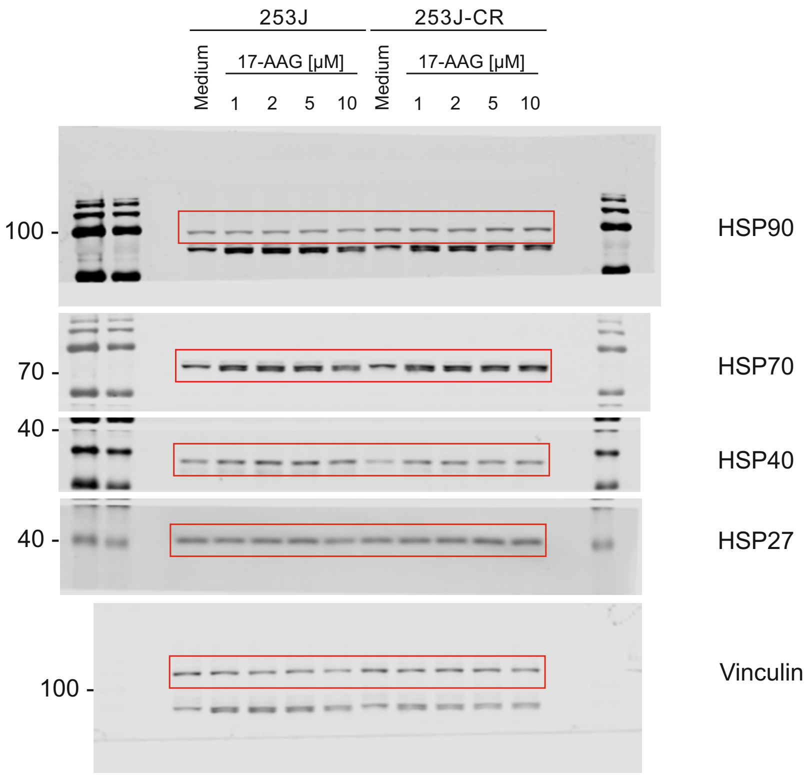

Suppl. Figure 2D:

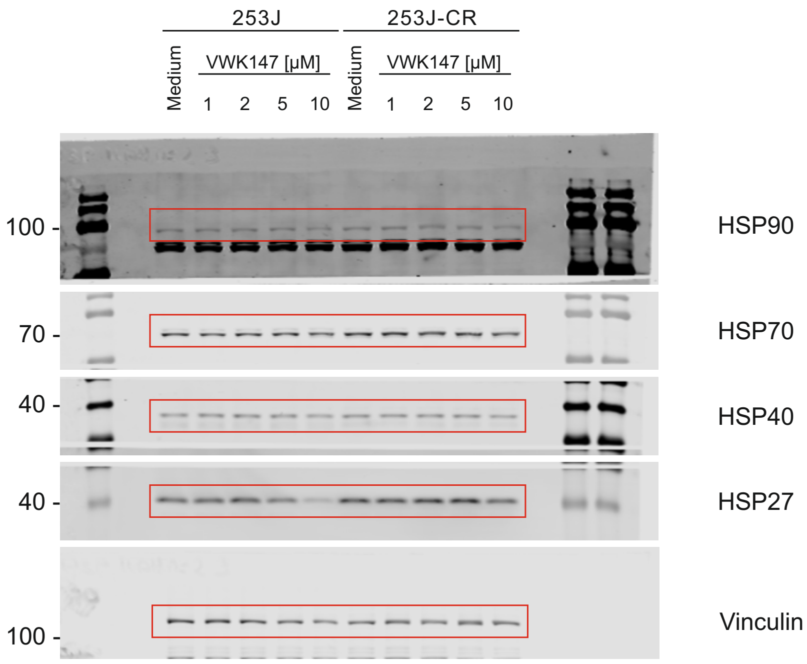

Suppl. Figure 4A:

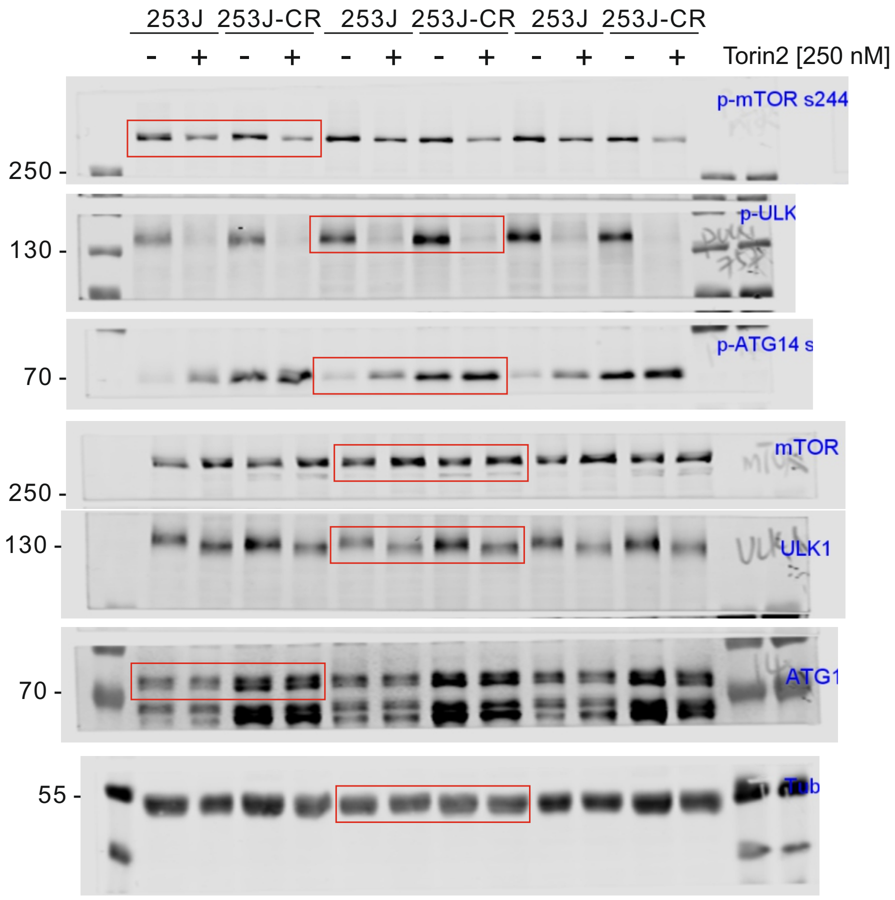

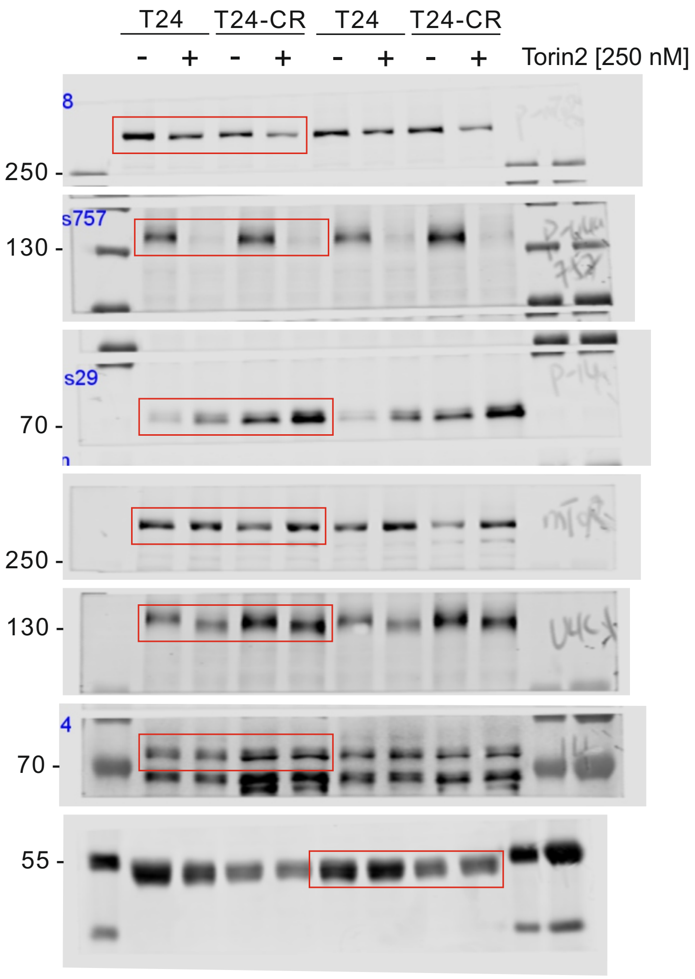

Suppl. Figure 4B:

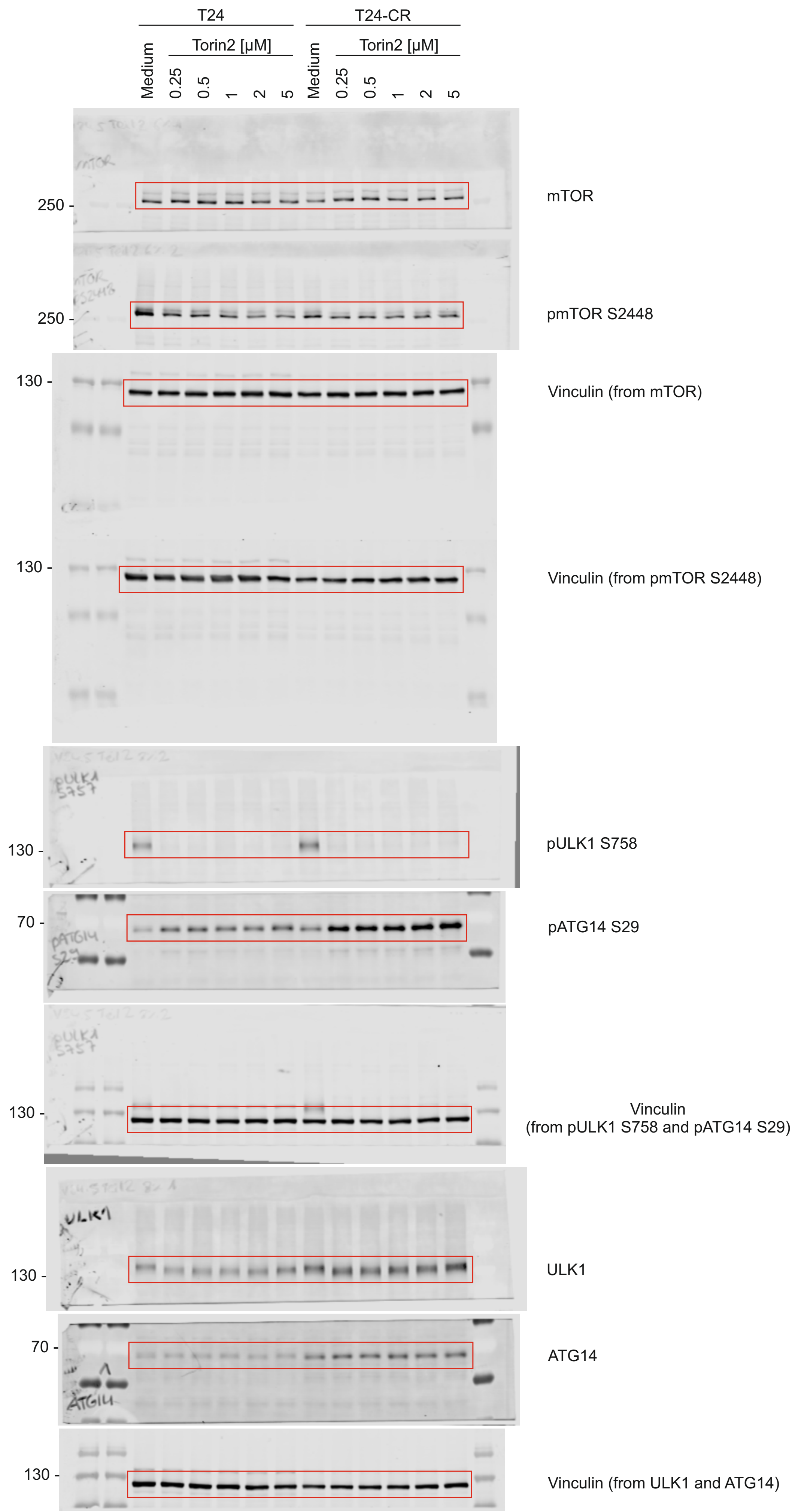

Suppl. Figure 5A:

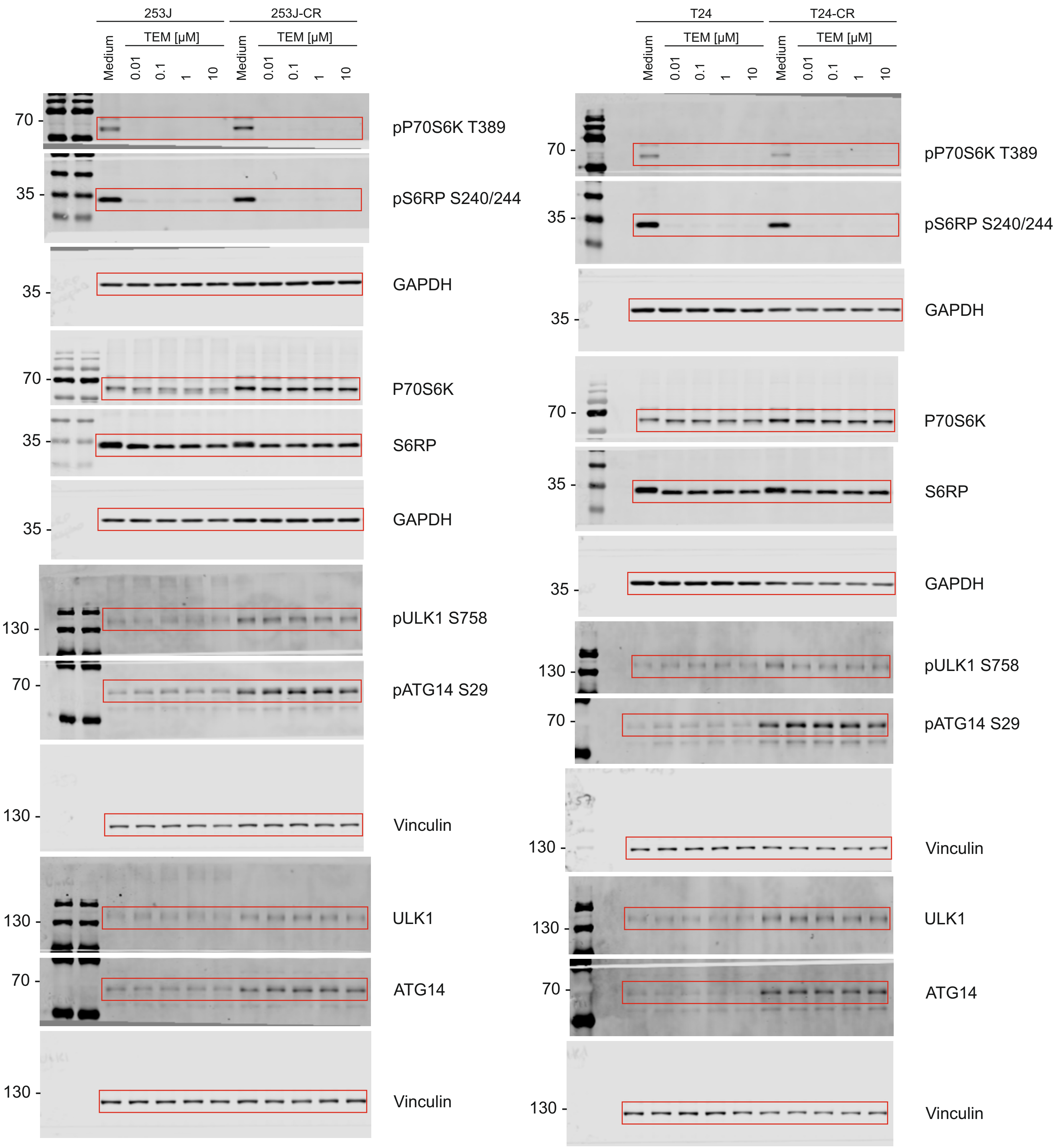

Suppl. Figure 7A:

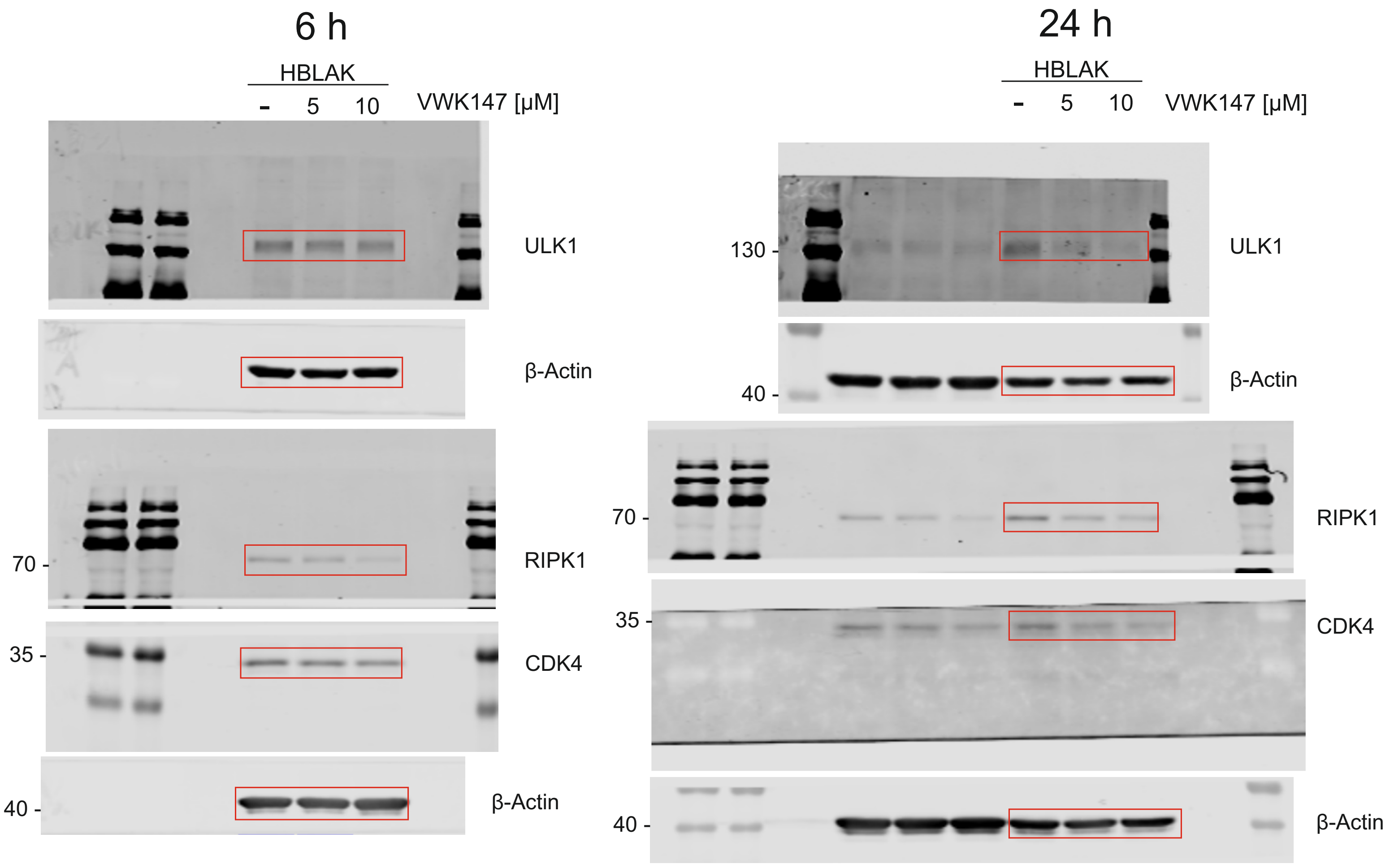

Suppl. Figure 7B:

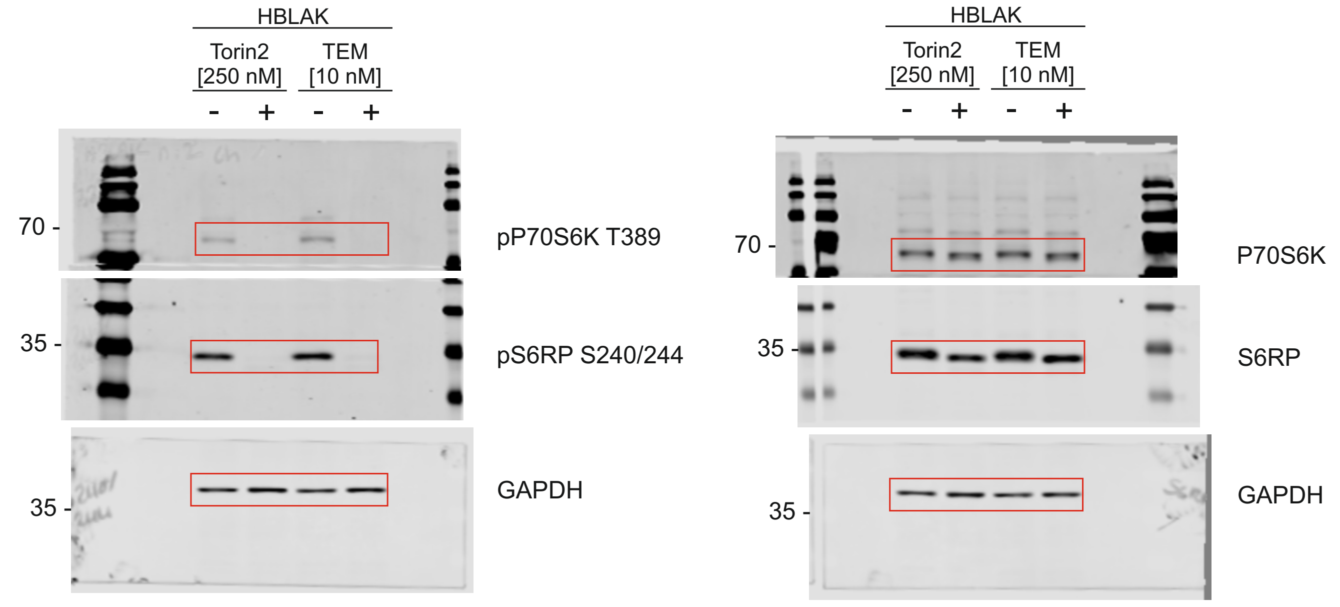

Suppl. Figure 9A:

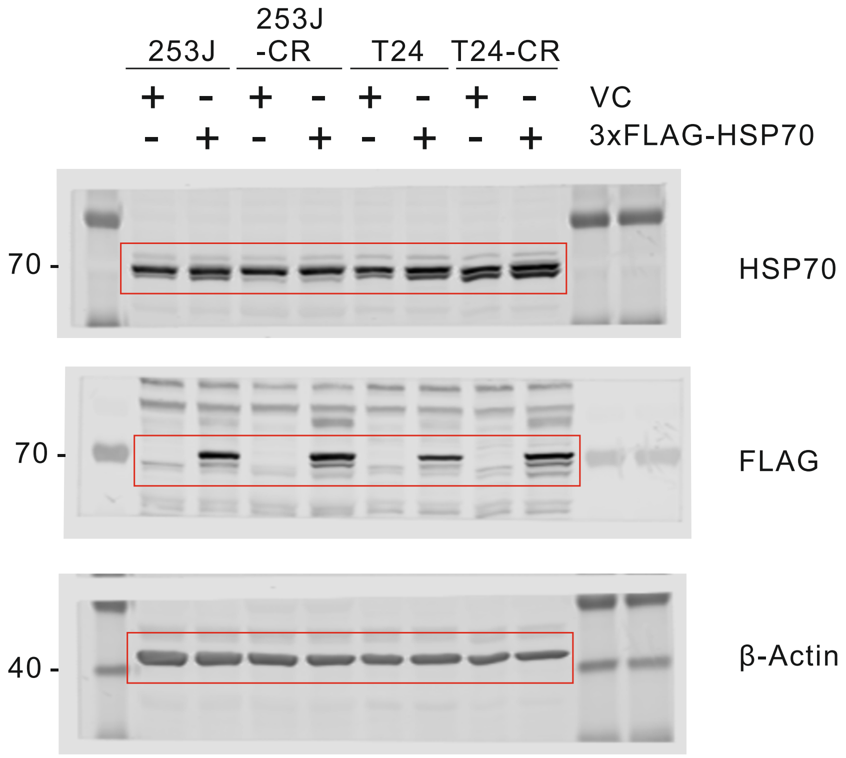

Suppl. Figure 11A:

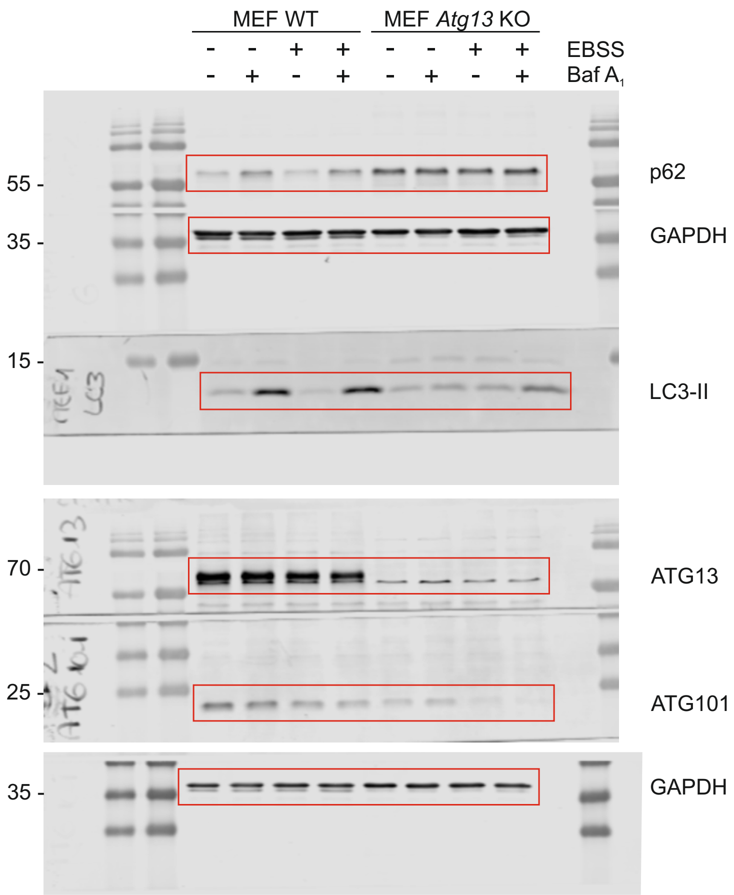

Suppl. Figure 11B:

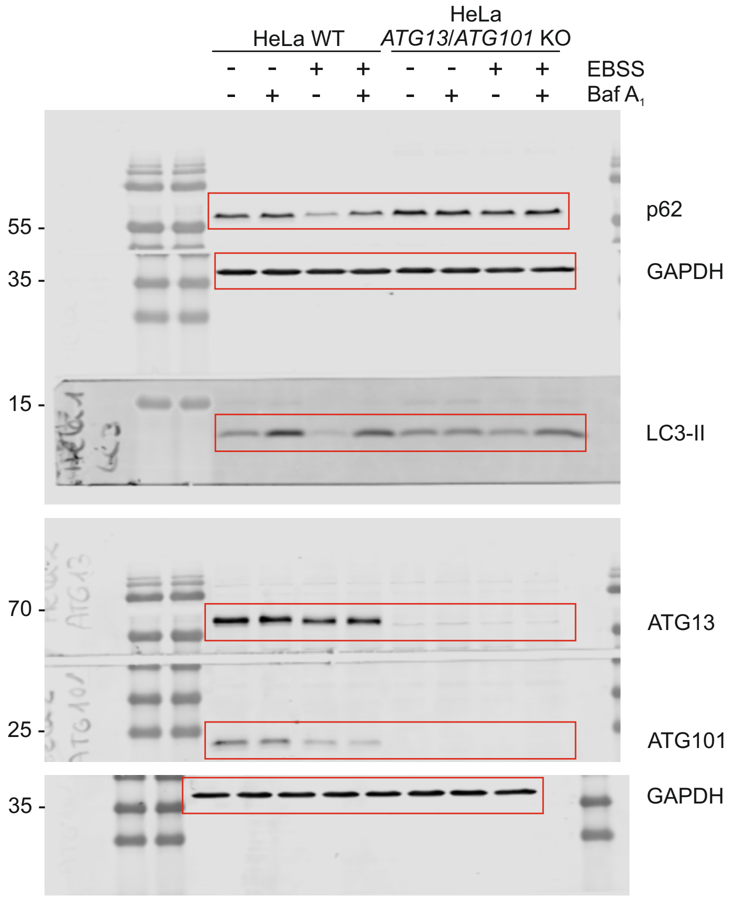

Suppl. Figure 11C:

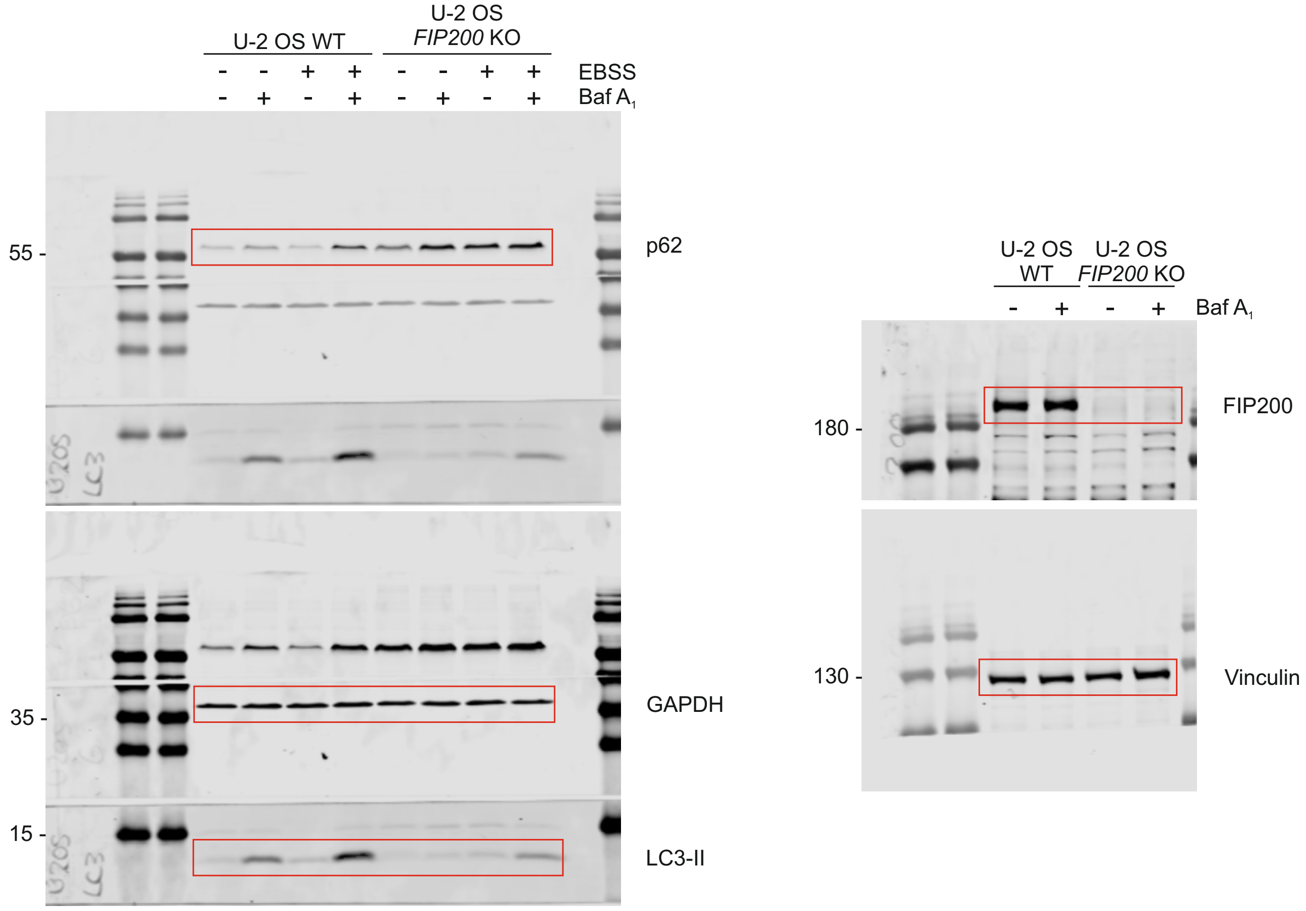

Suppl. Figure 12A:

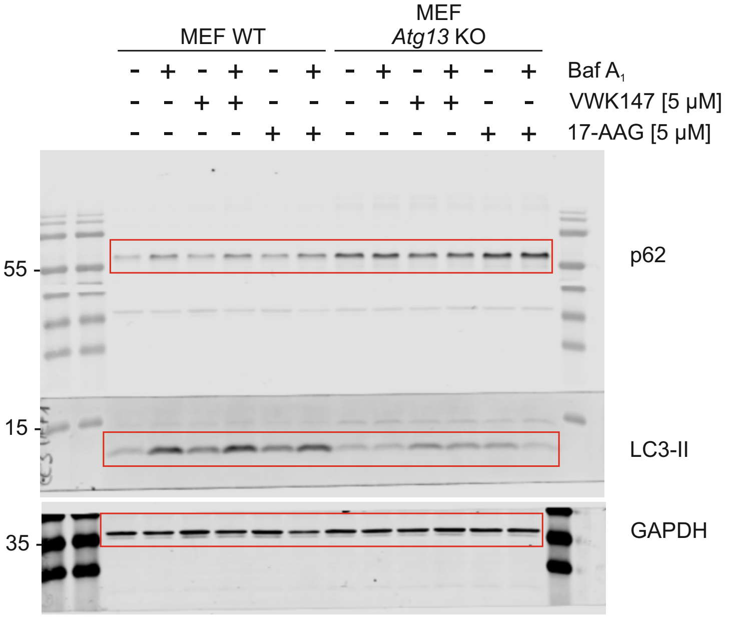

Suppl. Figure 12B:

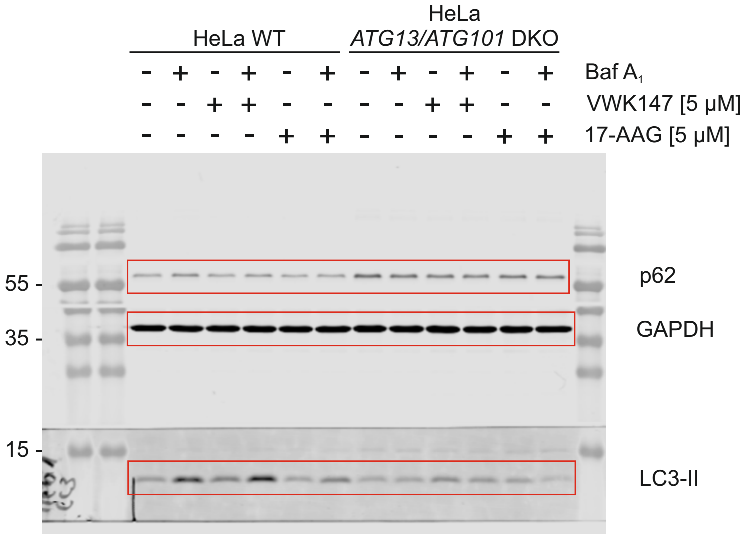

Suppl. Figure 12C:

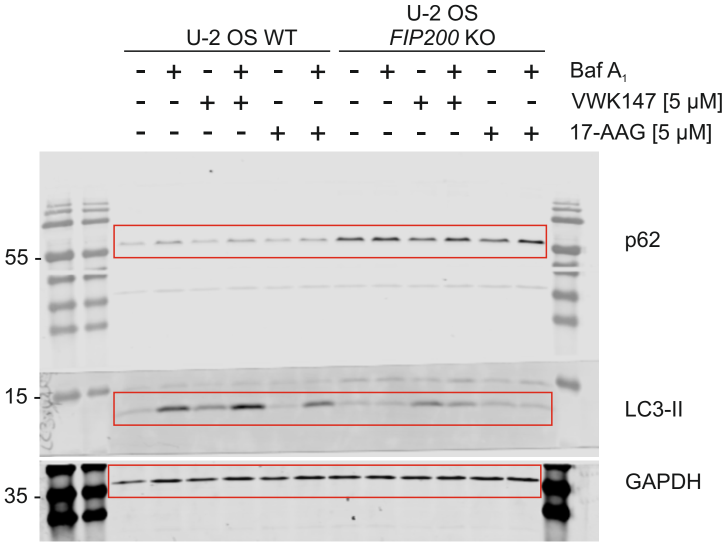

Suppl. Figure 13A:

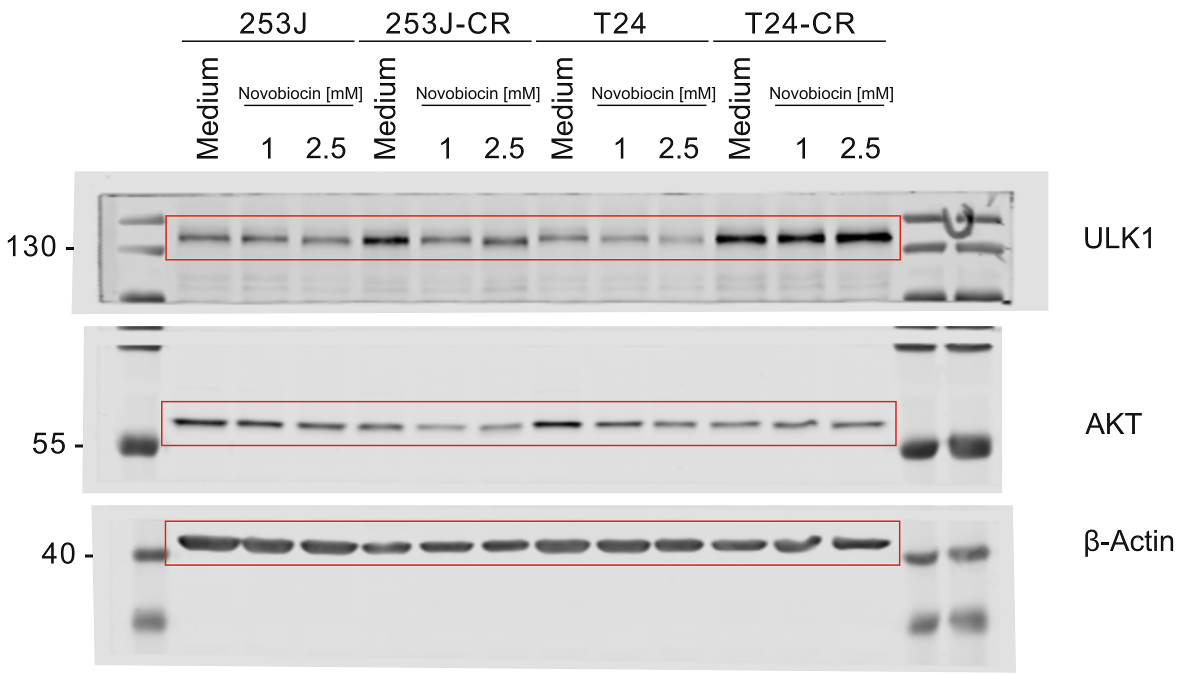

Suppl. Figure 13B:

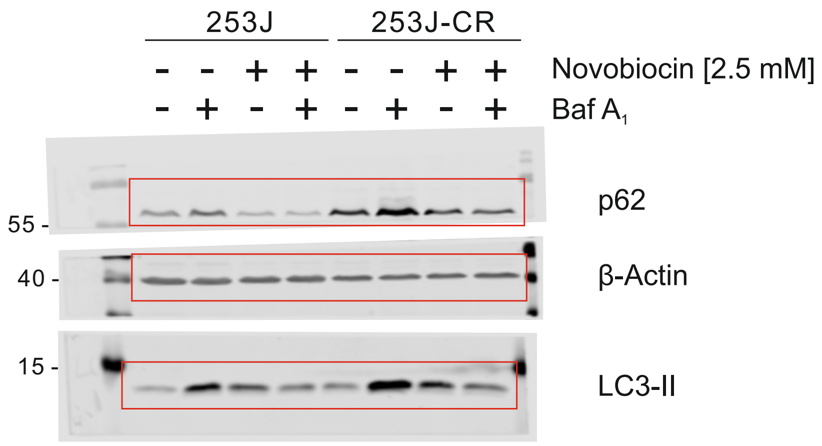

Suppl. Figure 13C:

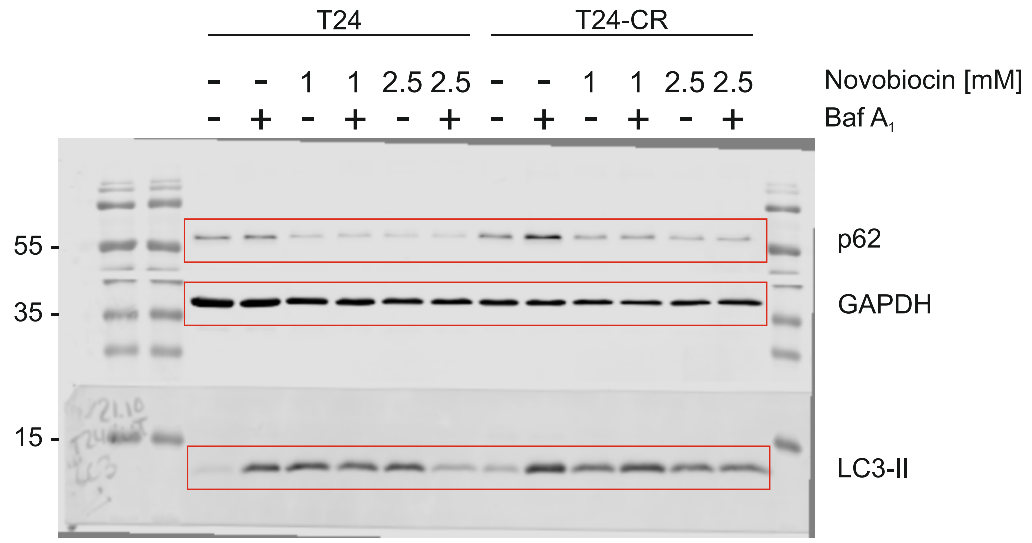

Suppl. Figure 13D:

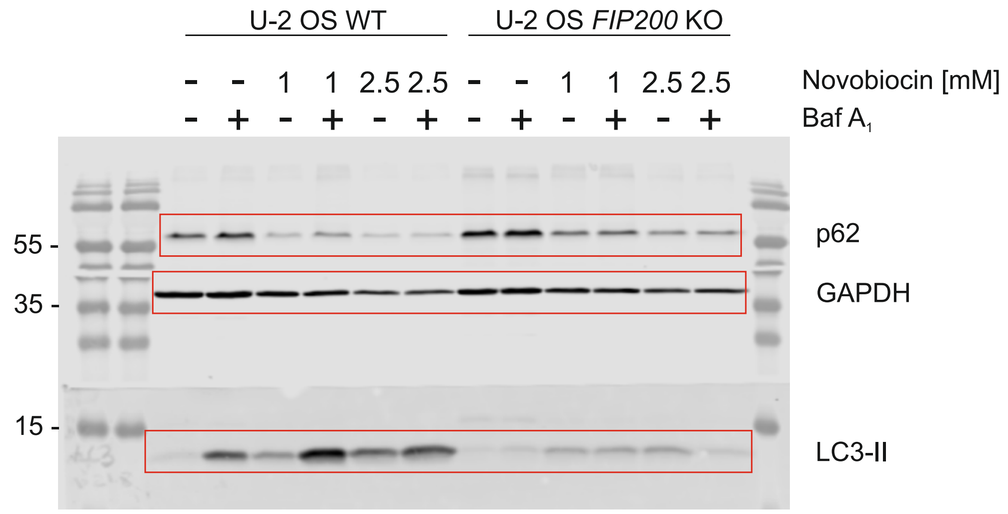

Supplement: Supplementary file 1 — Original data [file 41419_2025_8330_MOESM1_ESM.pdf]
