# Supplementary material for: Small-molecule inhibitor of C‑terminal HSP90 dimerization modulates autophagy and functions synergistically with mTOR inhibition to kill cisplatin-resistant cancer cells
Source: Cell Death Dis. 2025 Dec 23;17(1):130. doi: 10.1038/s41419-025-08330-4 (PMC12848304; doi:10.1038/s41419-025-08330-4)
Supplement: Supplementary file 2 — Supporting Information [file 41419_2025_8330_MOESM2_ESM.pdf]

Supporting Information

Small-molecule inhibitor of C-terminal HSP90 dimerization modulates autophagy and functions synergistically with mTOR inhibition to kill cisplatin-resistant cancer cells

Céline David[1,#], Yadong Sun[1,2,#], Vitalij Woloschin[3,#], Melina Vogt[4], Niklas Dienstbier[4], Annabelle Friedrich[1], Karina S. Krings[1], David Schlütermann[1], Lena Berning[1], Beate Lungerich[3], Seda Akgün[1], María José Mendiburo[1], Christoph G.W. Gertzen[3,5], Arndt Borkhardt[4], Sebastian Wesselborg[1], Holger Gohlke[3,6], Sanil Bhatia[4,*], Thomas Kurz[3,*], Björn Stork[1,*]

[1]Institute of Molecular Medicine I, Medical Faculty and University Hospital Düsseldorf, Heinrich Heine University, Düsseldorf 40225, Germany

[2]Department of Urology, Weifang People's Hospital, Shandong Second Medical University, Weifang, 261000, China.

[3]Heinrich Heine University Düsseldorf, Faculty of Mathematics and Natural Sciences, Institute for Pharmaceutical and Medicinal Chemistry, Düsseldorf 40225, Germany

[4]Department of Pediatric Oncology, Hematology and Clinical Immunology, Medical Faculty and University Hospital Düsseldorf, Heinrich Heine University, Düsseldorf 40225, Germany

[5]Heinrich Heine University Düsseldorf, Center for Structural Studies (CSS), Düsseldorf 40225, Germany

[6]Institute of Bio- and Geosciences (IBG-4: Bioinformatics), Forschungszentrum Jülich GmbH, Jülich 52425, Germany

#shared first authorship

*Correspondence: bjoern.stork@hhu.de; thomas.kurz@hhu.de; sanil.bhatia@med.uni-duesseldorf.de

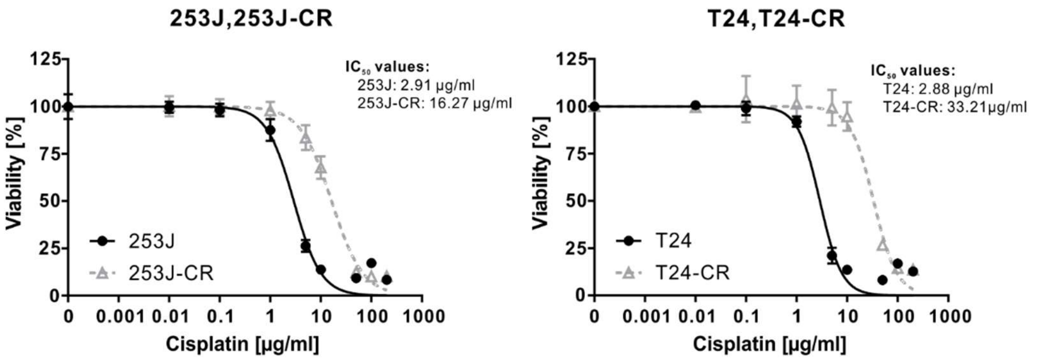

Suppl. Figure S1: Characterization of cisplatin-sensitive and -resistant urothelial bladder carcinoma cell lines. 253J, 253J-CR, T24 and T24-CR urothelial carcinoma cells were treated with indicated concentrations of cisplatin for 72 h. After treatment, cell viability was measured using Alamar Blue assay. Results are shown as means ± SD of three independent experiments performed in triplicates for each treatment.

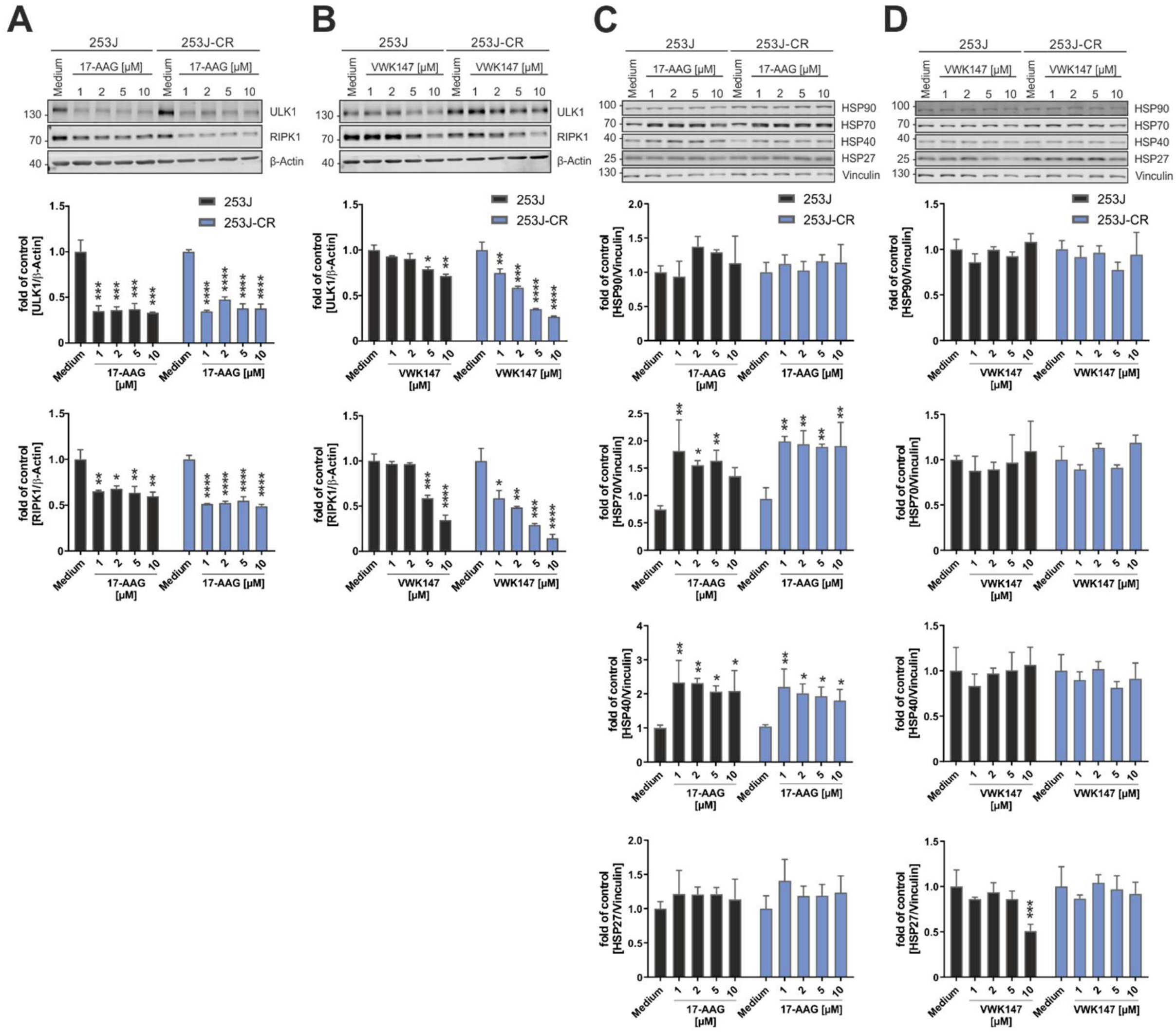

Suppl. Figure S2: VWK147 destabilizes HSP90 clients but does not induce a heat-shock response. (**A-D**) 253J and 253J-CR urothelial carcinoma cells were treated with the indicated concentrations of 17-AAG or VWK147 for 6 h. After treatment, the cells were lysed, and cellular lysates were immunoblotted for ULK1, RIPK1, Actin, HSP90, HSP70, HSP40, HSP27, and Vinculin, respectively. One representative immunoblot is shown. The quantifications of indicated ratios are from three independent experiments (means + SD). P values were determined by ordinary one-way ANOVA with Dunnett´s post hoc or multiple comparisons test (comparison to the solvent control of the respective cell line). *p ≤ 0.05; **p ≤ 0.01; ***p ≤ 0.001; ****p ≤ 0.0001.

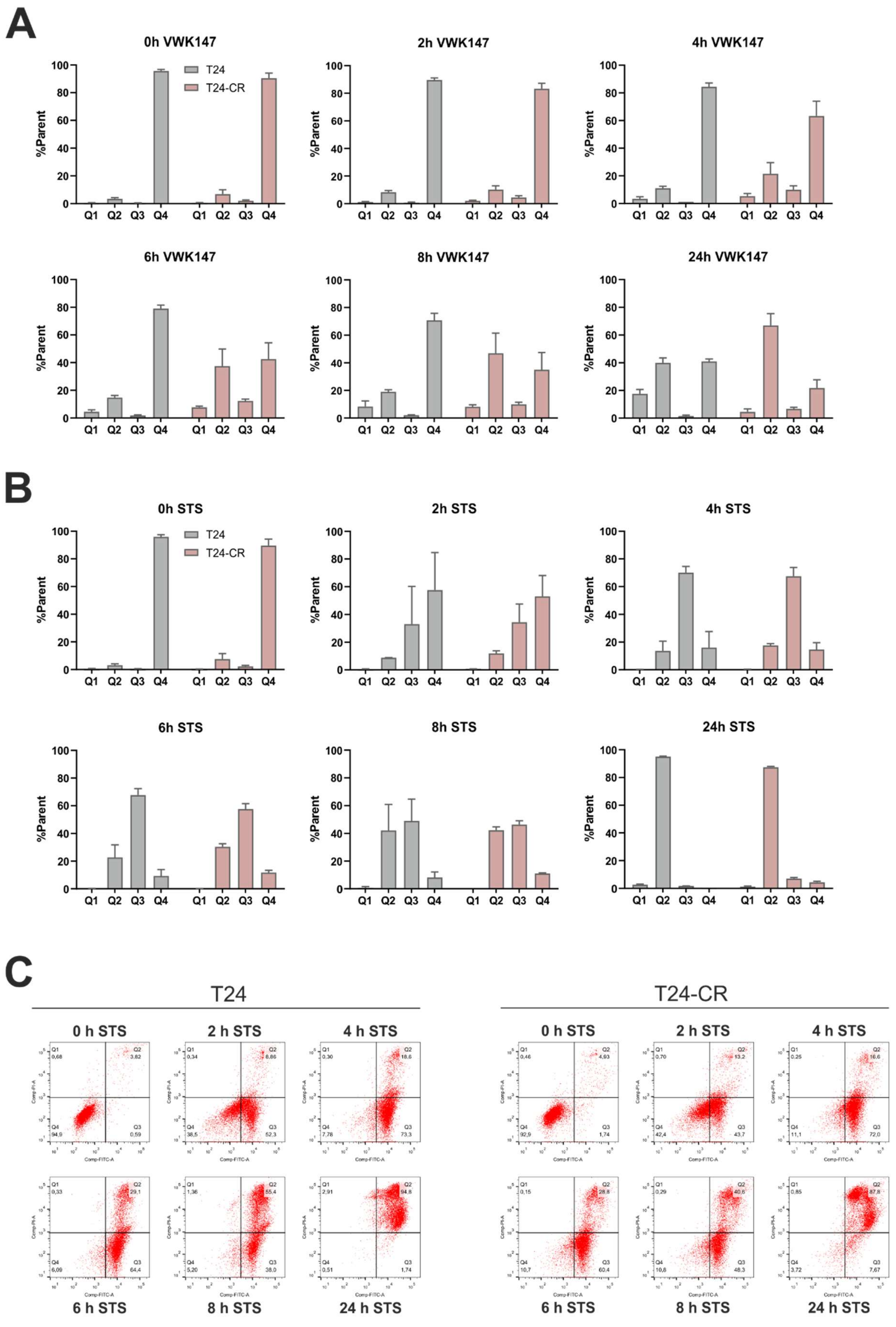
A
0h VWK147
2h VWK147
4h VWK147
6h VWK147
8h VWK147
24h VWK147
T24
T24-CR
%Parent
B
0h STS
2h STS
4h STS
6h STS
8h STS
24h STS
C
T24
T24-CR
0 h STS
2 h STS
4 h STS
6 h STS
8 h STS
24 h STS

◄ Suppl. Figure S3: Characterization of VWK147-induced cell death and staurosporine-induced apoptosis as a positive assay control in T24 and T24-CR urothelial carcinoma cells via FITC-Annexin V and PI co-staining. T24 and T24-CR urothelial carcinoma cells were treated with (**A**) 10 µM VWK147 or (**B, C**) 2.5 µM STS for the indicated periods of time. DMSO was used as a solvent control. After treatment, the cells were collected, stained with FITC-Annexin V and propidium iodide (PI) and analyzed by flow cytometry. In (**C**), one representative experiment is shown as dot plots. The quadrant Q4 represents living cells (Annexin V and PI negative), the quadrant Q3 early apoptotic cells (Annexin V positive, PI negative), the quadrant Q2 late apoptotic cells (Annexin V and PI positive) and the quadrant Q1 necrotic or dead cells (Annexin V negative and PI positive). For (**A**) and (**B**), the average of the population percentage in each quadrant from two (**B**) to three (**A**) independent experiments are represented in bar diagrams (means + SD). 'Parent' refers to the population remaining after gating out doublets and debris.

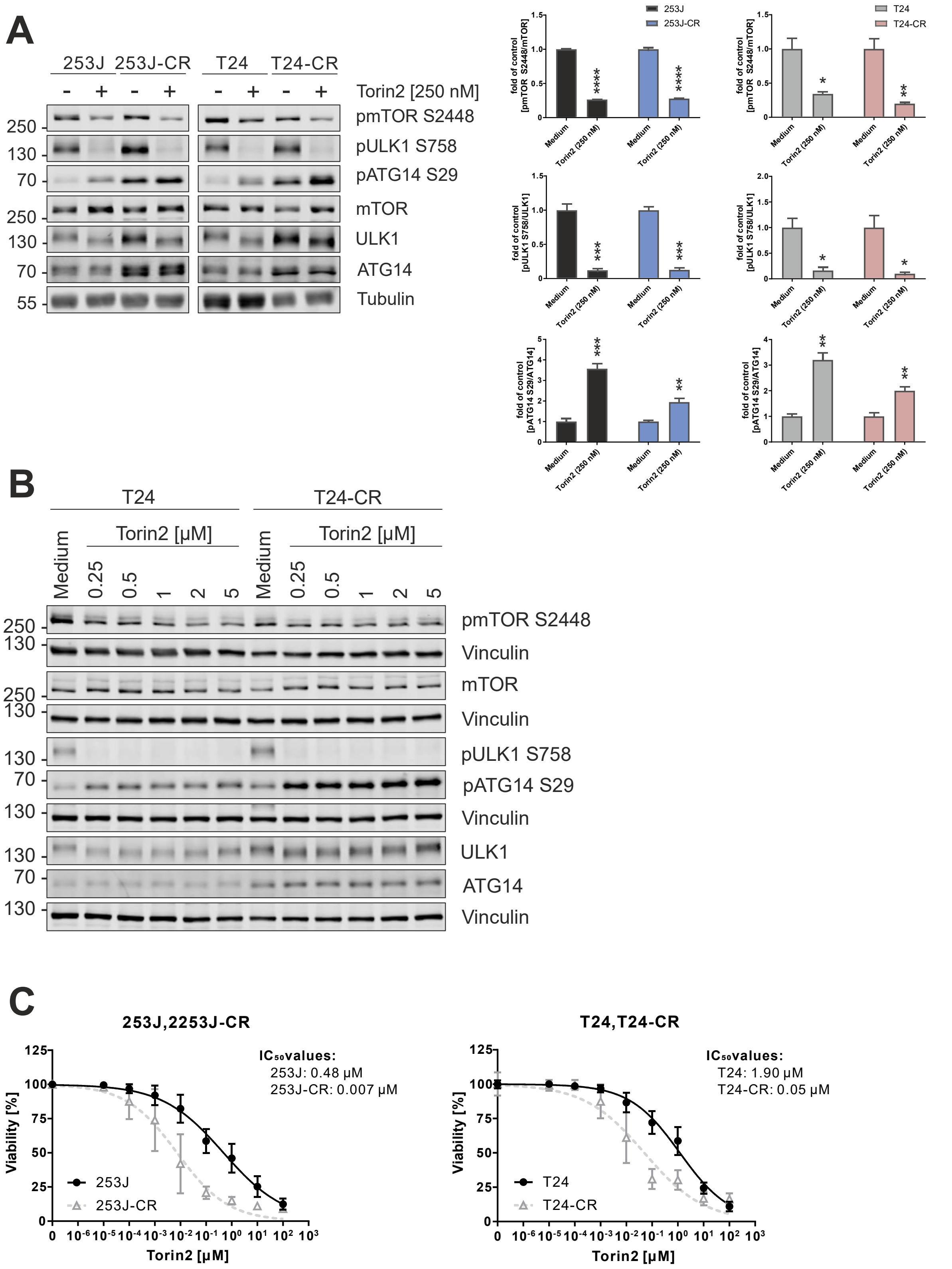

Suppl. Figure S4: Characterization of Torin2 efficacy in urothelial bladder carcinoma cell lines. (**A**) 253J, 253J-CR, T24 and T24-CR urothelial carcinoma cells were treated with 250 nM Torin2 for 2 h. After treatment, the cells were lysed, and cellular lysates were immunoblotted for phospho-mTOR

Ser2448, mTOR, phospho-ULK1 Ser758, ULK1, phospho-ATG14 Ser29, ATG14 and tubulin. One representative immunoblot is shown. The quantifications of indicated ratios are from three independent experiments (means + SD). P values were determined by unpaired t test (comparison to the solvent control of the respective cell line). *$p \leq 0.05$; **$p \leq 0.01$; ***$p \leq 0.001$; ****$p \leq 0.0001$. (**B**) T24 and T24-CR urothelial carcinoma cells were treated with indicated concentrations of Torin2 for 24 h. After treatment, the cells were lysed, and cellular lysates were immunoblotted for phospho-mTOR Ser2448, mTOR, phospho-ULK1 Ser758, ULK1, phospho-ATG14 Ser29, ATG14 and Vinculin (n=1). (**C**) 253J, 253J-CR, T24 and T24-CR urothelial carcinoma cells were treated with indicated concentrations of Torin2 for 72 h. After treatment, cell viability was measured using Alamar Blue assay. Results are shown as means ± SD of three independent experiments performed in triplicates for each treatment.

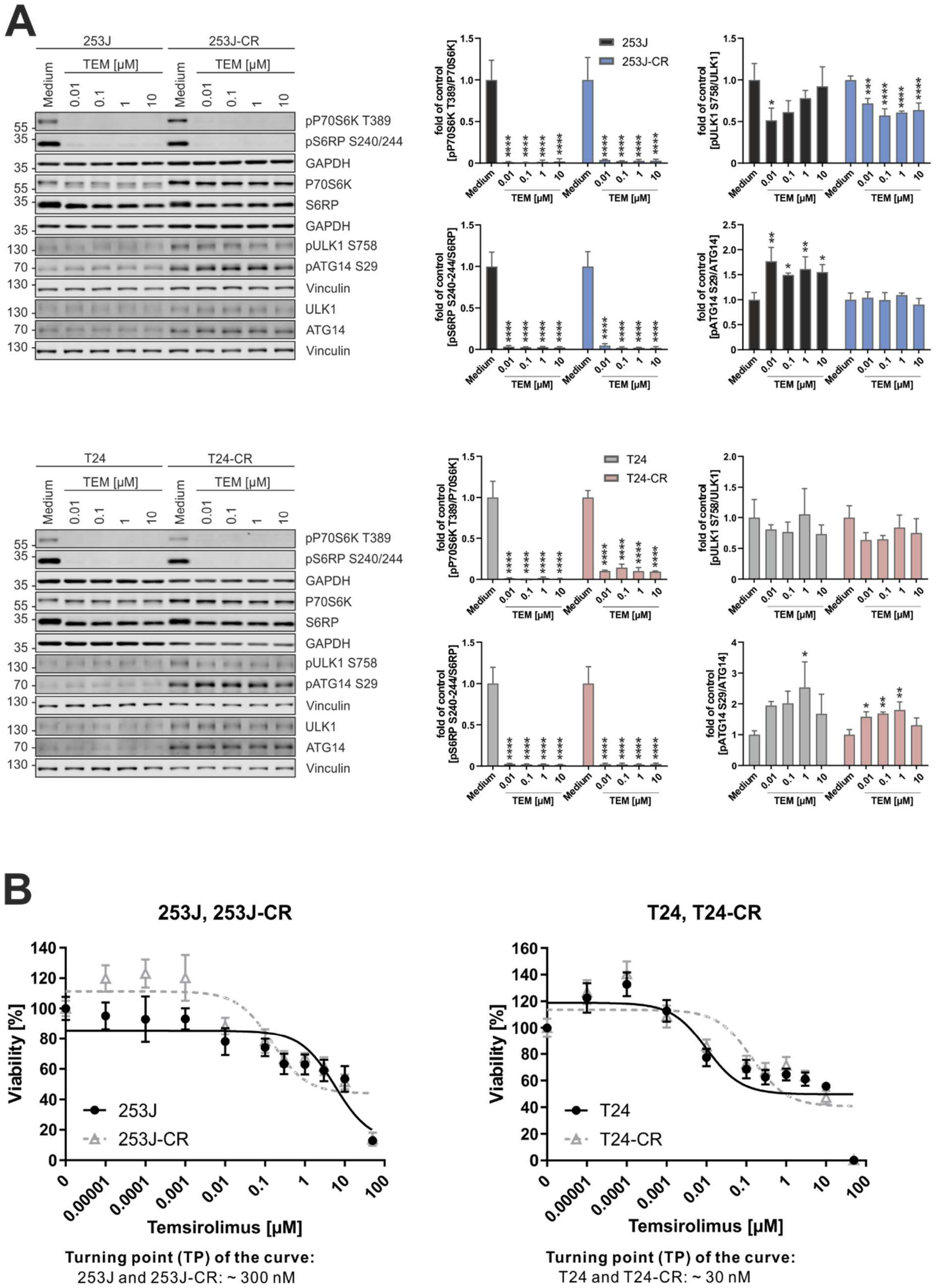

Suppl. Figure S5: Characterization of Temsirolimus efficacy in urothelial carcinoma cell lines. (**A**) 253J, 253J-CR, T24 and T24-CR urothelial carcinoma cells were treated with indicated concentrations of Temsirolimus for 6 h. After treatment, the cells were lysed, and cellular lysates were

immunoblotted for phospho-P70 S6 kinase Thr389, P70 S6 kinase, phospho-S6 ribosomal protein Ser 240/244, S6 ribosomal protein, phospho-ULK1 Ser758, ULK1, phospho-ATG14 Ser29, ATG14, GAPDH and Vinculin. One representative immunoblot is shown. The quantifications of indicated ratios are from three independent experiments (means + SD). P values were determined by ordinary one-way ANOVA with Dunnett's multiple comparisons test (comparison to the solvent control of the respective cell line). *p ≤ 0.05; **p ≤ 0.01; ***p ≤ 0.001; ****p ≤ 0.0001. (**B**) 253J, 253J-CR, T24 and T24-CR urothelial carcinoma cells were treated with indicated concentrations of Temsirolimus for 72 h. After treatment, cell viability was measured using CellTiter-Glo Luminescent Cell Viability assay. Results are shown as means ± SD of three independents experiments performed in triplicates for each treatment.

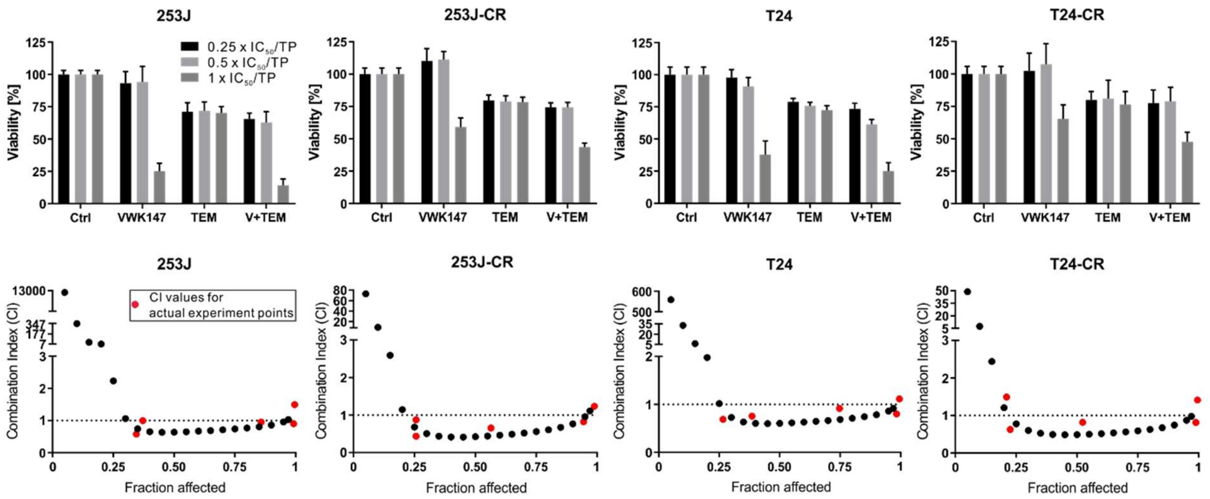

Suppl. Figure S6: VWK147 acts synergistically with the clinically approved mTOR inhibitor Temsirolimus in urothelial carcinoma cell lines. 253J, 253J-CR, T24 and T24-CR urothelial carcinoma cells were treated with VWK147 combined with Temsirolimus (0.25 x, 0.5 x, 1 x, 2 x and 4 x IC_{50} or turning point of the viability curve (TP)) for 72 h. The approximate IC_{50} values or TPs were determined based on the previous mono-treatment. The cell viability was measured using CellTiter-Glo Luminescent Cell Viability assay. Viability results are depicted in bar diagrams as means + SD of three independent experiments performed in triplicates for each treatment (only 0.25 x, 0.5 x and 1 x IC_{50} or TP are shown). The combination index values were calculated using CompuSyn. The actual experiment points are indicated as red dots whereas simulation dots processed by CompuSyn are indicated as black dots. The combination effects of VWK147 and Temsirolimus were determined synergistic (CI < 1).

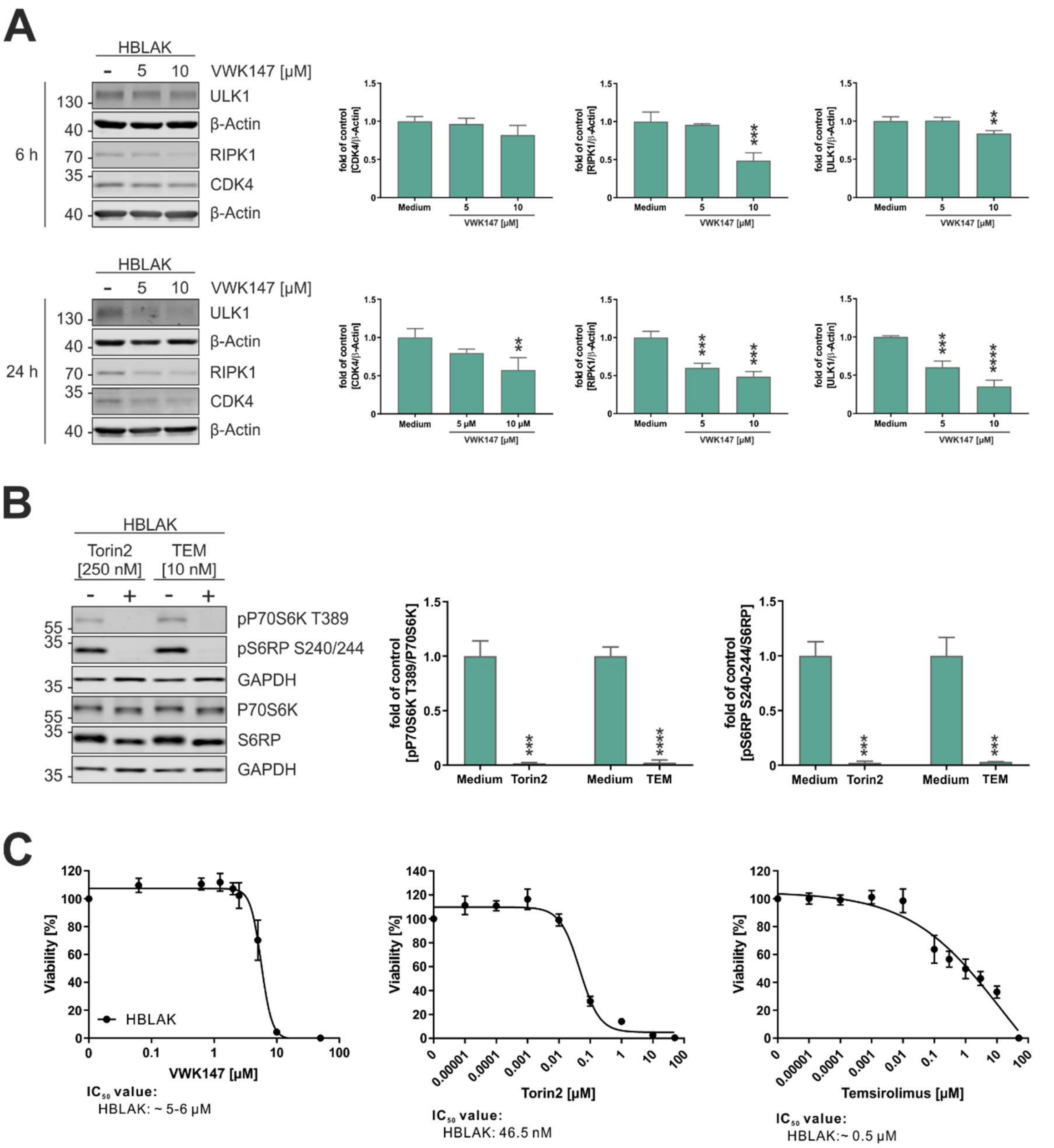

Suppl. Figure S7: Characterization of VWK147, Temsirolimus and Torin2 efficacy in the uroepithelial cell line HBLAK. (**A**) HBLAK uroepithelial cells were treated with indicated concentrations of VWK147 for 6 h and 24 h. After treatment, the cells were lysed, and cellular lysates were immunoblotted for ULK1, RIPK1, CDK4 and β-Actin. One representative immunoblot is shown. The quantifications of indicated ratios are from three independent experiments (means + SD). P values were determined by ordinary one-way ANOVA with Dunnett's multiple comparisons test (comparison to the solvent control of the respective cell line). **p ≤ 0.01; ***p ≤ 0.001; ****p ≤ 0.0001. (**B**) HBLAK uroepithelial cells were treated with indicated concentrations of Torin2 and Temsirolimus for 6 h. After

treatment, the cells were lysed, and cellular lysates were immunoblotted for phospho-P70 S6 kinase Thr389, P70 S6 kinase, phospho-S6 ribosomal protein Ser 240/244, S6 ribosomal protein and GAPDH. One representative immunoblot is shown. The quantifications of indicated ratios are from three independent experiments (means + SD). P values were determined by a two-tailed unpaired t-test. ***p ≤ 0.001; ****p ≤ 0.0001. (**C**) HBLAK uroepithelial cells were treated with indicated concentrations of VWK147, Torin2 and Temsirolimus for 72 h. After treatment, cell viability was measured using CellTiter-Glo Luminescent Cell Viability assay. Results are shown as means ± SD of at least three independent experiments performed in triplicates for each treatment.

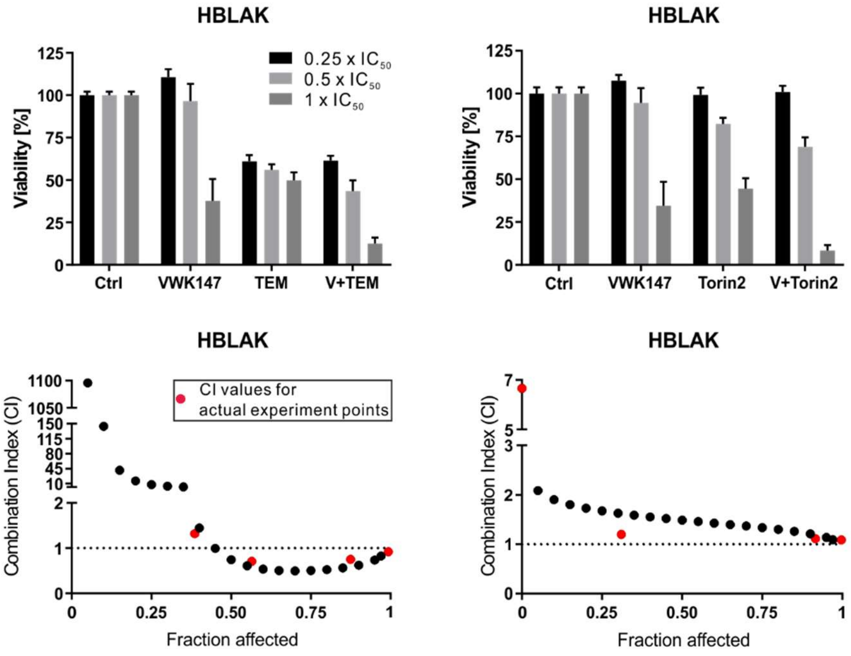

Suppl. Figure S8: VWK147 acts slightly synergistically with the clinically approved mTOR inhibitor Temsirolimus but not with Torin2 in the uroepithelial cell line HBLAK. HBLAK uroepithelial cells were treated with indicated concentrations of VWK147 combined with Temsirolimus or Torin2 (0.25 x, 0.5 x, 1 x and 2 x IC_{50}) for 72 h. The approximate IC_{50} values were determined based on the previous mono-treatment. The cell viability was measured using CellTiter-Glo Luminescent Cell Viability assay. Viability results are depicted in bar diagrams as means + SD of four independent experiments performed in triplicates for each treatment (only 0.25 x, 0.5 x and 1 x IC_{50} are shown). The combination index values were calculated using CompuSyn. The actual experiment points are indicated as red dots whereas simulation dots processed by CompuSyn are indicated as black dots. The combination effects of VWK147 and Temsirolimus were determined synergistic (CI < 1) and antagonistic (CI > 1).

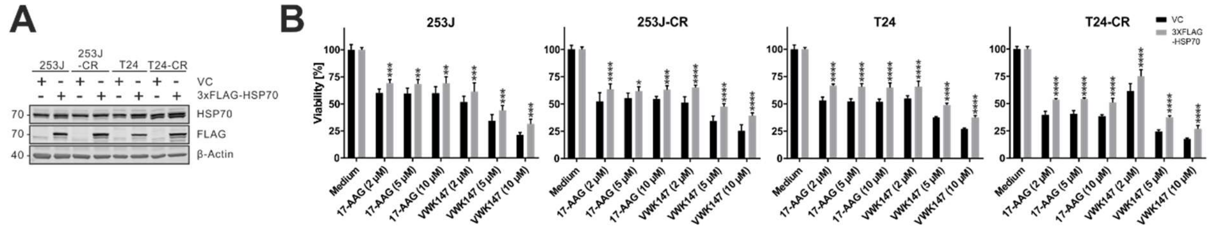

Suppl. Figure S9: Forced expression of HSP70 reduces cytotoxic effect of VWK147. (**A**) 253J, 253J-CR, T24 and T24-CR urothelial carcinoma cells transfected with either empty vector (VC) or cDNA encoding 3xFLAG-HSP70 were lysed and cellular lysates were immunoblotted for HSP70, FLAG and β-Actin. One representative immunoblot is shown. (**B**) 253J, 253J-CR, T24 and T24-CR urothelial carcinoma cells transfected with either empty vector (VC) or cDNA encoding 3xFLAG-HSP70-were treated with indicated concentrations of 17-AAG or VWK147 for 24 h. After treatment, cell viability was measured using MTT assay. Results are shown as means + SD of three independent experiments performed in triplicates for each treatment. P values were determined by ordinary two-way ANOVA with Sidak´s post hoc test. *$p \leq 0.05$; **$p \leq 0.01$; ***$p \leq 0.001$; ****$p \leq 0.0001$

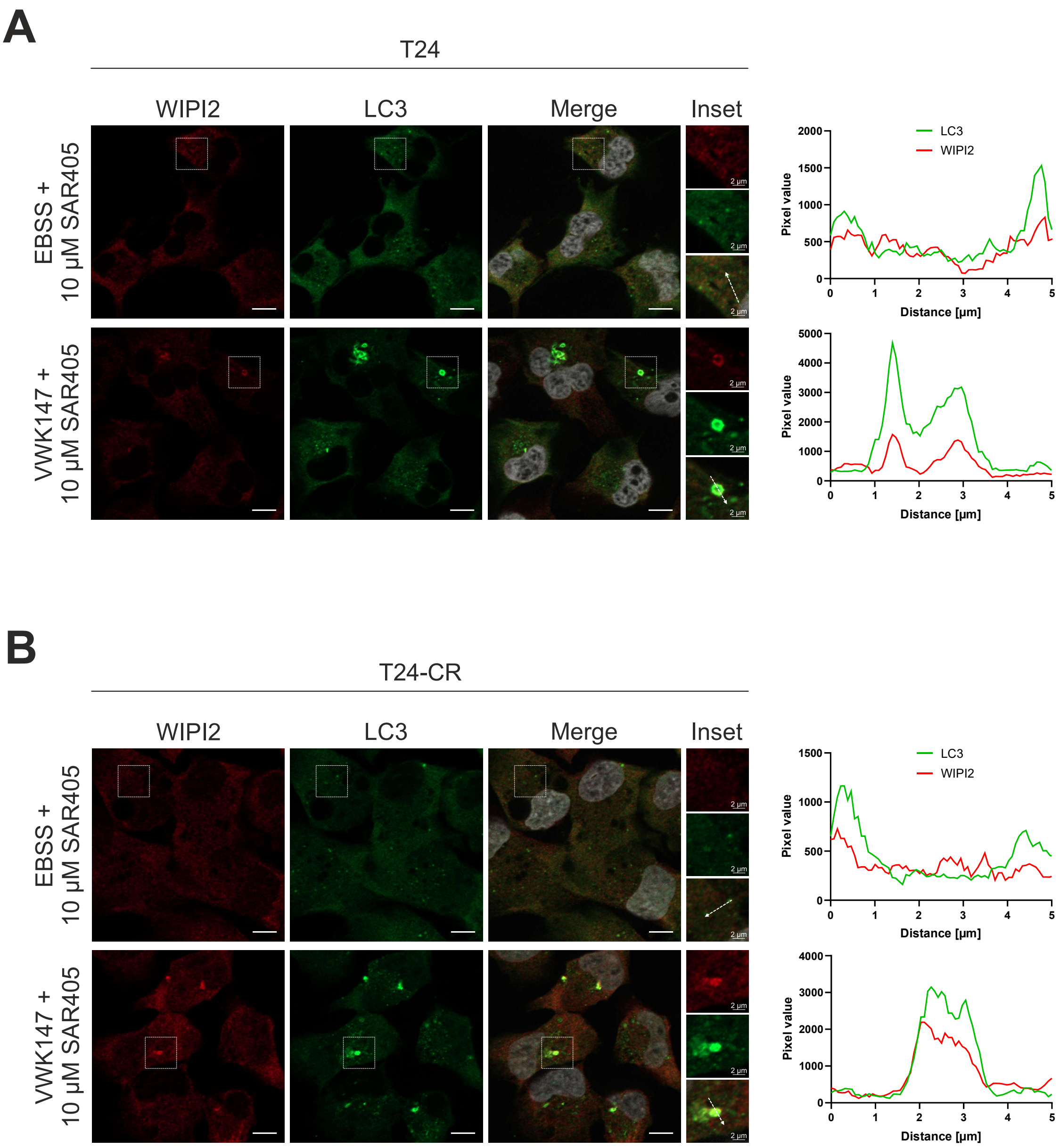

Suppl. Figure S10: VWK147 induces LC3-WIPI2 aggregates in T24 and T24-CR urothelial carcinoma cells. (**A**) T24 and (**B**) T24-CR urothelial carcinoma cells were grown on glass coverslips one or two days prior to treatment. Cells were treated with 10 µM SAR405 in combination with EBSS or 5 µM VWK147 for 4 h. Imaging was performed using a Zeiss Axio Observer 7 fluorescence microscope equipped with a 40x/1.4 Oil DIC M27 Plan-Apochromat objective and ApoTome 2. Representative sections are depicted. Scale bars: 10 µm and 2 µm. The line graphs represent the pixel intensities of the areas indicated by the respective dashed white arrows shown in the insets.

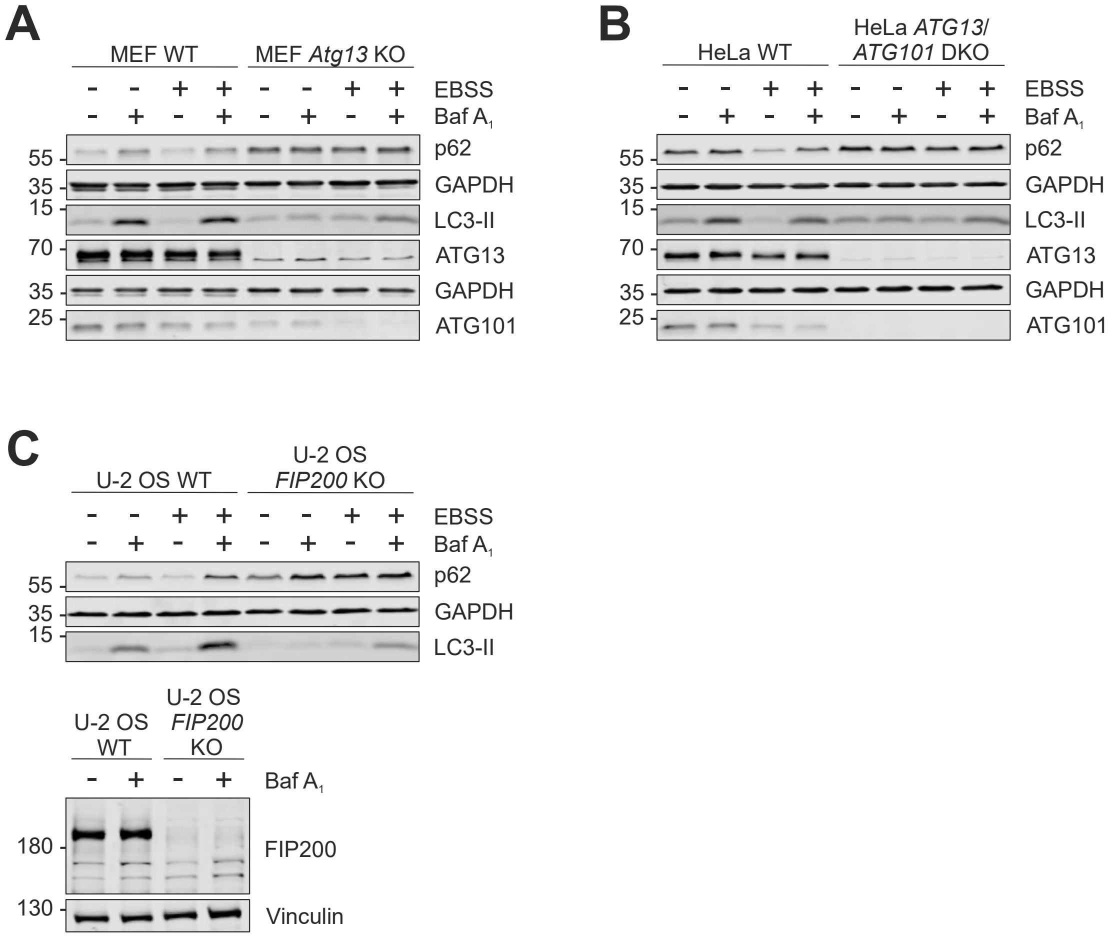

Suppl. Figure S11: Knockout of *Atg13/ATG13*, *ATG101* or *FIP200* severely impairs canonical autophagy in MEF, HeLa and U-2 OS cells. (**A**) MEF WT and MEF *Atg13* KO, (**B**) HeLa WT and HeLa *ATG13*/*ATG101* DKO and (**C**) U-2 OS WT and U-2 OS *FIP200* KO cells were treated with mono- or combination treatments (EBSS, 20 nM bafilomycin A_1) for 6 h. After treatment, the cells were lysed, and cellular lysates were immunoblotted for p62, LC3, GAPDH, FIP200, ATG13, ATG101 and Vinculin (n=1).

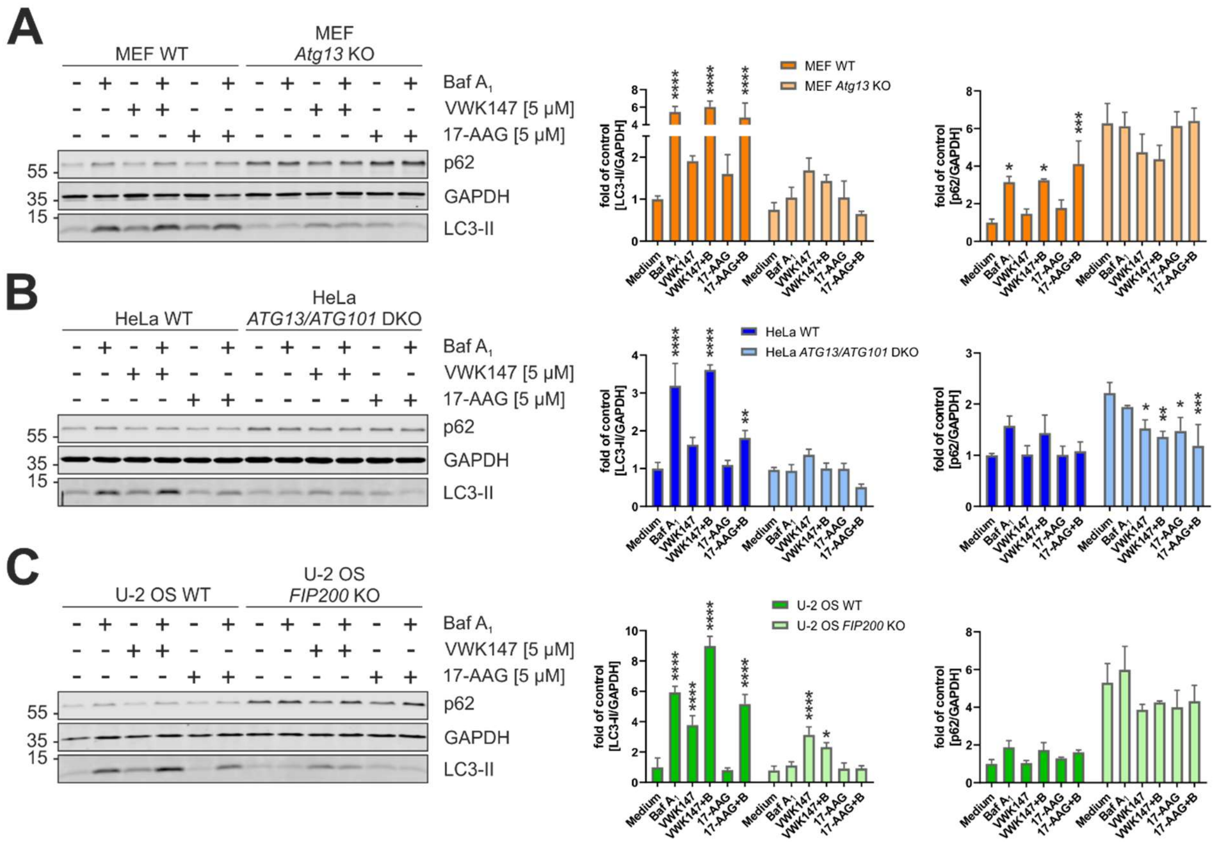

Suppl. Figure S12: 17-AAG does not induce non-canonical autophagy. (**A**) MEF WT and MEF *Atg13* KO, (**B**) HeLa WT and HeLa *ATG13/ATG101* DKO and (**C**) U-2 OS and U-2 OS *FIP200* KO were treated with 5 µM VWK147 or 17-AAG in presence or absence of 20 nM bafilomycin A_1 for 6 h. After treatment, the cells were lysed, and cellular lysates were immunoblotted for p62, LC3 and GAPDH. One representative immunoblot is shown. The quantifications of indicated ratios are from three independent experiments (means + SD). P values were determined by ordinary two-way ANOVA with Tukey´s multiple comparisons test (comparison to the solvent control of the respective cell line). *p ≤ 0.05; **p ≤ 0.01; ***p ≤ 0.001; ****p ≤ 0.0001.

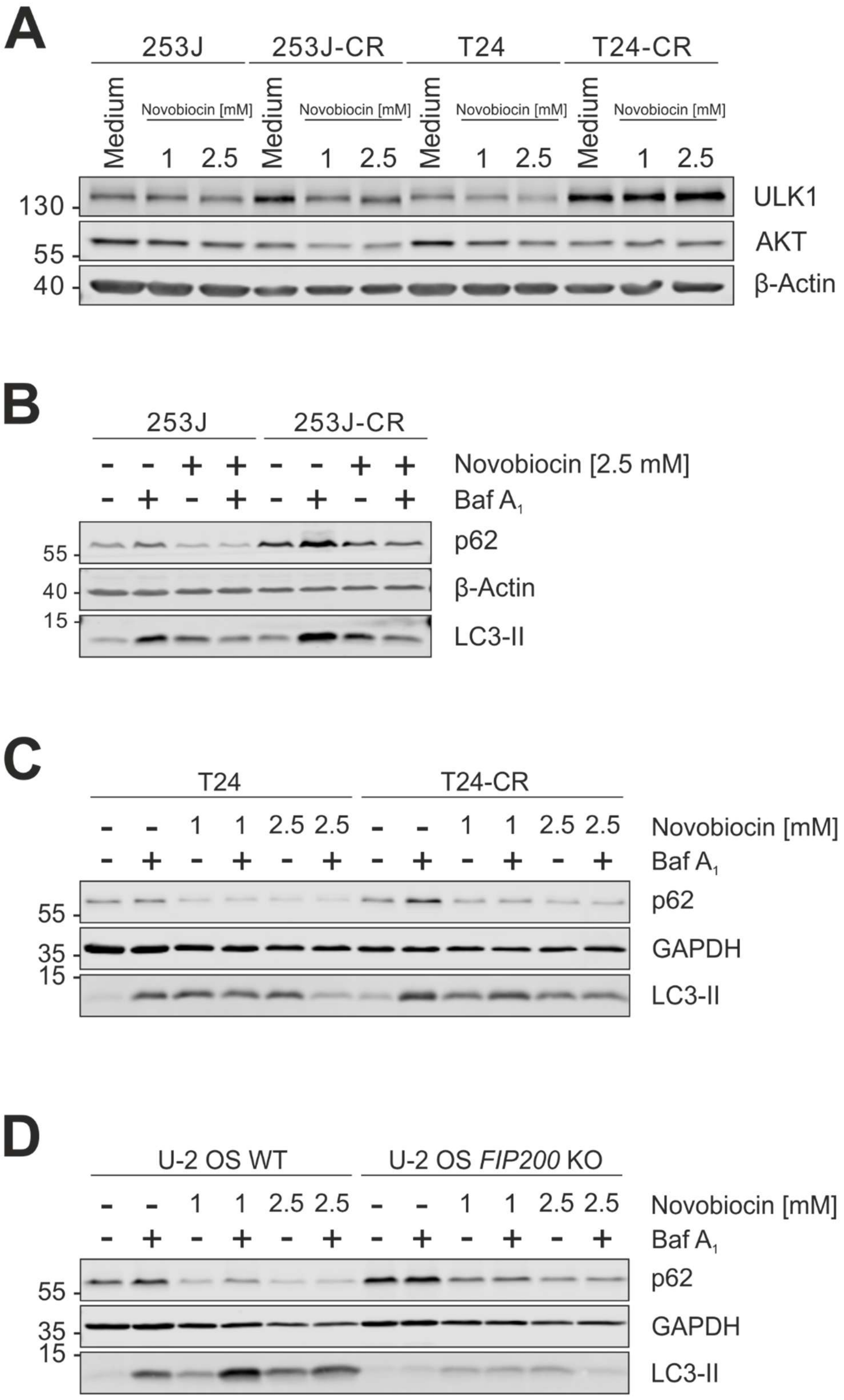

Suppl. Figure S13: Novobiocin induces non-canonical LC3 lipidation. (**A**) 253J, 253J-CR, T24 and T24-CR urothelial carcinoma cells were treated with indicated concentrations of Novobiocin for 6 h. After treatment, the cells were lysed, and cellular lysates were immunoblotted for ULK1, AKT and β-Actin.

One representative immunoblot is shown. (**B**) 253J, 253J-CR, (**C**) T24 and T24-CR urothelial carcinoma cells as well as (**D**) U-2 OS WT and U-2 OS *FIP200* KO osteosarcoma cells were treated with indicated mono- or combination treatments (1 mM or 2.5 mM Novobiocin, 20 nM bafilomycin A_1) for 6 h. After treatment, the cells were lysed, and cellular lysates were immunoblotted for p62, LC3 and β-Actin. One representative immunoblot is shown (n=3).
